# Supplementary figures and images for: Strong evidence for the adaptive walk model of gene evolution in Drosophila and Arabidopsis
Source: PLoS Biol. 2022 Sep 13;20(9):e3001775. doi: 10.1371/journal.pbio.3001775 (PMC9470001; doi:10.1371/journal.pbio.3001775)

**(a)**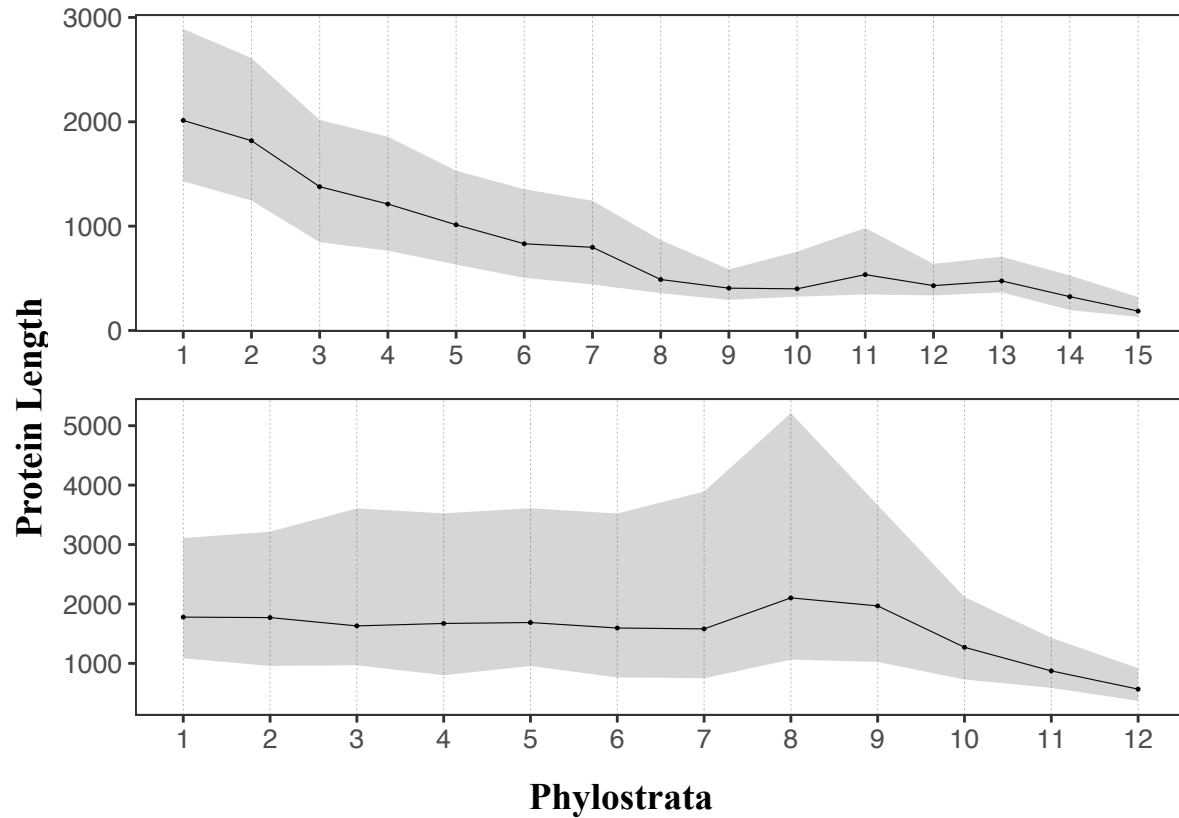**(b)**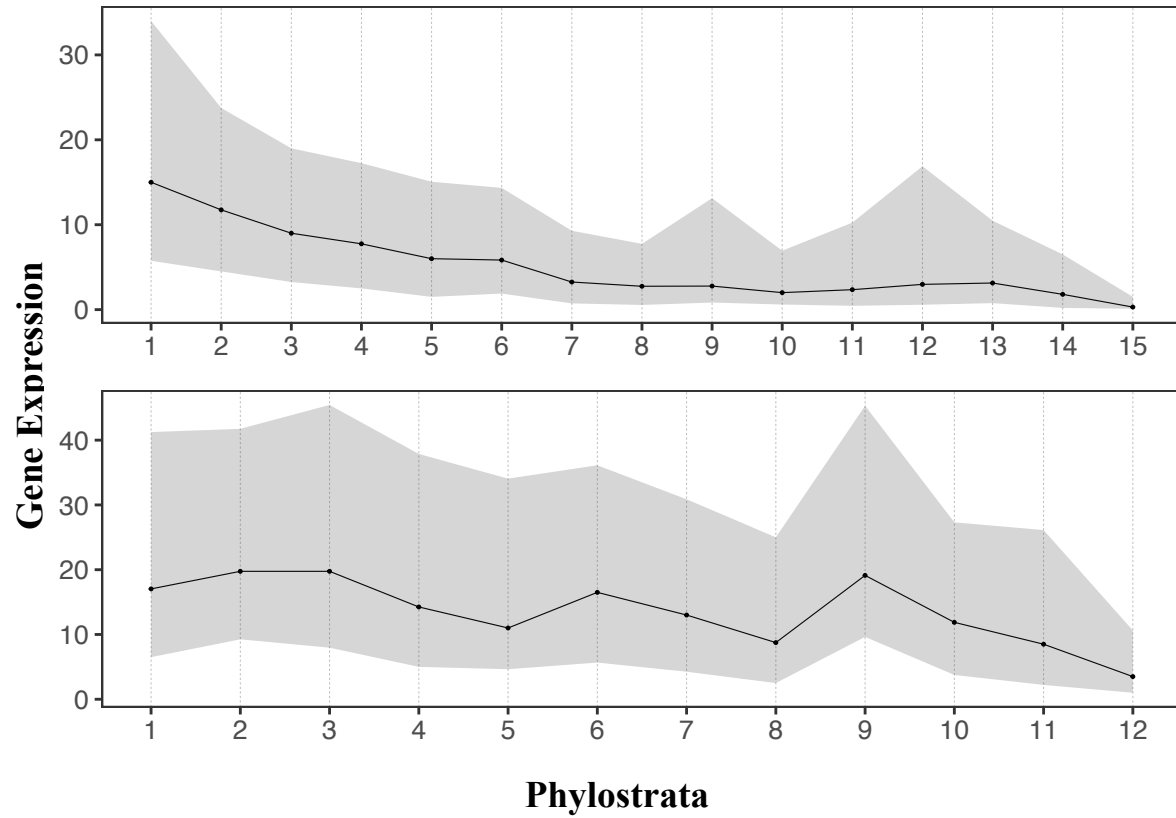

Supplement: S1 Fig — Relationship between gene age and gene length (a) and gene expression (b) for A. thaliana (top) and D. melanogaster (bottom). This analysis was performed by categorizing gene age according to the clades defined in Fig 1A. For each clade, the median value of gene length and gene expression is depicted with the black dot. The shaded area represents the values of gene length and mean expression levels within the first and third quartile. The data (S2 and S3 Data tables) and code needed to generate this figure can be found at https://gitlab.gwdg.de/molsysevol/supplementarydata_geneage and https://zenodo.org/record/6828430. (PDF) [file pbio.3001775.s005.pdf]

**(a)**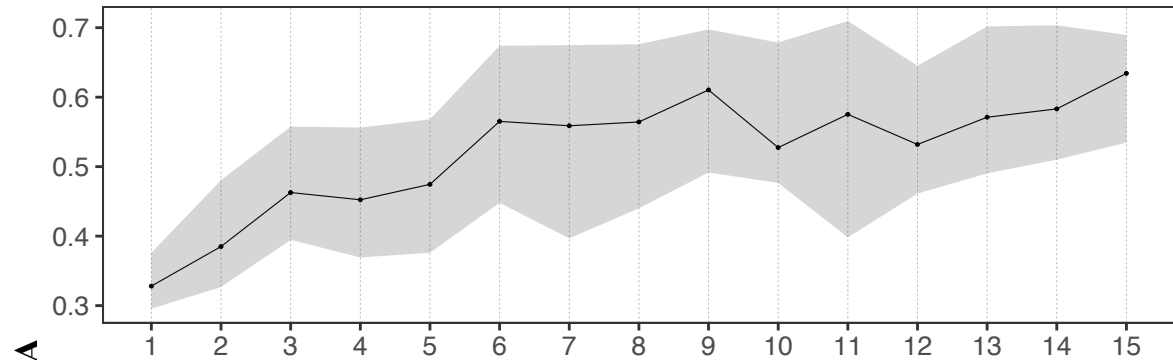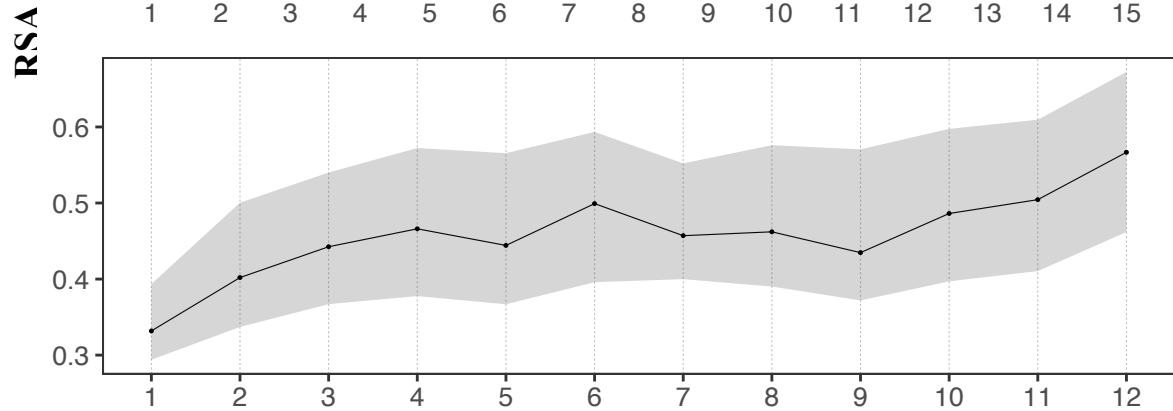**Phylostrata****(b)**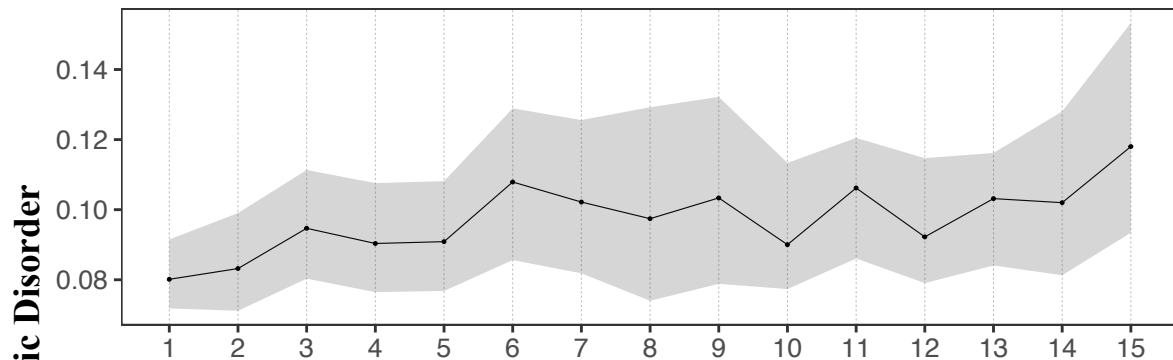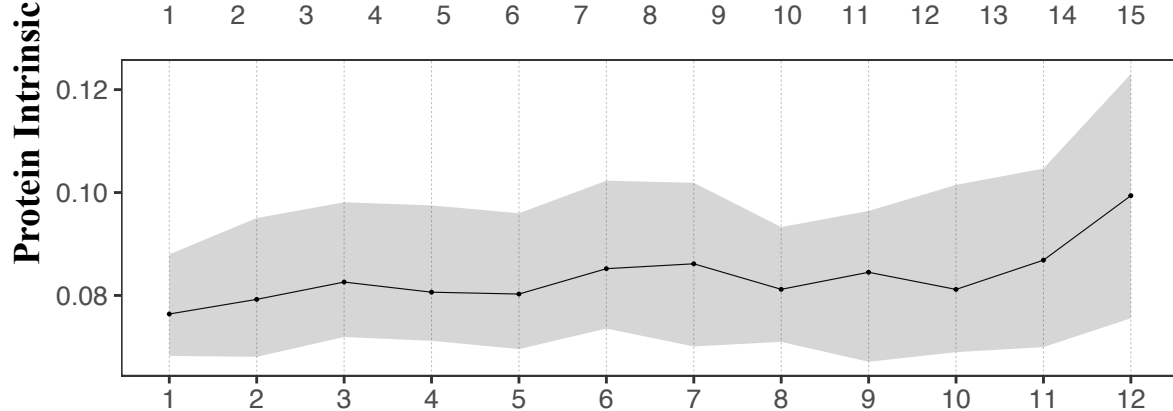**Phylostrata**

Supplement: S2 Fig — Relationship between gene age and RSA (a) and protein intrinsic disorder (b) for A. thaliana (top) and D. melanogaster (bottom). Legend as in S1 Fig. The data (S4 and S5 Data tables) and code needed to generate this figure can be found at https://gitlab.gwdg.de/molsysevol/supplementarydata_geneage and https://zenodo.org/record/6828430. (PDF) [file pbio.3001775.s006.pdf]

**(a)**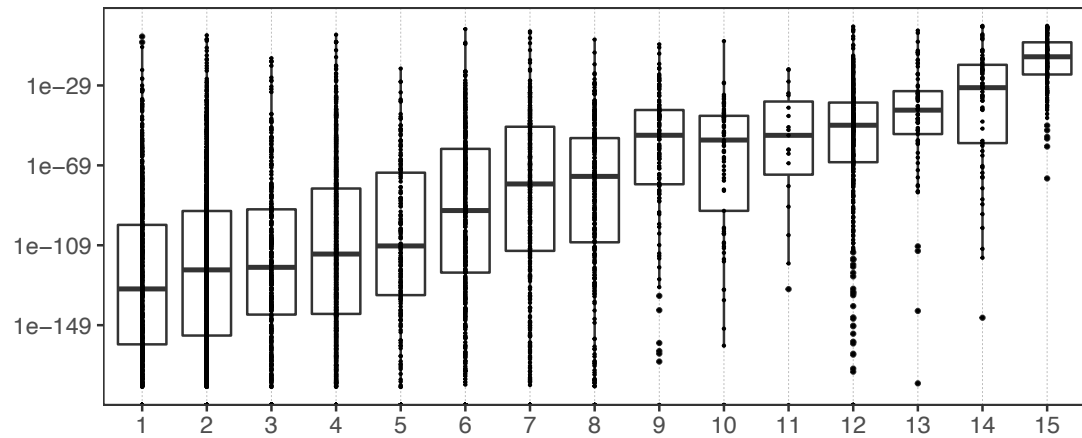**E-value**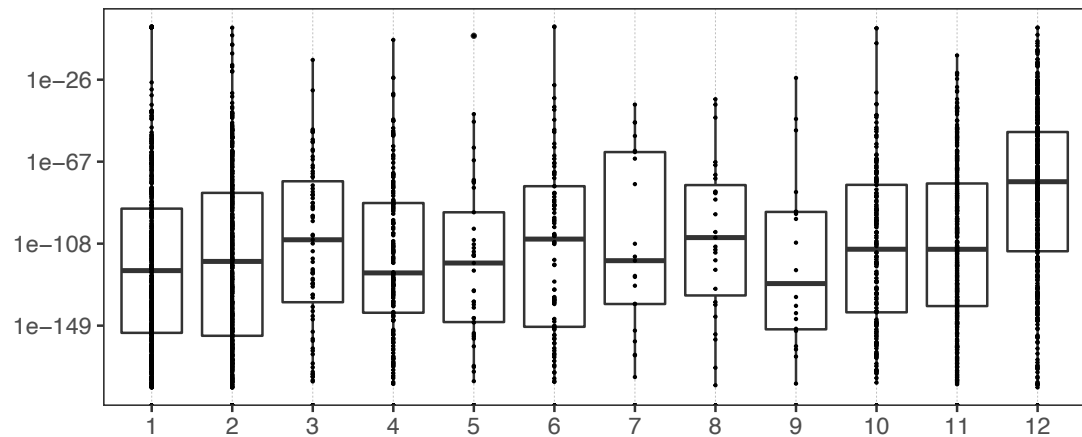**Phylostrata****(b)**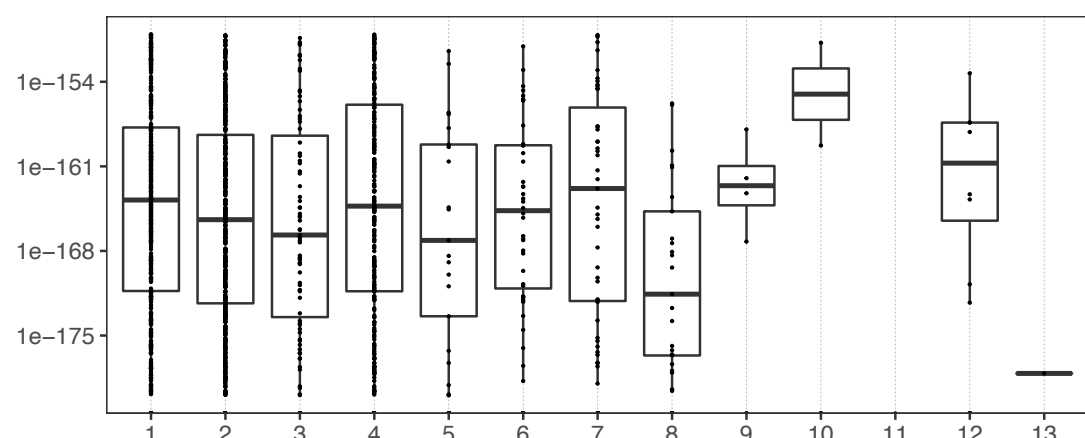**E-value**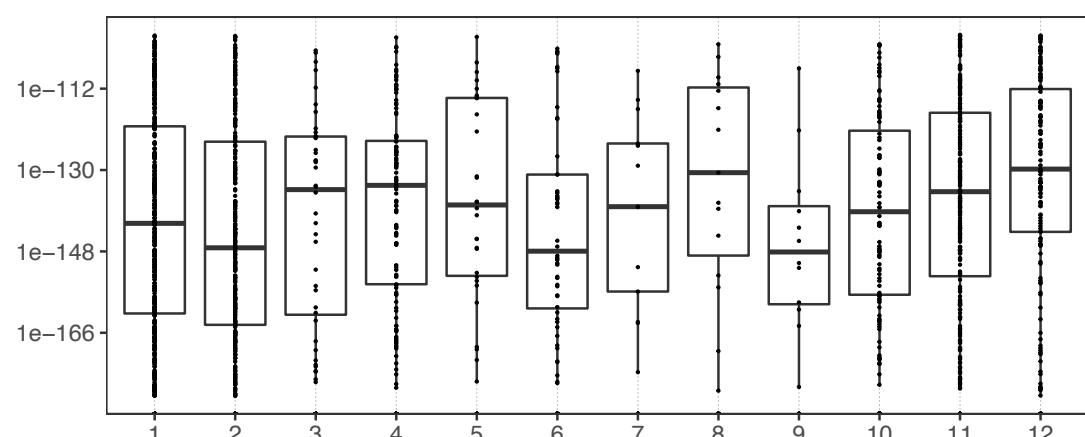**Phylostrata**

Supplement: S3 Fig — Relationship between gene age and E-values before (a) and after (b) the E value correction for A. thaliana (top) and D. melanogaster (bottom). Each black dot represents a gene and median E value for each clade is represented with the black line in the boxplot. The data (S23 Data) and code needed to generate this figure can be found at https://gitlab.gwdg.de/molsysevol/supplementarydata_geneage and https://zenodo.org/record/6828430. (PDF) [file pbio.3001775.s007.pdf]

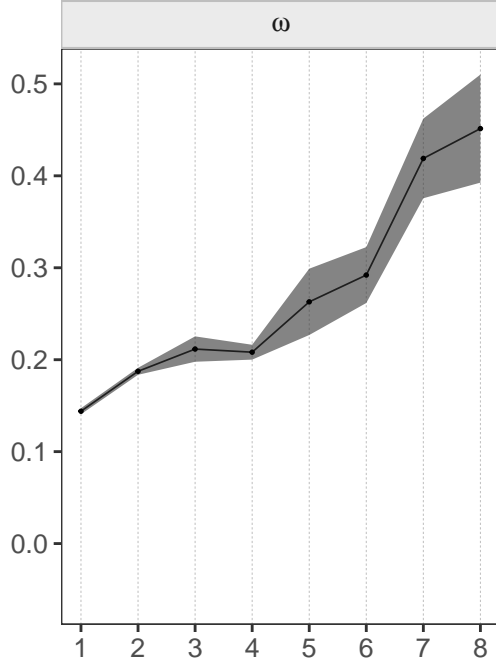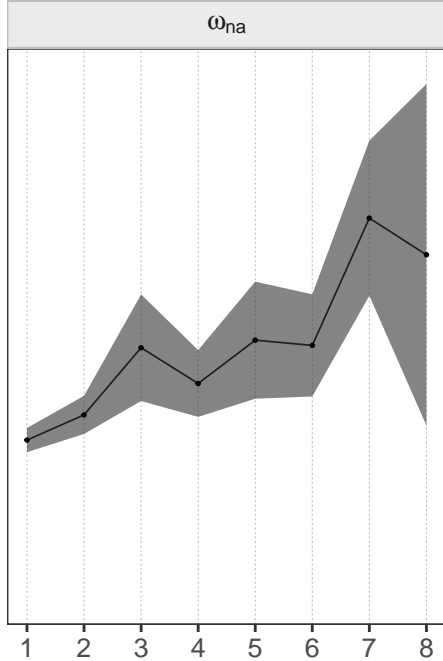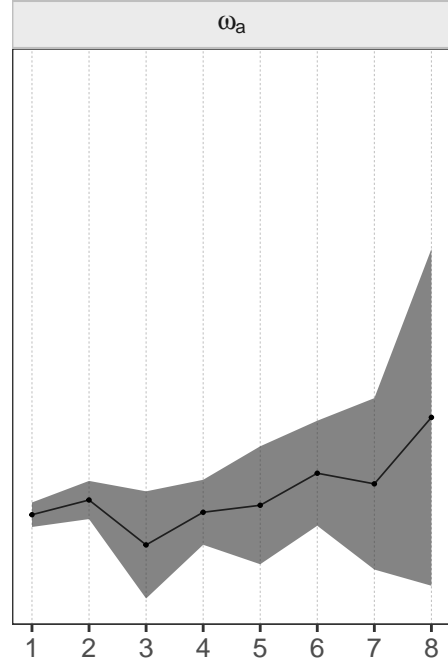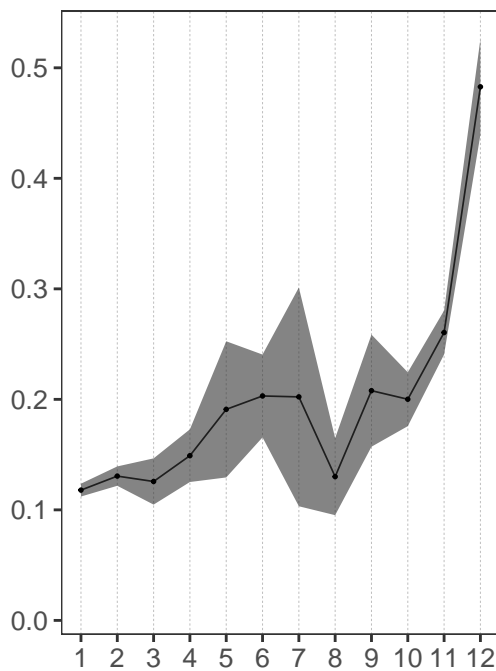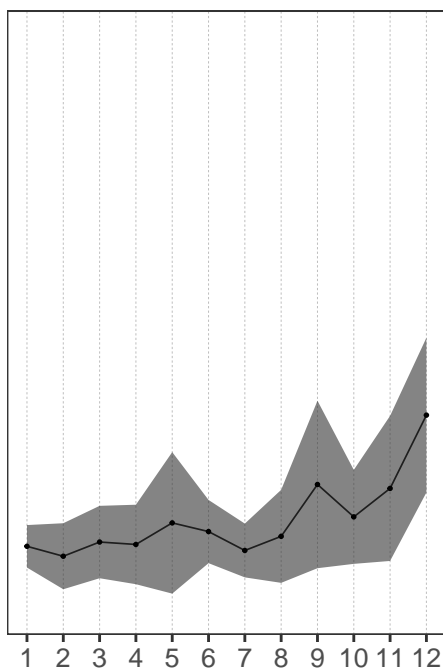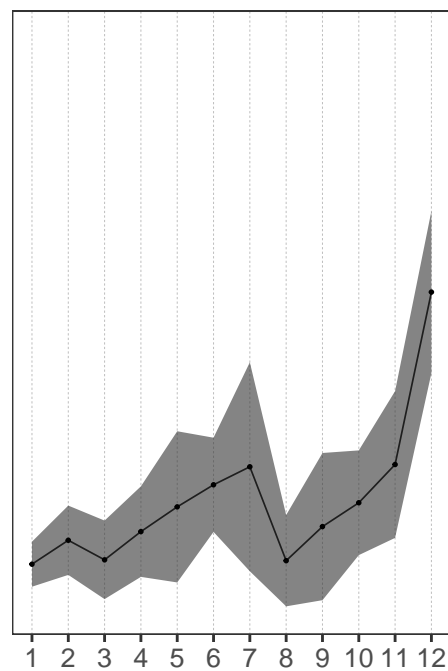

**Phylostrata Group**

Supplement: S4 Fig — Mean values of ω, ωna, and ωa for each category are represented with the black points. Error bars denote for the 95% confidence interval for each category, computed over 100 bootstrap replicates. The data (S24 Data) and code needed to generate this figure can be found at https://gitlab.gwdg.de/molsysevol/supplementarydata_geneage and https://zenodo.org/record/6828430. (PDF) [file pbio.3001775.s008.pdf]

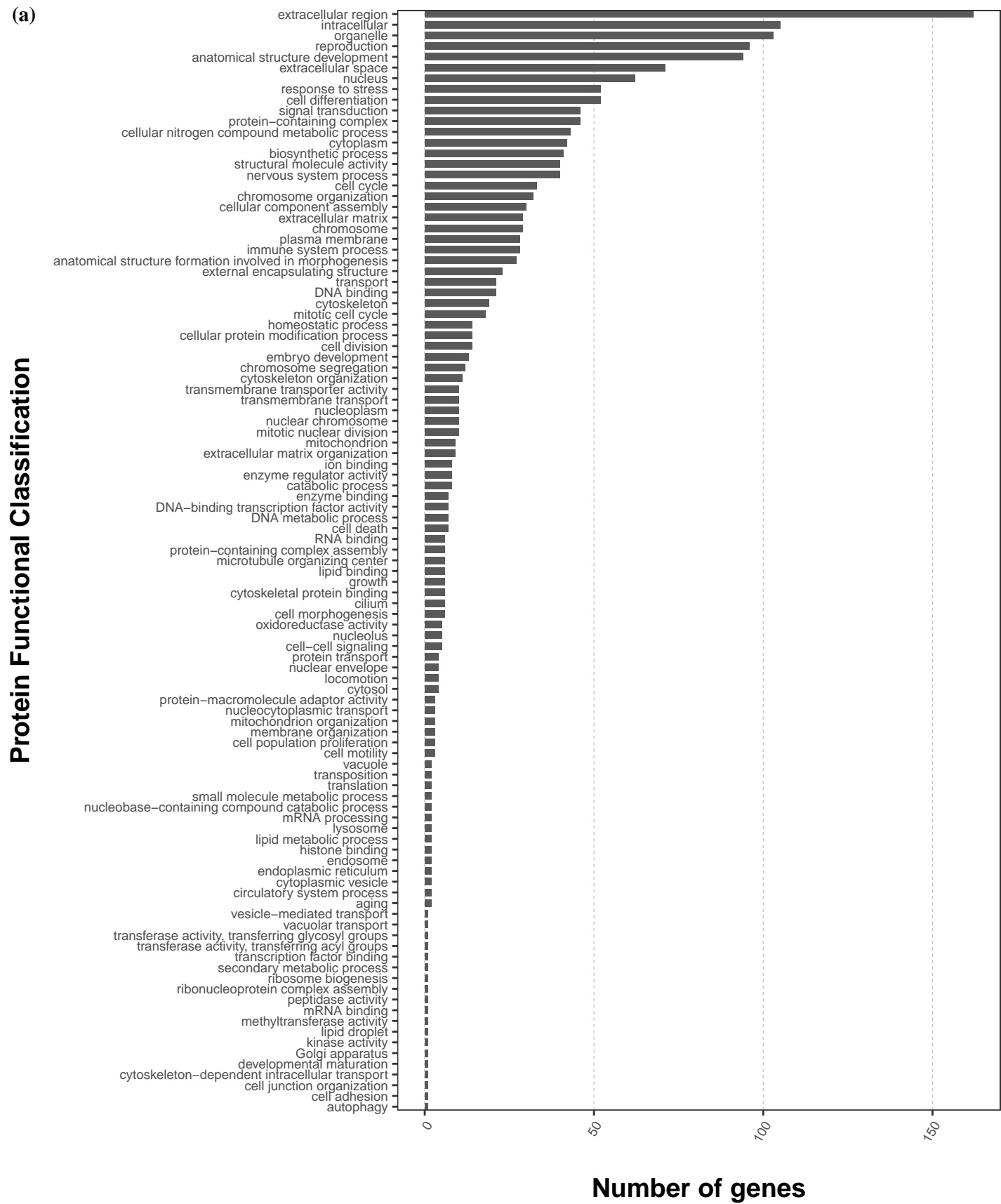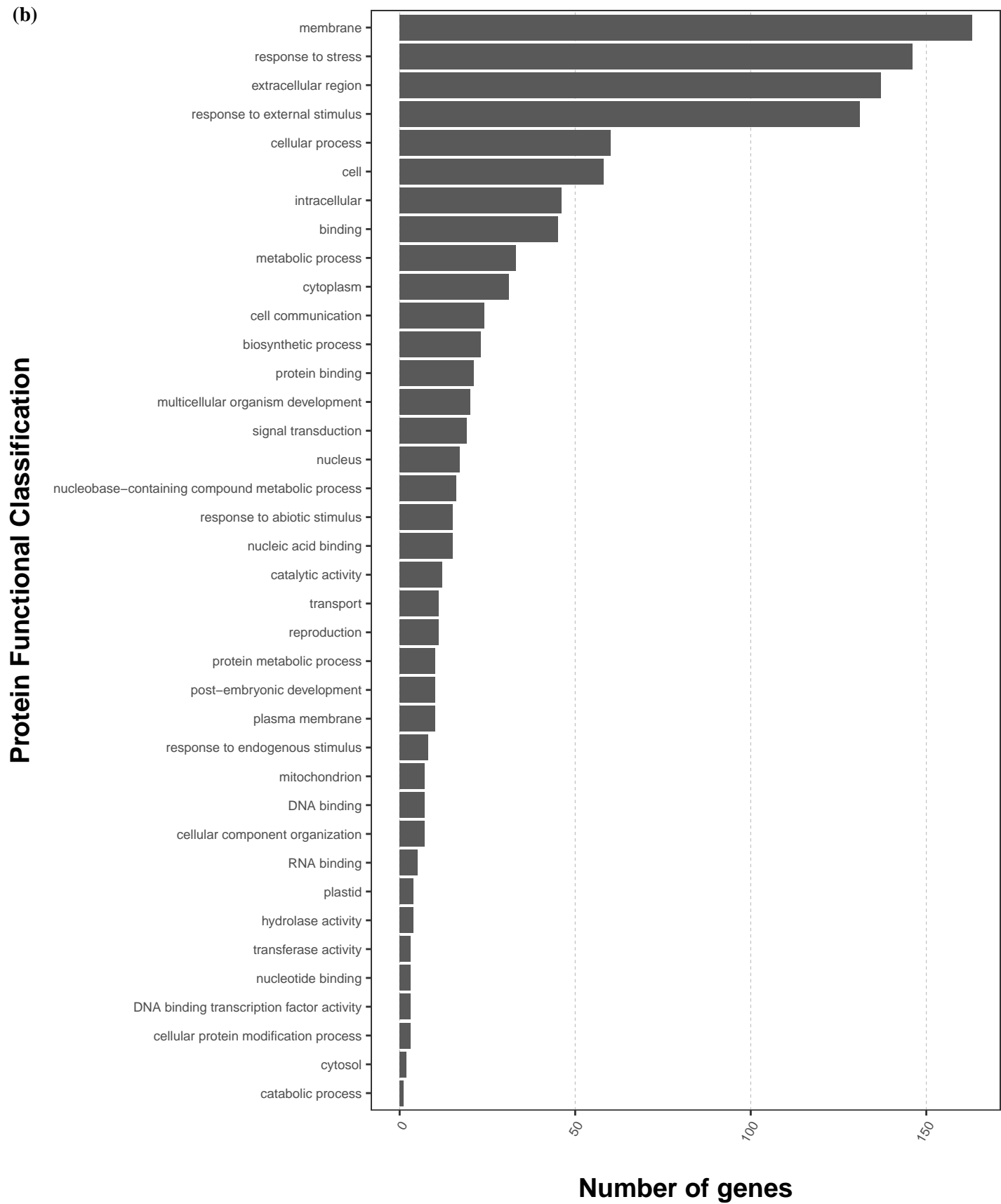

Supplement: S5 Fig — The number of young genes for the respective protein function in (a) D. melanogaster and (b) A. thaliana. The data (S25 Data) and code needed to generate this figure can be found at https://gitlab.gwdg.de/molsysevol/supplementarydata_geneage and https://zenodo.org/record/6828430. (PDF) [file pbio.3001775.s009.pdf]

**a)**

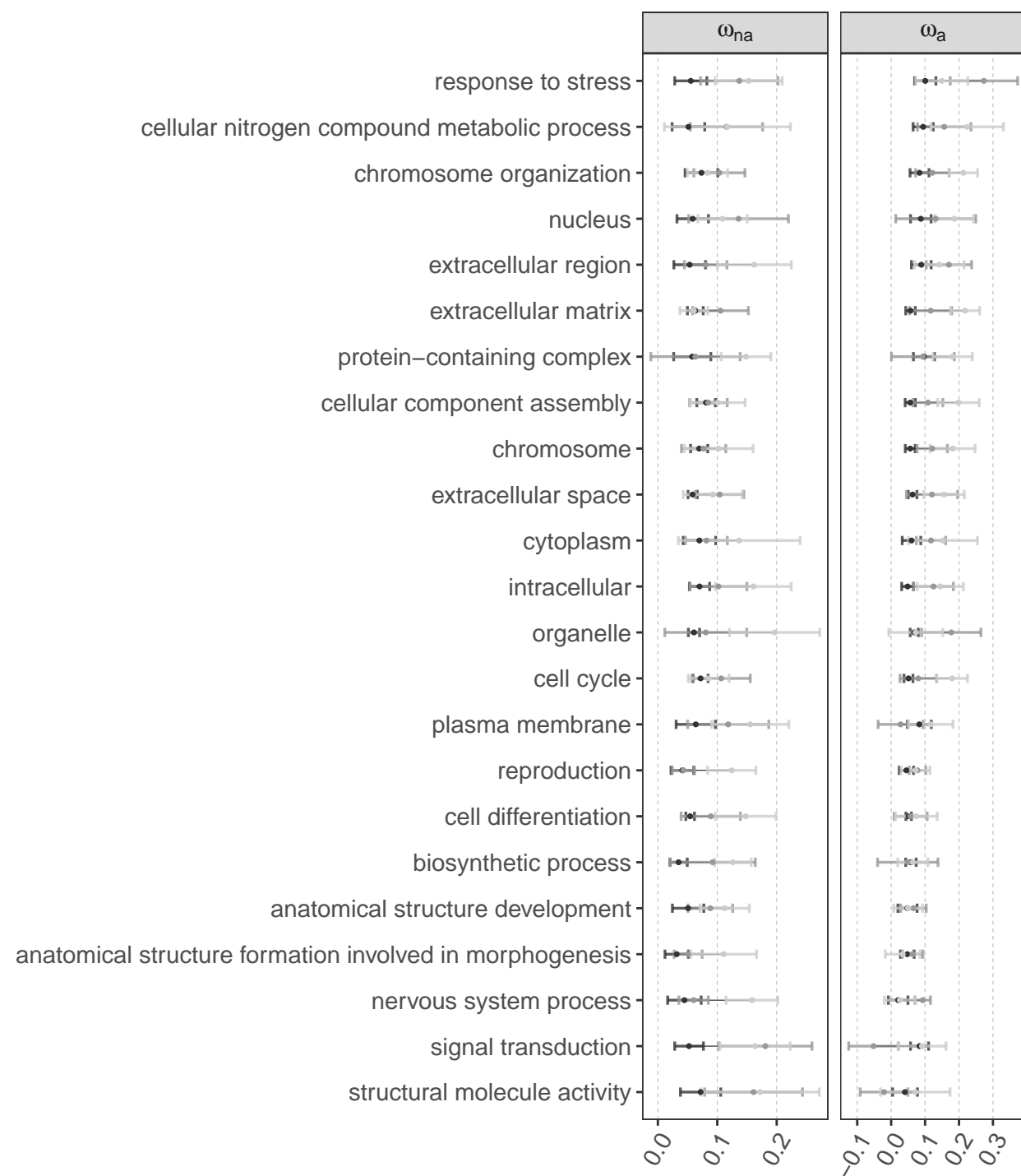

**b)**

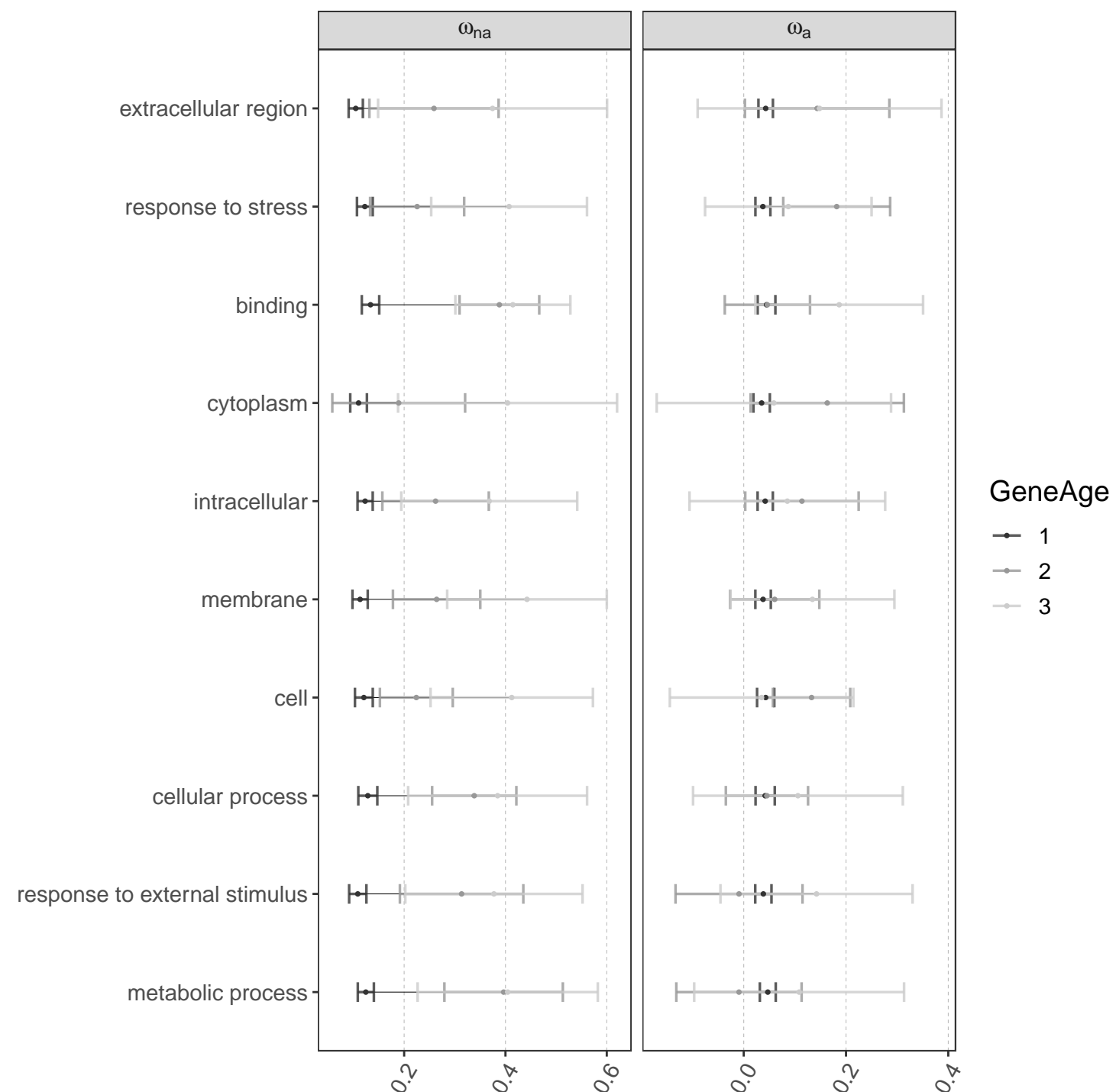

Supplement: S6 Fig — Estimates of ω, ωna, and ωa plotted as a function of protein function and gene age in (a) A. thaliana and (b) D. melanogaster. Categories are ordered according to the values of ωa. Gene age categories are ordered from old (1) to young (3). Mean values of ω, ωna, and ωa for each class are represented with the black points. Error bars denote the 95% confidence interval for each category, computed over 100 bootstrap replicates. The data (S26 Data) and code needed to generate this figure can be found at https://gitlab.gwdg.de/molsysevol/supplementarydata_geneage and https://zenodo.org/record/6828430. (PDF) [file pbio.3001775.s010.pdf]

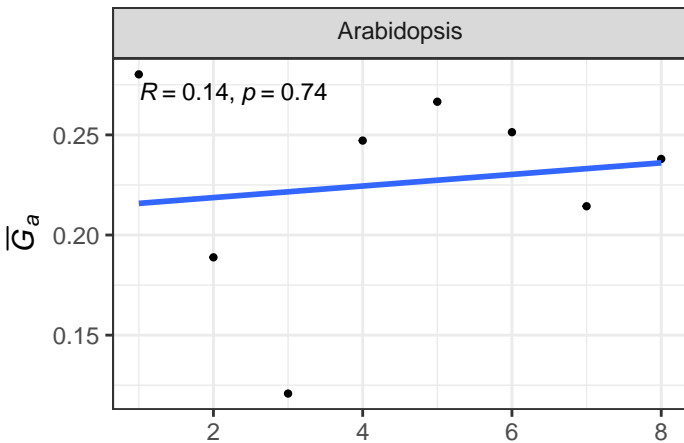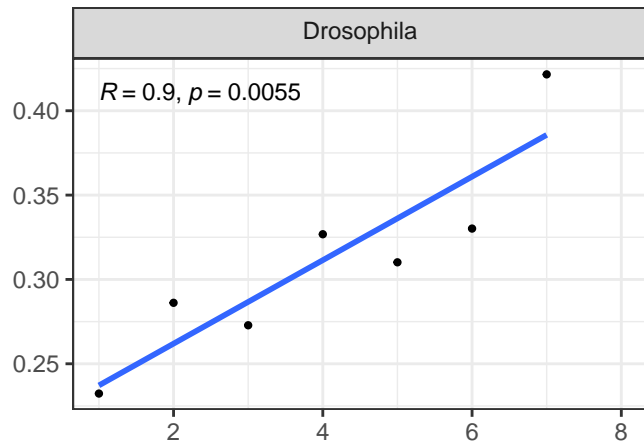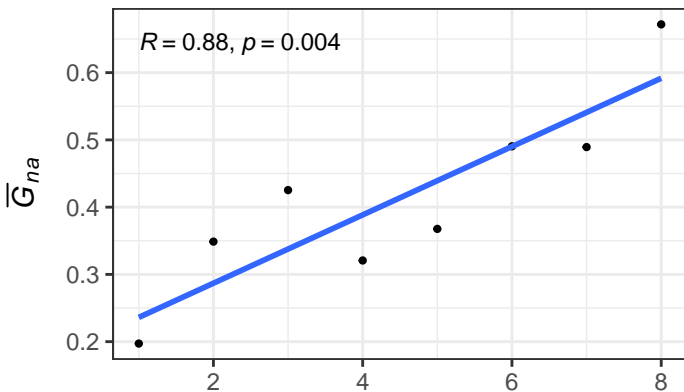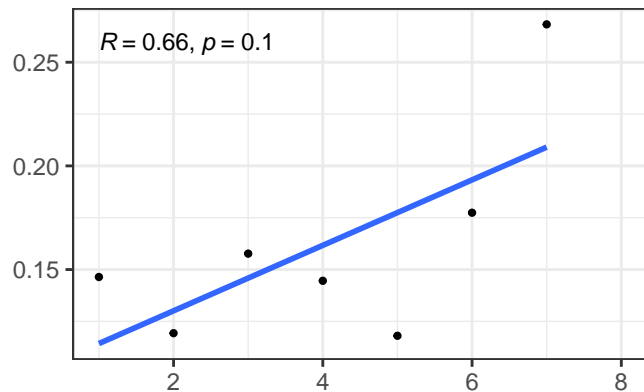

Phylostrata level

Supplement: S7 Fig — Each point represents the weighted average for each age category. A linear model was fitted between gene age and Grantham’s distances values and is represented with the blue line. Statistical significance was assessed with a Pearson’s correlation test and the respective correlation coefficient (R) and p-values (p) are shown in each plot. The data (S28 Data) and code needed to generate this figure can be found at https://gitlab.gwdg.de/molsysevol/supplementarydata_geneage and https://zenodo.org/record/6828430. (PDF) [file pbio.3001775.s011.pdf]

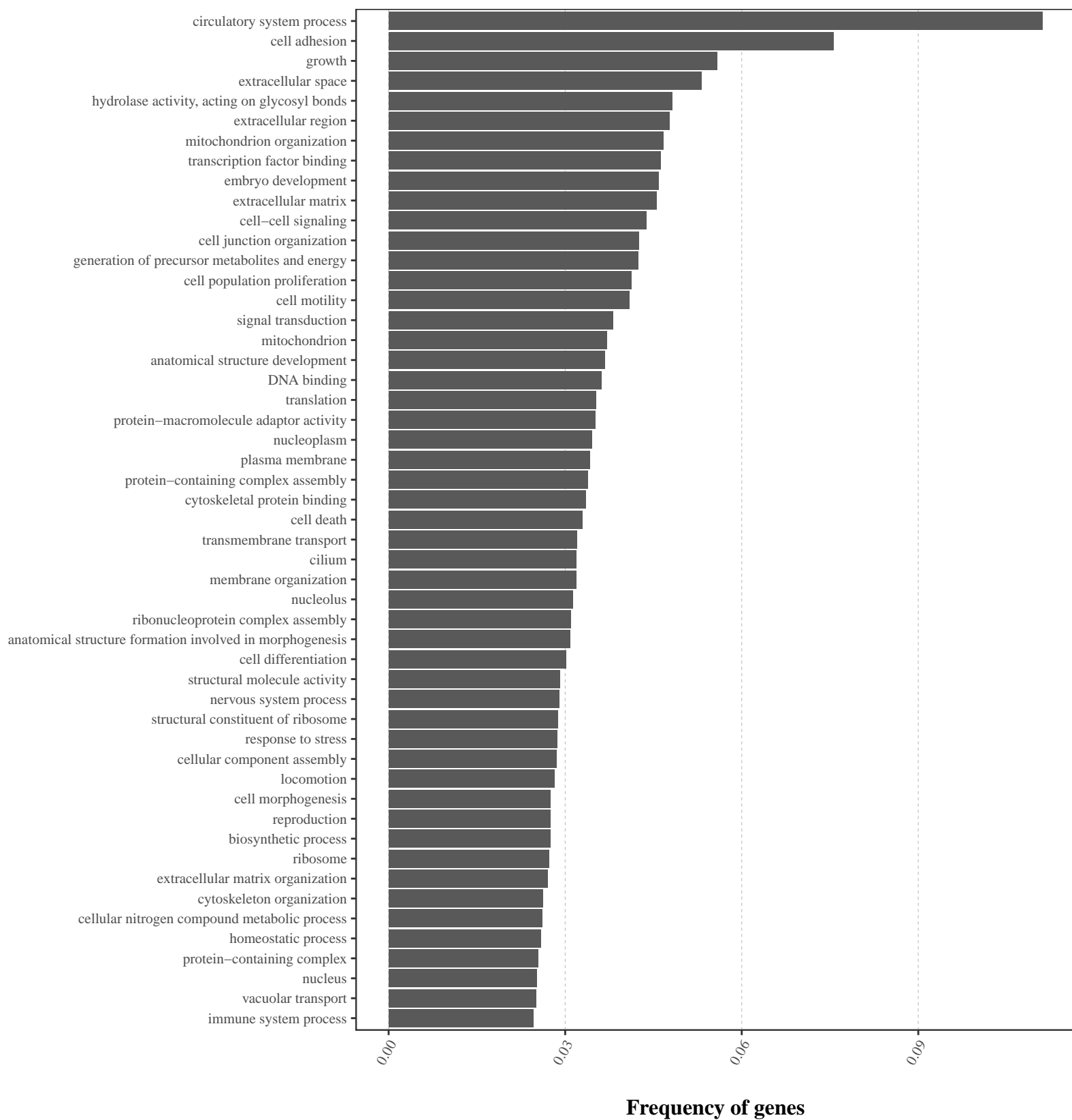

Supplement: S8 Fig — The frequency of genes for each functional category was estimated by dividing the number of genes present in these clades by the total number of genes annotated with the respective function. The data (S25 Data) and code needed to generate this figure can be found at https://gitlab.gwdg.de/molsysevol/supplementarydata_geneage and https://zenodo.org/record/6828430. (PDF) [file pbio.3001775.s012.pdf]

Statistical group

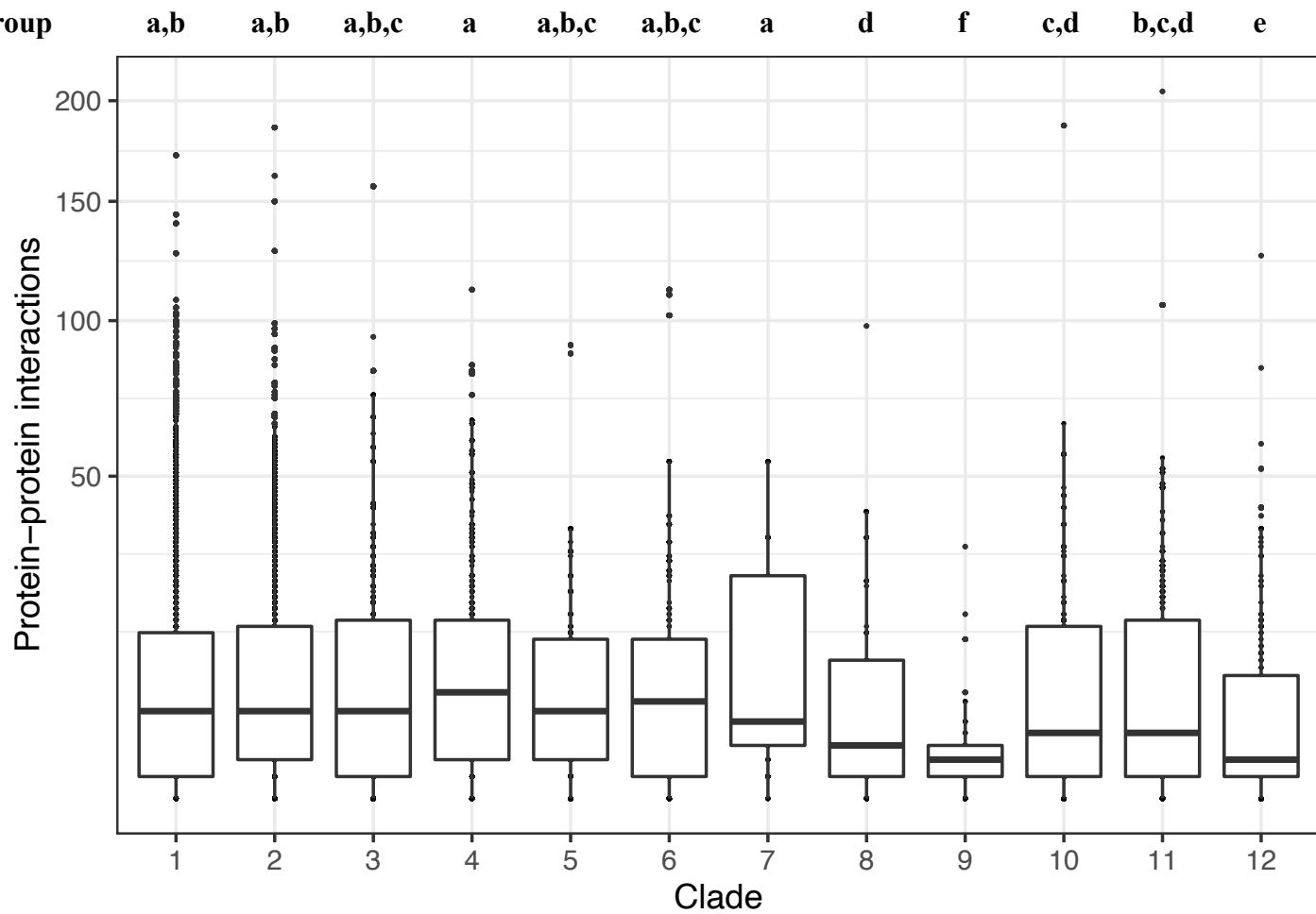

Supplement: S9 Fig — The statistical group for each clade is represented. The black line represents the median value of PPI for each clade and black dots denote the outliers of the distribution. The y-axis is scaled with a square root function. The data (S29 Data) and code needed to generate this figure can be found at https://gitlab.gwdg.de/molsysevol/supplementarydata_geneage and https://zenodo.org/record/6828430. (PDF) [file pbio.3001775.s013.pdf]

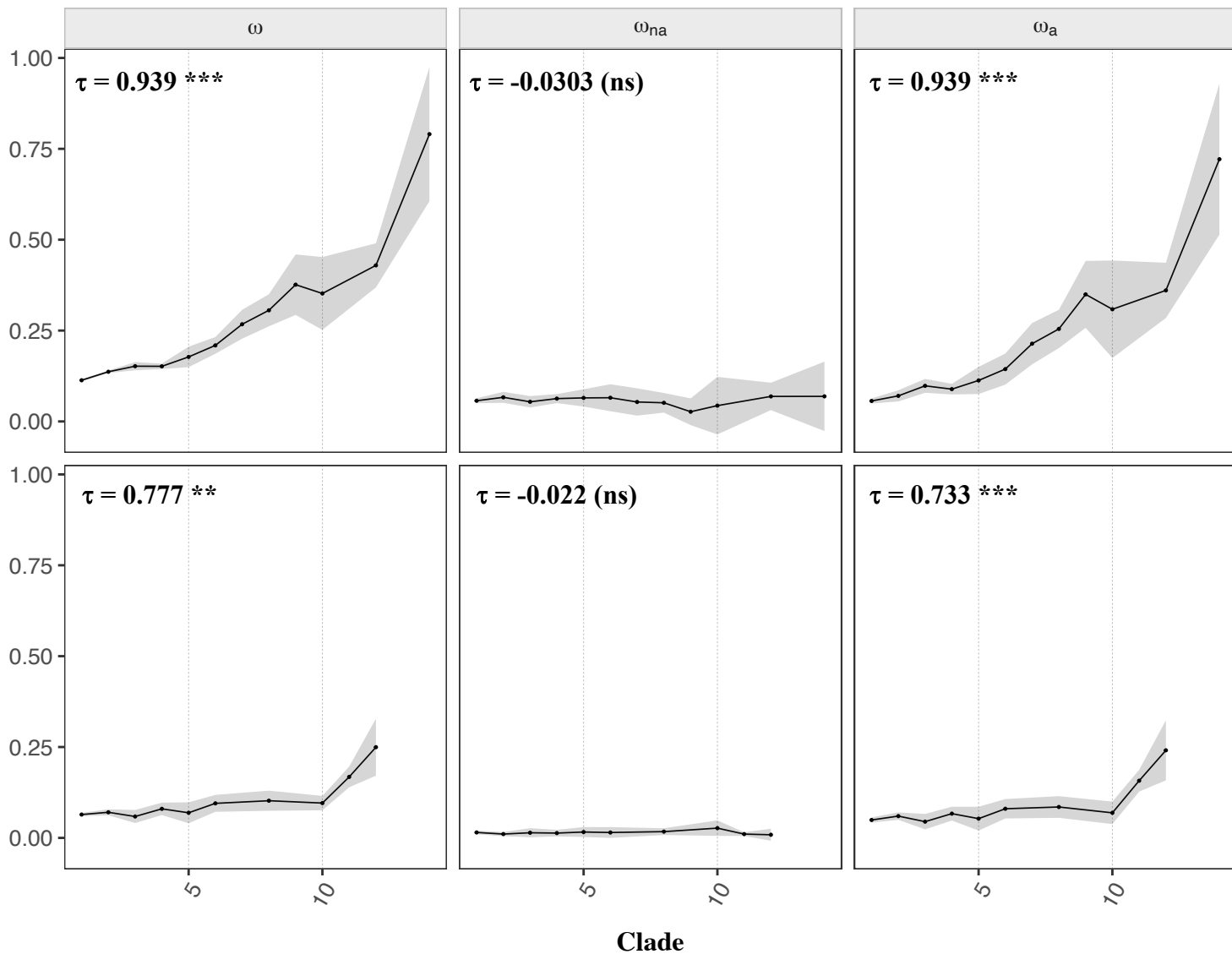

Supplement: S10 Fig — The Kendall’s correlation coefficients are shown with the respective significance (*P < 0.05; **P < 0.01; ***P < 0.001; “.” 0.05 ≤ P < 0.10). Legend as in S4 Fig. The data (S30 Data) and code needed to generate this figure can be found at https://gitlab.gwdg.de/molsysevol/supplementarydata_geneage and https://zenodo.org/record/6828430. (PDF) [file pbio.3001775.s014.pdf]
