# Supplementary material for: Strong evidence for the adaptive walk model of gene evolution in Drosophila and Arabidopsis
Source: PLoS Biol. 2022 Sep 13;20(9):e3001775. doi: 10.1371/journal.pbio.3001775 (PMC9470001; doi:10.1371/journal.pbio.3001775)
Supplement: S1 File — (PDF) [file pbio.3001775.s047.pdf]

Gene Age Analysis

af_moutinho

23/04/2021

This script includes the analysis performed with gene age alone.

```
setwd("~/Dropbox/SupplementaryData_GeneAge/")
# change here to the respective folder where you keep the data

# Libraries
library(plyr)
library(dplyr)
library(data.table)
library(ggplot2)
library(reshape2)
library(doBy)
library(knitr)
library(kableExtra)
#

# theme of the plot
theme.plot <- function(x) {
  theme(axis.title = element_text(face = "bold", color = "black", size=14),
        text = element_text(size=14),
        axis.title.x = element_text(margin = margin(t = 18, r = 10, b = 0, l = 0)),
        axis.title.y = element_text(margin = margin(t = 18, r = 10, b = 0, l = 0)),
        panel.grid.minor=element_blank(),
        panel.grid.major = element_line(colour = "grey", linetype = "dashed", size = 0.2),
        panel.grid.major.y=element_blank(),
        #strip.text.y = element_blank(),
        axis.text.x = element_text(angle = 60, hjust = 1))
}

age_df <- read.table(file = "S1_Data.csv", sep = "\t", header = TRUE)

# arranging the table for plotting:
age_df2 <- melt(age_df, id.vars = c("GeneAge", "chr", "species"),
       measure.vars = c("dnds", "omegaNA", "omegaA"))

# function to estimate the mean and standard deviation to plot the results with the
# mean of the bootstrap replicates and the 95% confidence interval
fun <- function(x){
  c(mean=mean(x), sd=sd(x), var=var(x))
}

tbl.sum <- summaryBy(value ~ variable + GeneAge + chr + species, data=age_df2, FUN = fun)
```

```r
inter_class <- summaryBy(value ~ variable + chr + species, data=age_df2, FUN = fun)

# change the variable name to the respective symbol:
tbl.sum$variable <- factor(tbl.sum$variable, levels = c("dnds", "omegaNA", "omegaA"))
levels(tbl.sum$variable) <- c(expression(omega), expression(omega[na]),
                                    expression(omega[a]))

# plotting:
plot_age <- ggplot(tbl.sum, aes(x = GeneAge, y = value.mean,  fill = chr)) +
  geom_line(col = "black", size = 0.3) +
  geom_ribbon(aes(ymin=value.mean - 1.96*value.sd,
                  ymax=value.mean + 1.96*value.sd,  fill = chr), alpha=0.6) +
  geom_point(size=1, aes(shape = chr))+
  facet_grid(species~variable, scales = "free_x", labeller = label_parsed) +
  ylab("") +
  xlab("Phylostrata") +
  scale_fill_grey() +
  scale_color_grey() +
  scale_x_continuous(limits = c(1,15), breaks = c(1,3,5,7,9,11,13,15)) +
  theme_bw() +
  theme.plot()
plot_age
```

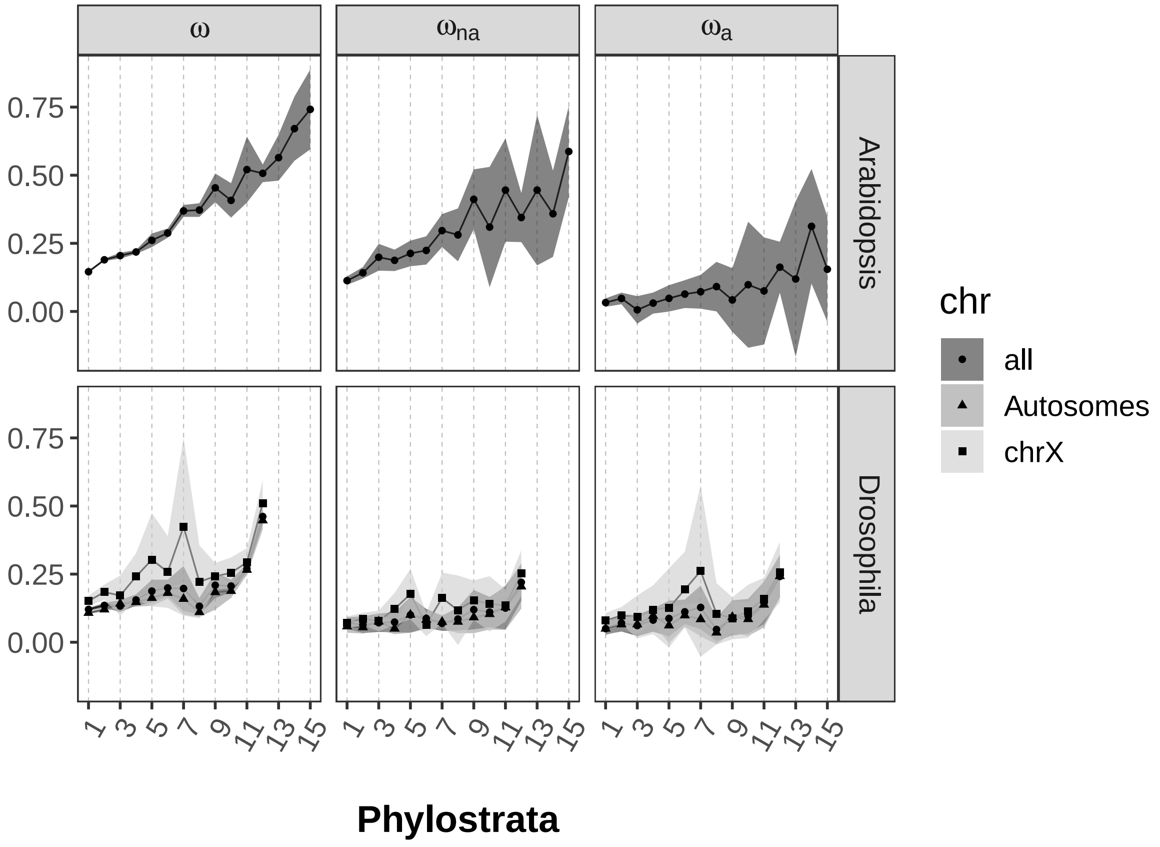

This section shows how the statistical analyses were performed.

Table 1: Statistics for the analysis of gene age

chr	species	variable	Kendall.tau	p.value
all	Arabidopsis	omega	0.9619048	0.0000006
all	Arabidopsis	omega[na]	0.8476190	0.0000106
all	Arabidopsis	omega[a]	0.7333333	0.0001387
all	Drosophila	omega	0.7272727	0.0009966
all	Drosophila	omega[na]	0.6969697	0.0016086
all	Drosophila	omega[a]	0.6363636	0.0039762
Autosomes	Drosophila	omega	0.7575758	0.0006066
Autosomes	Drosophila	omega[na]	0.6363636	0.0039762
Autosomes	Drosophila	omega[a]	0.4848485	0.0282123
chrX	Drosophila	omega	0.5757576	0.0091672
chrX	Drosophila	omega[na]	0.4242424	0.0548539
chrX	Drosophila	omega[a]	0.4242424	0.0548539

```
tbl.stat <- ddply(tbl.sum, c("chr", "species", "variable"), function(x) {
    var <- as.numeric(factor(x$GeneAge))
    variable.value <- as.numeric(factor(x$value.mean))
    corr = cor.test(var, variable.value, method = "kendall", exact = FALSE)
    Kendall.tau = corr$estimate
    p.value = corr$p.value
    dat = data.frame(Kendall.tau, p.value)
    })

# presenting the table:
kable(x = tbl.stat, caption = "Statistics for the analysis of gene age")
```

The last chunk of the script contains the analysis performed to verify the effect of the chromosome in the data.

```
# Libraries
library(lmtest)
#

# subsetting the data for omega a and omega na in Drosophila
dmel.oa <- subset(tbl.sum, species == "Drosophila" & variable == "omega[a]" & !chr == "all")
dmel.ona <- subset(tbl.sum, species == "Drosophila" & variable == "omega[na]" & !chr == "all")

## omega_a:
# M1 without chromosome effect:
lm1.dmel.oa <- lm(value.mean ~ GeneAge, dmel.oa)
# M2 with chromosome effect:
lm2.dmel.oa <- lm(value.mean ~ GeneAge * chr, dmel.oa)
a.oa <- anova(lm1.dmel.oa, lm2.dmel.oa)
#Model 1: value.mean ~ GeneAge
#Model 2: value.mean ~ GeneAge * chr
#  Res.Df      RSS Df Sum of Sq      F  Pr(>F)
#1     22 0.066364
#2     20 0.052373  2  0.013991 2.6714 0.09371 .
```

```
## omega_na:
# M1 without chromosome effect:
lm1.dmel.ona <- lm(value.mean ~ GeneAge, dmel.ona)
# M2 with chromosome effect:
lm2.dmel.ona <- lm(value.mean ~ GeneAge * chr, dmel.ona)
a.ona <- anova(lm1.dmel.ona, lm2.dmel.ona)
# Model 1: value.mean ~ GeneAge
#Model 2: value.mean ~ GeneAge * chr
#  Res.Df      RSS Df Sum of Sq     F  Pr(>F)
#1     22 0.033238
#2     20 0.024177  2 0.0090615 3.748 0.04146 *
```
