# Supplementary material for: Strong evidence for the adaptive walk model of gene evolution in Drosophila and Arabidopsis
Source: PLoS Biol. 2022 Sep 13;20(9):e3001775. doi: 10.1371/journal.pbio.3001775 (PMC9470001; doi:10.1371/journal.pbio.3001775)
Supplement: S2 File — (PDF) [file pbio.3001775.s048.pdf]

e-value_correction

af_moutinho

20/04/2021

This script includes the analysis of gene age correcting for BLAST's false negative rate.

```
setwd("~/Dropbox/SupplementaryData_GeneAge/Data/")
# change path to where you keep the data

# Libraries
library(plyr)
library(dplyr)
library(reshape2)
library(ggplot2)
library(data.table)
library(doBy)
library(data.table)
library(cowplot)
library(knitr)
#library(kableExtra)
#

# getting the data tables:
# Drosophila:
dmel.df <- read.table(file = "S31_Data.csv", sep = "\t", header = TRUE)
# Arabidopsis:
arab.df <- read.table(file = "S32_Data.csv", sep = "\t", header = TRUE)

# keeping only the GeneID and evalue columns:
# Drosophila:
sub.dmel.df <- subset(dmel.df, select = c("GeneID", "Clade", "evalue"))
sub.dmel.df$species <- rep("Drosophila", nrow(sub.dmel.df))
# keeping unique genes as the data has duplicates due to GO annotations
sub.dmel.df <- unique(sub.dmel.df)
# Arabidopsis:
sub.arab.df <- subset(arab.df, select = c("GeneID", "Clade", "evalue"))
sub.arab.df$species <- rep("Arabidopsis", nrow(sub.arab.df))
# keeping unique genes as the data has duplicates due to GO annotations
sub.arab.df <- unique(sub.arab.df)

age.evalue <- rbind(sub.dmel.df, sub.arab.df)
age.evalue$analysis <- rep("before", nrow(age.evalue))

# getting the distribution of evalues for each clade:
sum.evalue <- ddply(age.evalue, c("species", "Clade"), function(x) {
  sum.dt <- summary(x$evalue)
```

```
  qt1 <- as.numeric(sum.dt[2])
  med <- as.numeric(sum.dt[3])
  mean <- as.numeric(sum.dt[4])
  qt2 <- as.numeric(sum.dt[5])
  data.frame(qt1, med, mean, qt2)
})
sum.evalue <- na.omit(sum.evalue)

# correlation between gene age and evalue:
cor.df <- ddply(sum.evalue, c("species"), function(x) {
  var <- as.numeric(factor(x$Clade))
  med.value <- as.numeric(factor(x$med))
  corr <- cor.test(var, med.value, method = "kendall", exact = FALSE)
  Kendall.tau <- corr$estimate
  p.value <- corr$p.value
  data.frame(Kendall.tau, p.value)
})

kable(cor.df, caption = "Correlation between gene age and E-value")
```

Table 1: Correlation between gene age and E-value

species	Kendall.tau	p.value
Arabidopsis	0.9513920	0.0000012
Drosophila	0.5640761	0.0249059

```
# theme plot
theme.plot <- function(x) {
  theme(axis.title = element_text(face = "bold", color = "black", size=14),
        text = element_text(size=14),
        axis.title.x = element_text(margin = margin(t = 18, r = 10, b = 0, l = 0)),
        axis.title.y = element_text(margin = margin(t = 18, r = 10, b = 0, l = 0)),
        panel.grid.minor=element_blank(),
        panel.grid.major = element_line(colour = "grey", linetype = "dashed", size = 0.2),
        panel.grid.major.y=element_blank(),
        #strip.text.y = element_blank(),
        axis.text.x = element_text(angle = 60, hjust = 1))
}

# box plot:
# Drosophila
sub.dmel.df <- na.omit(sub.dmel.df)
sub.dmel.df$Clade <- factor(na.omit(sub.dmel.df$Clade),
                           labels = c("1", "2", "3", "4", "5", "6", "7", "8", "9",
                                      "10", "11", "12"))
eval.bplot.dmel <- ggplot(sub.dmel.df, aes(Clade, evalue)) +
  #geom_line(col = "black", size = 0.4) +
  geom_boxplot(outlier.size = 0.6) +
  geom_smooth(method = "glm", formula = y~x, se = FALSE) +
  geom_point(size=.2) +
  #ylim(0,1e-10) +
  scale_y_log10() +
```

```
  xlab("Clade") +
  ylab("Blastp evalue") +
  ggtitle("Drosophila") +
  theme_bw() +
  theme.plot()
eval.bplot.dmel
```

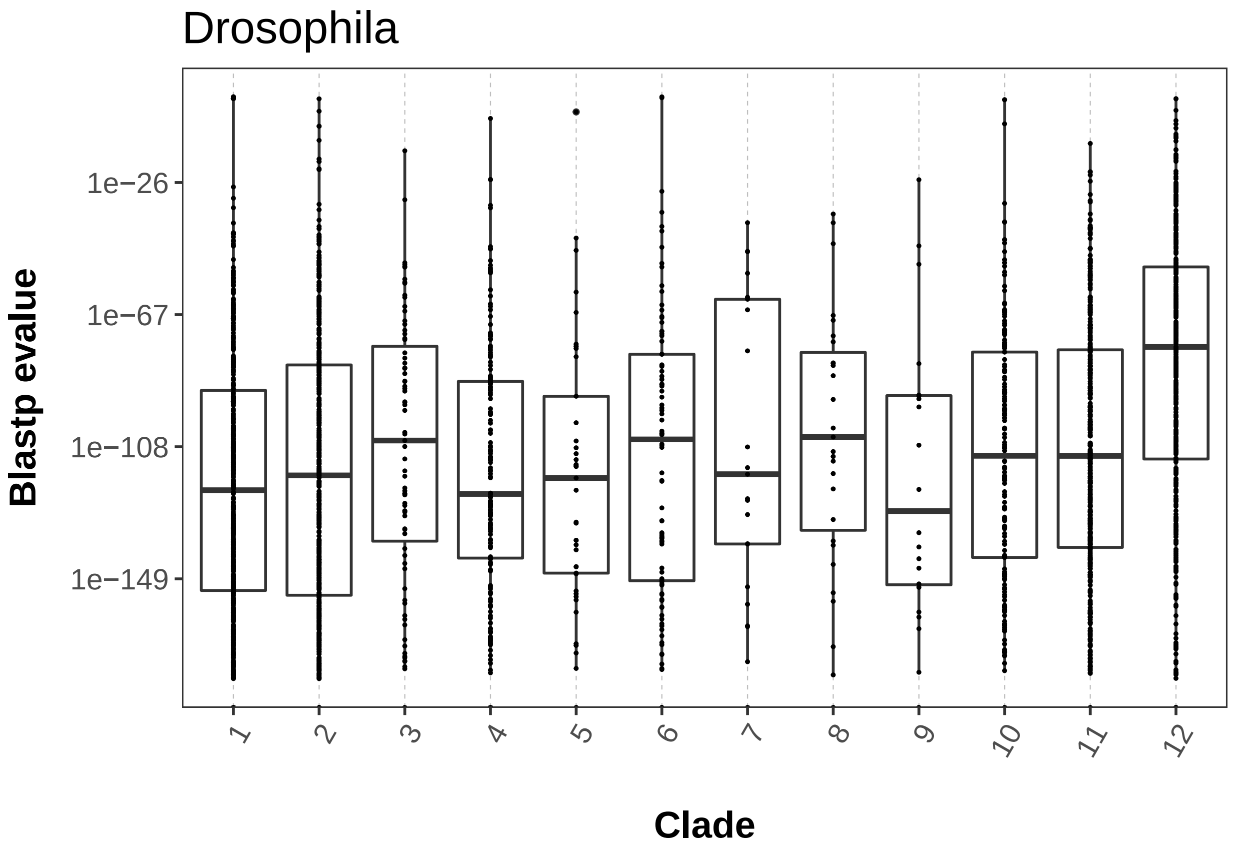


```
# Arabiodpsis
sub.arab.df <- na.omit(sub.arab.df)
sub.arab.df$Clade <- factor(na.omit(sub.arab.df$Clade),
                            labels = c("1", "2", "3", "4", "5", "6", "7", "8", "9", "10", "11",
                                       "12", "13", "14", "15"))
eval.bplot.arab <- ggplot(sub.arab.df, aes(Clade, evalue)) +
  #geom_line(col = "black", size = 0.4) +
  geom_boxplot(outlier.size = 0.6) +
  geom_smooth(method = "glm", formula = y~x, se = FALSE) +
  geom_point(size=.2) +
  #ylim(0,1e-10) +
  scale_y_log10() +
  xlab("Clade") +
  ylab("Blastp evalue") +
  ggtitle("Arabidopsis") +
  theme_bw() +
  theme.plot()
eval.bplot.arab
```

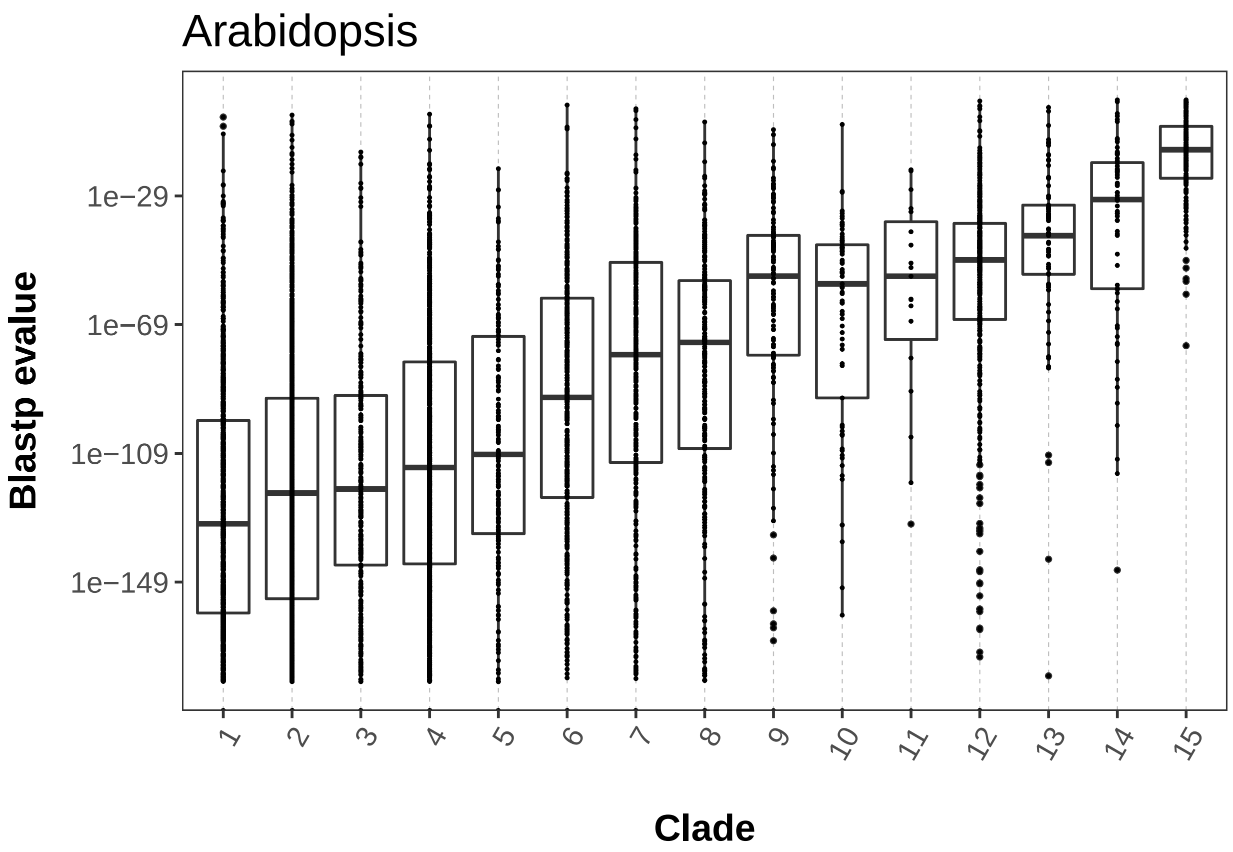


```
bplot.evalue <- plot_grid(eval.bplot.dmel, eval.bplot.arab, nrow = 2)
```

Subsetting the data to keep only genes with the lowest E-values in Drosophila species:

```
# evalue of 1e-100:
e100.dmel <- subset(sub.dmel.df, evalue < 1e-100)

# checking whether the correlation persists after filtering the data:
e100.dmel$Clade <- factor(na.omit(e100.dmel$Clade),
                             labels = c("1", "2", "3", "4", "5", "6", "7", "8", "9", "10", "11", "12")
bplot.dmel.e100 <- ggplot(e100.dmel, aes(Clade, evalue)) +
  #geom_line(col = "black", size = 0.4) +
  geom_boxplot(outlier.size = 0.6) +
  #stat_summary(fun.y=mean, geom="point", shape=20, size=6, color="red", fill="red") +
  geom_point(size=.2) +
  #ylim(0,1e-10) +
  scale_y_log10() +
  xlab("Clade") +
  ylab("") +
  theme_bw() +
  theme.plot()
bplot.dmel.e100
```

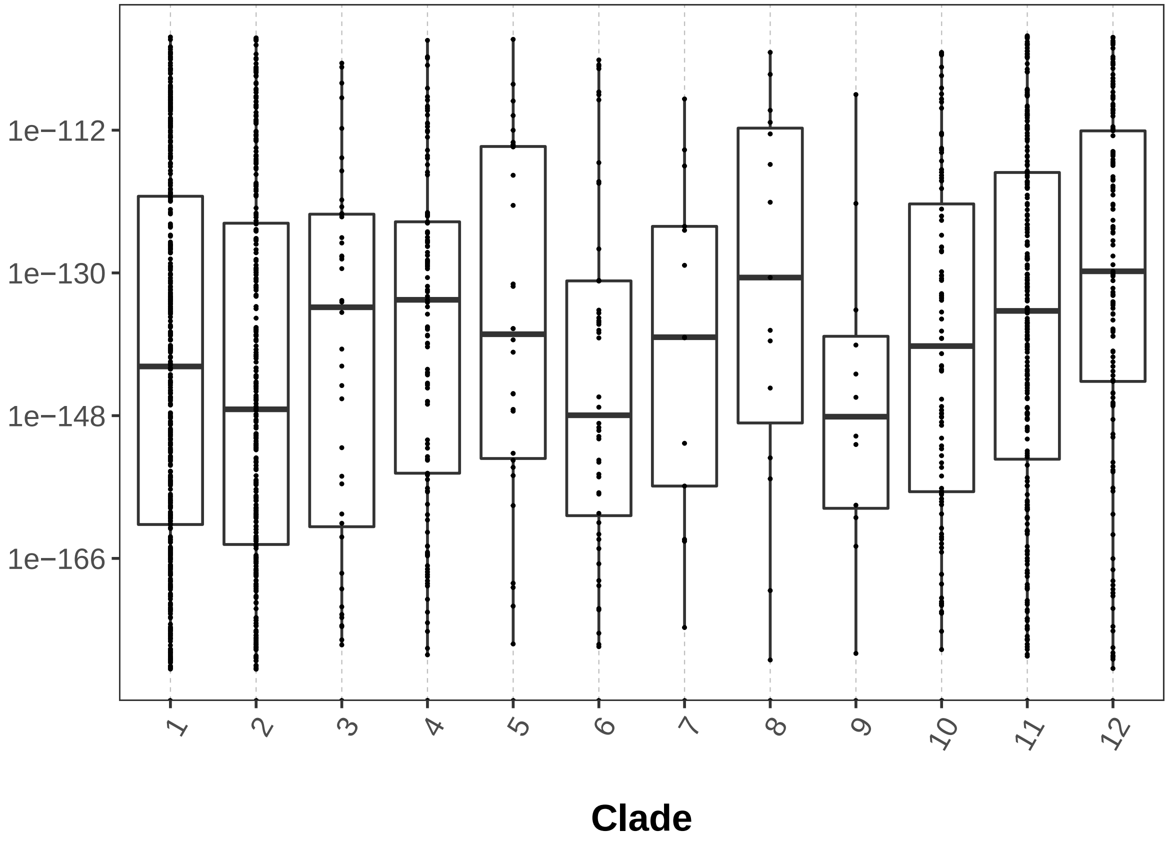


```
# stats:
sum.dmel.e100 <- ddply(e100.dmel, "Clade", function(x) {
  df.sum <- summary(x$evalue)
  median <- as.numeric(df.sum[3])
  mean <- as.numeric(df.sum[4])
  data.frame(median, mean)
})
var.dmel <- as.numeric(factor(sum.dmel.e100$Clade))
mean.value.dmel <- as.numeric(factor(sum.dmel.e100$median))
corr.dmel = cor.test(var.dmel, mean.value.dmel, method = "kendall", exact = FALSE)
Kendall.tau = corr.dmel$estimate
p.value = corr.dmel$p.value
cor.eval.dmel = data.frame(Kendall.tau, p.value)

kable(cor.eval.dmel, caption = "Correlation between gene age and E-value after
      subsetting the data in Drosophila")
```

Table 2: Correlation between gene age and E-value after subsetting the data in Drosophila

	Kendall.tau	p.value
tau	0.4082483	0.111103

Subsetting the data to keep only genes with the lowest E-values in Arabidopsis species:

```r
# evalue of 1e-150:
e150.arab <- subset(sub.arab.df, evalue < 1e-150)

# checking whether the correlation persists after filtering the data:
e150.arab$Clade <- factor(na.omit(e150.arab$Clade),
                           labels = c("1", "2", "3", "4", "5", "6", "7", "8", "9", "10", "11", "12",
bplot.arab.e150 <- ggplot(e150.arab, aes(Clade, evalue)) +
  #geom_line(col = "black", size = 0.4) +
  geom_boxplot(outlier.size = 0.6) +
  #stat_summary(fun.y=mean, geom="point", shape=20, size=6, color="red", fill="red") +
  geom_point(size=.2) +
  #ylim(0,1e-10) +
  scale_y_log10() +
  xlab("Clade") +
  ylab("") +
  theme_bw() +
  theme.plot()
bplot.arab.e150
```

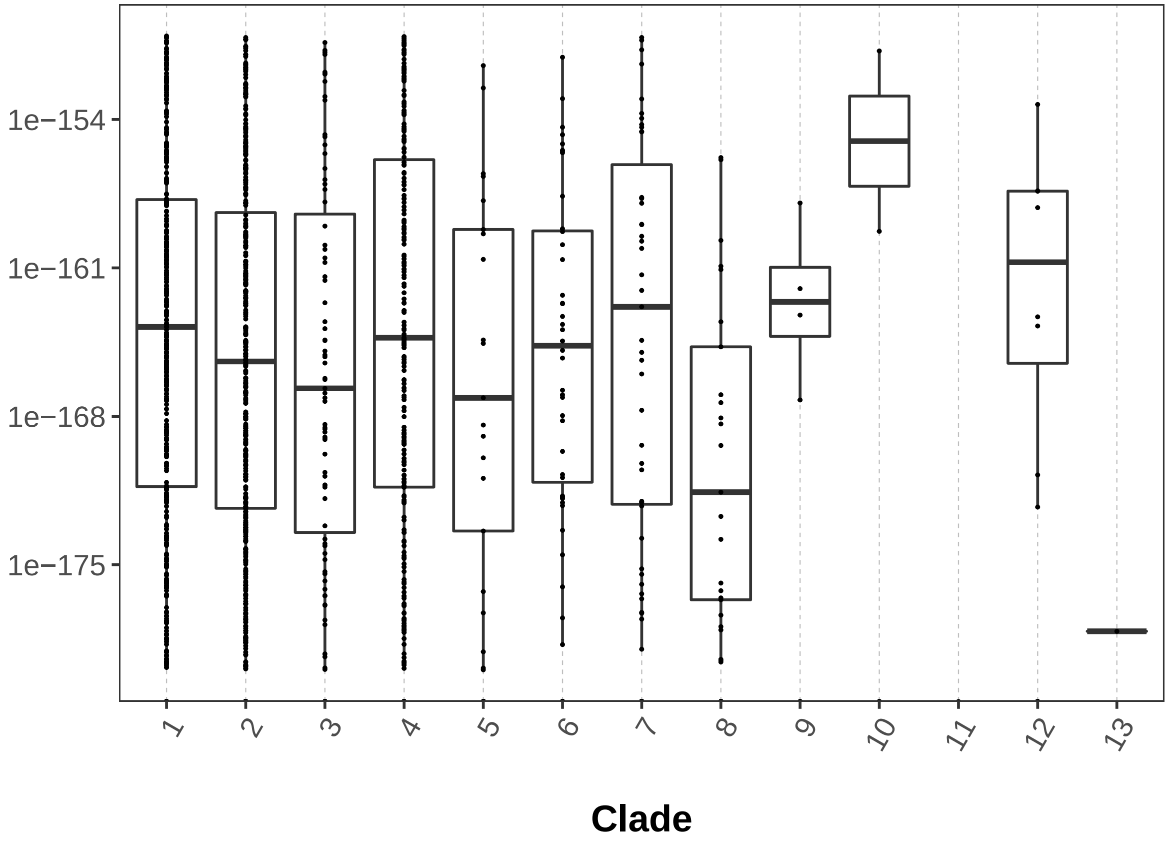


```r
# stats:
sum.arab.e150 <- ddply(e150.arab, "Clade", function(x) {
  df.sum <- summary(x$evalue)
  median <- as.numeric(df.sum[3])
  mean <- as.numeric(df.sum[4])
  data.frame(median, mean)
```

```
})
var.arab <- as.numeric(factor(sum.arab.e150$Clade))
mean.value.arab <- as.numeric(factor(sum.arab.e150$median))
corr.arab = cor.test(var.arab, mean.value.arab, method = "kendall", exact = FALSE)
Kendall.tau = corr.arab$estimate
p.value = corr.arab$p.value
cor.eval.arab = data.frame(Kendall.tau, p.value)

kable(cor.eval.arab, caption = "Correlation between gene age and E-value after
      subsetting the data in Arabidopsis")
```

Table 3: Correlation between gene age and E-value after subsetting the data in Arabidopsis

	Kendall.tau	p.value
tau	0.3541441	0.140688

```
# combining the "before" and "after" tables:
after_evalue <- rbind(e100.dmel, e150.arab)
after_evalue$analysis <- rep("after", nrow(after_evalue))
evalue_age <- rbind(age.evalue, after_evalue)
# saving the S20 Data table to reproduce Figure S3.
write.table(evalue_age, file = "~/Dropbox/SupplementaryData_GeneAge/Evalue/S23_Data.csv",
            sep = "\t", col.names = T, row.names = F, quote = F)
```

Grapes was run on the subset of the data and the results can be seen in the next chunk of the script:

```
# reading the table with the results:
age_evalue_boots <- read.table(file = "~/Dropbox/SupplementaryData_GeneAge/Evalue/S24_Data.csv",
                               sep = "\t",
                               header = TRUE)

# arranging the table for plotting:
age_evalue_boots2 <- melt(age_evalue_boots, id.vars = c("GeneAge", "species"),
       measure.vars = c("dnds", "omegaNA", "omegaA"))

# function to estimate the mean and standard deviation to plot the results with the
# mean of the bootstrap replicates and the 95% confidence interval
fun <- function(x){
  c(mean=mean(x), sd=sd(x))
}

# applying the above function
tbl.sum <- summaryBy(value ~ variable + GeneAge + species,
                     data=age_evalue_boots2, FUN = fun)

# Plotting:
tbl.sum$variable <- factor(tbl.sum$variable, levels = c("dnds", "omegaNA", "omegaA"))
levels(tbl.sum$variable) <- c(expression(omega), expression(omega[na]),
                                     expression(omega[a]))
```

```r
# theme of the plot
theme.plot <- function(x) {
  theme(axis.title = element_text(face = "bold", color = "black", size=14),
        text = element_text(size=14),
        axis.title.x = element_text(margin = margin(t = 18, r = 10, b = 0, l = 0)),
        axis.title.y = element_text(margin = margin(t = 18, r = 10, b = 0, l = 0)),
        panel.grid.minor=element_blank(),
        panel.grid.major = element_line(colour = "grey", linetype = "dashed", size = 0.2),
        panel.grid.major.y=element_blank(),
        #strip.text.y = element_blank(),
        axis.text.x = element_text(angle = 60, hjust = 1),
        legend.position = "none")
}

# plotting each of the output tables
plot.evalue <- ggplot(tbl.sum, aes(x = GeneAge, y = value.mean)) +
  geom_line(col = "black", size = 0.2) +
  geom_ribbon(aes(ymin=value.mean + 1.96*value.sd,
                  ymax=value.mean - 1.96*value.sd, alpha=0.6)) +
  geom_point(size=.2)+
  facet_grid(species~variable, scales = "free_x", labeller = label_parsed) +
  ylab("") +
  xlab("Gene Age") +
  scale_fill_grey() +
  scale_color_grey() +
  theme_bw() +
  theme.plot()
plot.evalue
```

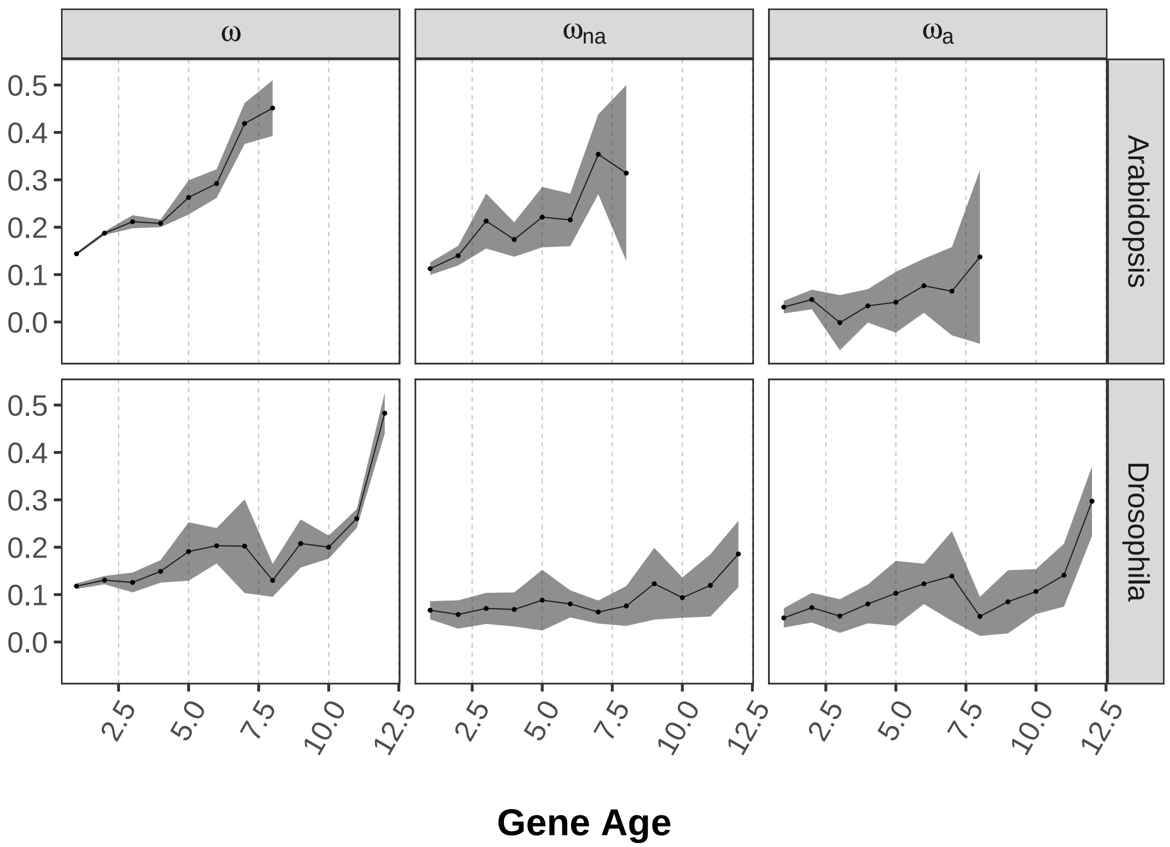

Statistical analyses:

```r
tbl.stat <- ddply(tbl.sum, c("species", "variable"), function(x) {
    var <- as.numeric(factor(x$GeneAge))
    variable.value <- as.numeric(factor(x$value.mean))
    corr <- cor.test(var, variable.value, method = "kendall", exact = FALSE)
    Kendall.tau <- corr$estimate
    p.value <- corr$p.value
    data.frame(Kendall.tau, p.value)
    })

# showing the tables
kable(tbl.stat, caption = "Statistics for the analyses of gene age
      correcting for E-values")
```

Table 4: Statistics for the analyses of gene age correcting for E-values

species	variable	Kendall.tau	p.value
Arabidopsis	omega	0.9285714	0.0012969
Arabidopsis	omega[na]	0.7857143	0.0064929
Arabidopsis	omega[a]	0.6428571	0.0259525
Drosophila	omega	0.6969697	0.0016086
Drosophila	omega[na]	0.6363636	0.0039762

species	variable	Kendall.tau	p.value
Drosophila	omega[a]	0.6363636	0.0039762
