# Supplementary material for: Strong evidence for the adaptive walk model of gene evolution in Drosophila and Arabidopsis
Source: PLoS Biol. 2022 Sep 13;20(9):e3001775. doi: 10.1371/journal.pbio.3001775 (PMC9470001; doi:10.1371/journal.pbio.3001775)

function_age

af_moutinho

15/04/2021

The first part of the script reproduces Figure S5, where the functions of young genes are presented.

```
# Libraries
library(plyr)
library(dplyr)
library(data.table)
library(ggplot2)
library(reshape2)
library(doBy)
library(knitr)
library(kableExtra)
#

# theme of the plot
theme.plot <- function(x) {
  theme(axis.title = element_text(face = "bold", color = "black", size=14),
        text = element_text(size=9),
        axis.title.x = element_text(margin = margin(t = 18, r = 10, b = 0, l = 0)),
        axis.title.y = element_text(margin = margin(t = 18, r = 10, b = 0, l = 0)),
        panel.grid.minor=element_blank(),
        panel.grid.major = element_line(colour = "grey", linetype = "dashed", size = 0.2),
        panel.grid.major.y=element_blank(),
        #strip.text.y = element_blank(),
        axis.text.x = element_text(angle = 60, hjust = 1))
}

# reproducing figure S5
age_function <- read.table(file = "~/Dropbox/SupplementaryData_GeneAge/Function/S25_Data.csv",
                           sep = "\t", header = T)
# change path to where you keep the data

# Drosophila
dmel_go <- subset(age_function, species == "Drosophila")

young_dmel <- na.omit(dmel_go[dmel_go$Clade == 11 | dmel_go$Clade == 12,])

GO_term <- as.character(names(table(young_dmel$GO_slim)))
ngenes <- as.numeric(table(young_dmel$GO_slim))
young_GO_dmel <- data.frame(GO_term, ngenes)

# plotting the functions of young genes:
# keep only GO terms with young genes
```

```
young_GO_dmel <- young_GO_dmel[young_GO_dmel$ngenes > 0,]
# order according to number of genes
young_GO_dmel <- young_GO_dmel[order(young_GO_dmel$ngenes, decreasing = FALSE),]


p.dmel.young <- ggplot(young_GO_dmel, aes(GO_term)) +
  geom_bar(aes(GO_term, ngenes), position = "dodge", stat = "identity", width = 0.7) +
  ylab("Number of genes") +
  xlab("Protein Functional Classification") +
  scale_x_discrete(limits = as.character(young_GO_dmel$GO_term), expand=c(-1, 4)) +
  theme_bw() +
  theme.plot() +
  coord_flip()
p.dmel.young
```

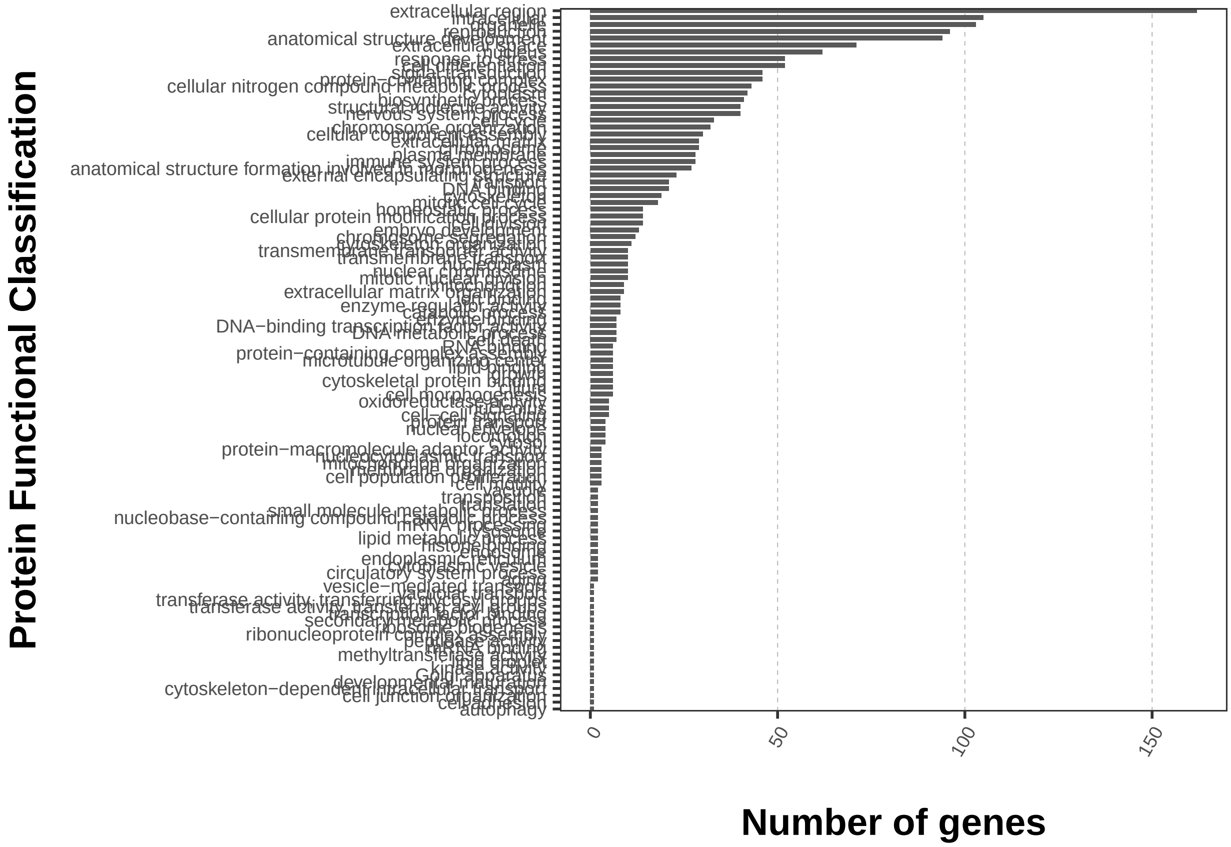


```
# Arabidopsis
arab_go <- subset(age_function, species == "Arabidopsis")

young_arab <- na.omit(arab_go[arab_go$Clade == 12 | arab_go$Clade == 13 |
                               arab_go$Clade == 14 | arab_go$Clade == 15,])

GO_term <- as.character(names(table(young_arab$GO_slim)))
ngenes <- as.numeric(table(young_arab$GO_slim))
young_GO_arab <- data.frame(GO_term, ngenes)
```

```r
# plotting the functions of young genes:
# keep only GO terms with young genes
young_GO_arab <- young_GO_arab[young_GO_arab$ngenes > 0,]
# order according to number of genes
young_GO_arab <- young_GO_arab[order(young_GO_arab$ngenes, decreasing = FALSE),]


p.arab.young <- ggplot(young_GO_arab, aes(GO_term)) +
  geom_bar(aes(GO_term, ngenes), position = "dodge", stat = "identity") +
  ylab("Number of genes") +
  xlab("Protein Functional Classification") +
  scale_x_discrete(limits = as.character(young_GO_arab$GO_term)) +
  theme_bw() +
  theme.plot() +
  coord_flip()
p.arab.young
```

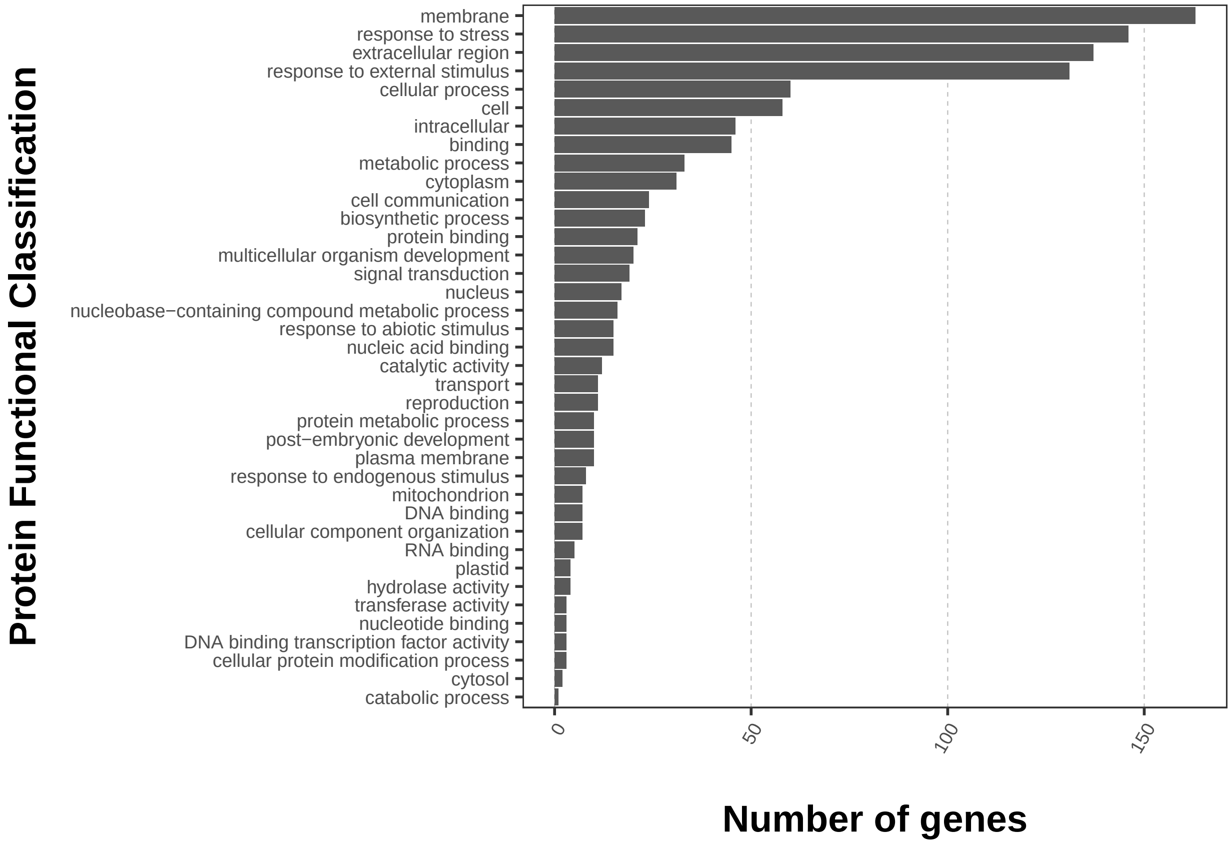

This script includes the data analysis of the gene age data together with protein function.

```r
setwd("~/Dropbox/SupplementaryData_GeneAge/Function/")
# change path to where you keep the data

tbl.function <- read.table(file = "~/Dropbox/SupplementaryData_GeneAge/Function/S26_Data.csv",
                           sep = "\t", header = TRUE)

# arranging the table for plotting (will only plot omega A and omega NA)
```

```r
tbl.function2 <- melt(tbl.function, id.vars = c("GeneAge", "category", "co_factor", "species"),
       measure.vars = c("omegaNA", "omegaA"))

# function to estimate the mean and standard deviation to plot the results with the
# mean of the bootstrap replicates and the 95% confidence interval
fun <- function(x){
  c(mean=mean(x), sd=sd(x))
}

# applying the above function to each output table for each value of each estimate
# (dnds, omegaA, omegaNA) for each value of the variable being analyzed for each species
tbl.sum <- summaryBy(value ~ variable + GeneAge + category + species + co_factor,
                     data=tbl.function2, FUN = fun)

# variables as factors:
tbl.sum$variable <- factor(tbl.sum$variable, levels = c("omegaNA", "omegaA"))
levels(tbl.sum$variable) <- c(expression(omega[na]),expression(omega[a]))

tbl.sum$GeneAge <- as.character(tbl.sum$GeneAge)
```

Including Plots

In the next chunk the script to plot the results is represented. The same order will follow.

```r
# theme of the plot
theme.plot <- function(x) {
  theme(axis.title = element_text(face = "bold", color = "black", size=14),
        text = element_text(size=14),
        axis.title.x = element_text(margin = margin(t = 18, r = 10, b = 0, l = 0)),
        axis.title.y = element_text(margin = margin(t = 18, r = 10, b = 0, l = 0)),
        panel.grid.minor=element_blank(),
        panel.grid.major = element_line(colour = "grey", linetype = "dashed", size = 0.2),
        panel.grid.major.y=element_blank(),
        #strip.text.y = element_blank(),
        axis.text.x = element_text(angle = 60, hjust = 1))
}


#### DROSOPHILA
dmel.go.age <- subset(tbl.sum, species == "Drosophila")

# checking which fucntions have the highest values for omegaA
mean.vars.dmel <- ddply(dmel.go.age, c("category", "variable"), function(x) {
  mean.value <- mean(x$value.mean)
  data.frame(mean.value)
})

mean.omegaA <- subset(mean.vars.dmel, variable == "omega[a]")

# ordering the functions from low to high omega A values
mean.omegaA <- mean.omegaA[order(mean.omegaA$mean.value, decreasing = FALSE),]
dmel.go.age$GeneAge <- as.character(dmel.go.age$GeneAge)
```

```
# plot:
p.dmel <- ggplot(dmel.go.age, aes(x = category, y = value.mean, color = GeneAge)) +
  geom_line(col = "black", size = 0.1)+
  geom_errorbar(aes(ymin=value.mean - 1.96*value.sd,
                    ymax=value.mean + 1.96*value.sd, color = GeneAge), size = 0.6,
                alpha=0.8, width = .2) +
  geom_point(size=.6)+
  facet_wrap(~variable, scales = "free_x", labeller = label_parsed) +
  ylab("") +
  xlab("Protein Function") +
  scale_x_discrete(limits = as.character(mean.omegaA$category)) +
  scale_fill_grey() +
  scale_color_grey() +
  theme_bw() +
  theme.plot() +
  coord_flip()
p.dmel
```

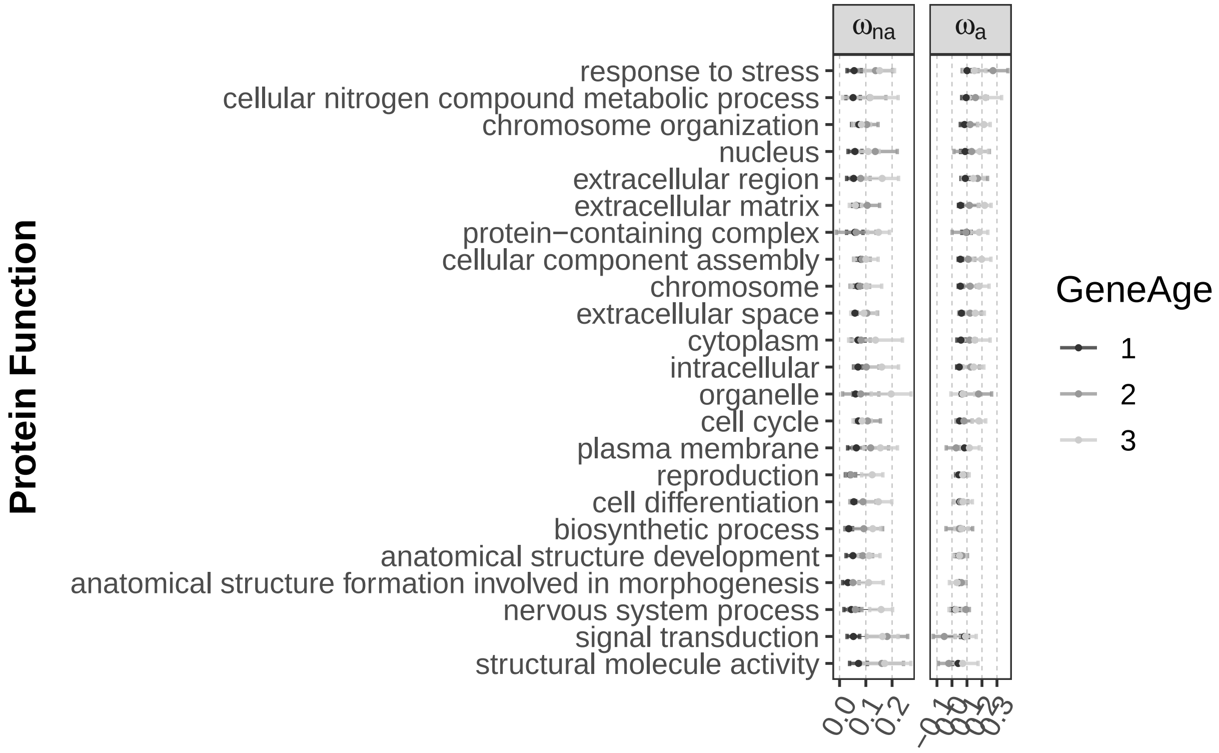


```
#### ARABIDOPSIS
arab.go.age <- subset(tbl.sum, species == "Arabidopsis")

# checking which fucntions have the highest values for omegaA
mean.vars <- ddply(arab.go.age, c("category", "variable"), function(x) {
  mean.value <- mean(x$value.mean)
  data.frame(mean.value)
```

```
})
mean.omegaA <- subset(mean.vars, variable == "omega[a]")

# ordering the functions from low to high omega A values
mean.omegaA <- mean.omegaA[order(mean.omegaA$mean.value, decreasing = FALSE),]
arab.go.age$GeneAge <- as.character(arab.go.age$GeneAge)

# plot:
p.arab <- ggplot(arab.go.age, aes(x = category, y = value.mean, color = GeneAge)) +
  geom_line(col = "black", size = 0.1)+
  geom_errorbar(aes(ymin=value.mean - 1.96*value.sd,
                    ymax=value.mean + 1.96*value.sd, color = GeneAge), size = 0.6,
                alpha=0.8, width = .2) +
  geom_point(size=.6)+
  facet_wrap(~variable, scales = "free_x", labeller = label_parsed) +
  ylab("") +
  xlab("Protein Function") +
  scale_x_discrete(limits = as.character(mean.omegaA$category)) +
  scale_fill_grey() +
  scale_color_grey() +
  theme_bw() +
  theme.plot() +
  coord_flip()
p.arab
```

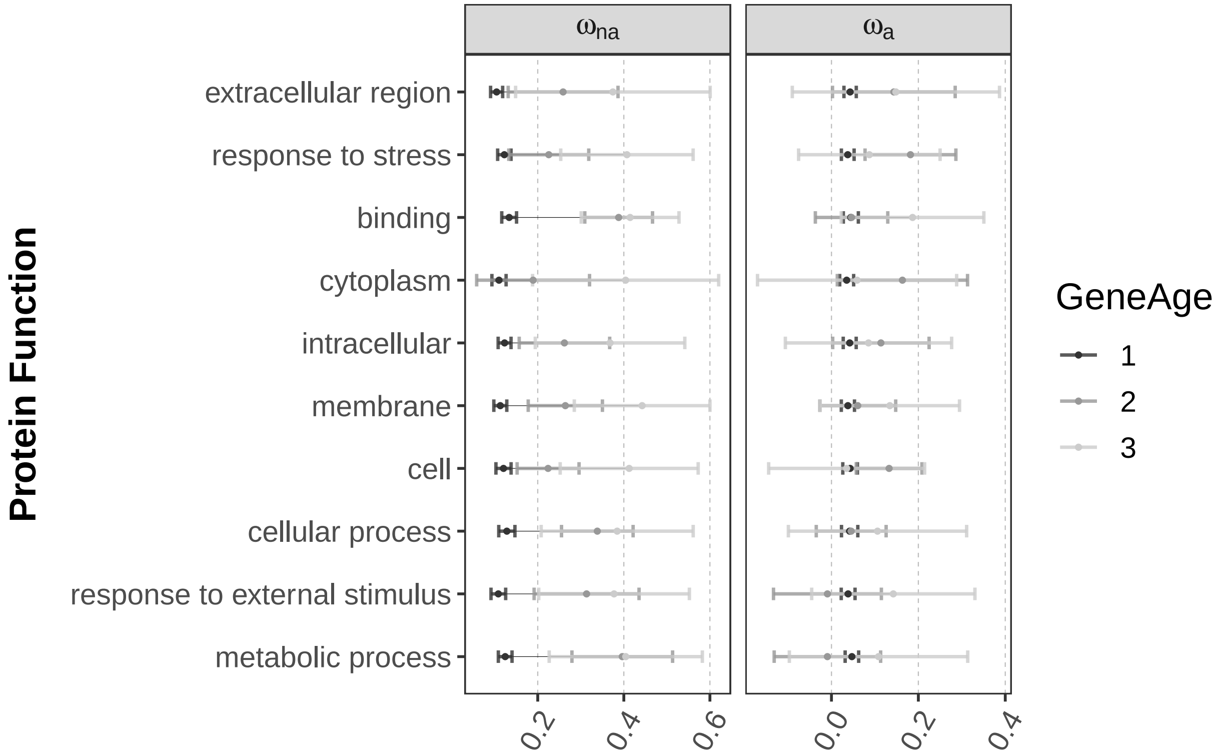

The last section shows how the statistical analyses were performed.

```r
### something is wrong here --> check this
# estimating the differences between age classes for each bootstrap replicate
df.dif <- ddply(tbl.function2, c("species", "category", "co_factor", "variable"),
                   .progress = "text", function(x) {
                     age2_age1 <- x$value[x$GeneAge == 2] - x$value[x$GeneAge == 1]
                     age3_age1 <- x$value[x$GeneAge == 3] - x$value[x$GeneAge == 1]
                     age3_age2 <- x$value[x$GeneAge == 3] - x$value[x$GeneAge == 2]
                     data.frame(age2_age1, age3_age1, age3_age2)
                     })
```

```
##   |                                                                              |
```

```r
colnames(df.dif)[4] <- "estimate"

go.dif.age <- reshape2::melt(df.dif, id.vars = c("species", "category", "co_factor",
                                                  "estimate"),
                                            measure.vars = c("age2_age1", "age3_age1",
                                                             "age3_age2"))

# estimating the p-value between the different categories of age:
# Since we are sampling across bootstrap replicates, the p-values will be estimated 100 times and
# then the minimum p-value will be used
stats.dif.age <- ddply(go.dif.age, c("species", "category", "co_factor", "estimate",
                                     "variable"),
                      function(x, N = 100) {
                         c <- as.numeric(nrow(x[x$value < 0,]))
                         c2 <- as.numeric(nrow(x[x$value > 0,]))
                         m <- min(c, c2)
                         p <- (2*m+1)/(N+1)
                         data.frame(c, c2, m, p)
                         })

# correcting for multiple testing:
# now applying p-adjust:
padjust.stats <- ddply(stats.dif.age, c("species", "category", "co_factor",
                                        "estimate"),
        .progress = "text", function(x) {
          x$p.value.adj <- p.adjust(x$p, method = "fdr")
          return(x)
          })
```

```
##   |                                                                              |
```

```r
kable(padjust.stats, caption = "Statistics for the combined analysis with protein function",
      format = "latex", booktabs = TRUE, longtable = TRUE) %>%
  kable_styling(font_size = 8, latex_options="striped") %>%
  column_spec(2, width = "15em")
```

Table 1: Statistics for the combined analysis with protein function

species	category	co_factor	estimate	variable	c	c2	m	p	p.value.adj

Arabidopsis	binding	Function	omegaNA	age2__age1	0	100	0	0.0099010	0.0148515
Arabidopsis	binding	Function	omegaNA	age3__age1	0	100	0	0.0099010	0.0148515
Arabidopsis	binding	Function	omegaNA	age3__age2	35	65	35	0.7029703	0.7029703
Arabidopsis	binding	Function	omegaA	age2__age1	50	50	50	1.0000000	1.0000000
Arabidopsis	binding	Function	omegaA	age3__age1	6	94	6	0.1287129	0.2821782
Arabidopsis	binding	Function	omegaA	age3__age2	9	91	9	0.1881188	0.2821782
Arabidopsis	cell	Function	omegaNA	age2__age1	0	100	0	0.0099010	0.0148515
Arabidopsis	cell	Function	omegaNA	age3__age1	0	100	0	0.0099010	0.0148515
Arabidopsis	cell	Function	omegaNA	age3__age2	2	98	2	0.0495050	0.0495050
Arabidopsis	cell	Function	omegaA	age2__age1	3	97	3	0.0693069	0.2079208
Arabidopsis	cell	Function	omegaA	age3__age1	57	43	43	0.8613861	0.8613861
Arabidopsis	cell	Function	omegaA	age3__age2	84	16	16	0.3267327	0.4900990
Arabidopsis	cellular process	Function	omegaNA	age2__age1	0	100	0	0.0099010	0.0297030
Arabidopsis	cellular process	Function	omegaNA	age3__age1	2	98	2	0.0495050	0.0742574
Arabidopsis	cellular process	Function	omegaNA	age3__age2	28	72	28	0.5643564	0.5643564
Arabidopsis	cellular process	Function	omegaA	age2__age1	43	57	43	0.8613861	0.8613861
Arabidopsis	cellular process	Function	omegaA	age3__age1	25	75	25	0.5049505	0.8613861
Arabidopsis	cellular process	Function	omegaA	age3__age2	29	71	29	0.5841584	0.8613861
Arabidopsis	cytoplasm	Function	omegaNA	age2__age1	11	89	11	0.2277228	0.2277228
Arabidopsis	cytoplasm	Function	omegaNA	age3__age1	1	99	1	0.0297030	0.0891089
Arabidopsis	cytoplasm	Function	omegaNA	age3__age2	6	94	6	0.1287129	0.1930693
Arabidopsis	cytoplasm	Function	omegaA	age2__age1	5	95	5	0.1089109	0.3267327
Arabidopsis	cytoplasm	Function	omegaA	age3__age1	45	55	45	0.9009901	0.9009901
Arabidopsis	cytoplasm	Function	omegaA	age3__age2	78	22	22	0.4455446	0.6683168
Arabidopsis	extracellular region	Function	omegaNA	age2__age1	1	99	1	0.0297030	0.0445545
Arabidopsis	extracellular region	Function	omegaNA	age3__age1	1	99	1	0.0297030	0.0445545
Arabidopsis	extracellular region	Function	omegaNA	age3__age2	19	81	19	0.3861386	0.3861386
Arabidopsis	extracellular region	Function	omegaA	age2__age1	9	91	9	0.1881188	0.5643564
Arabidopsis	extracellular region	Function	omegaA	age3__age1	19	81	19	0.3861386	0.5792079
Arabidopsis	extracellular region	Function	omegaA	age3__age2	58	42	42	0.8415842	0.8415842
Arabidopsis	intracellular	Function	omegaNA	age2__age1	1	99	1	0.0297030	0.0445545
Arabidopsis	intracellular	Function	omegaNA	age3__age1	0	100	0	0.0099010	0.0297030
Arabidopsis	intracellular	Function	omegaNA	age3__age2	11	89	11	0.2277228	0.2277228
Arabidopsis	intracellular	Function	omegaA	age2__age1	12	88	12	0.2475248	0.7425743
Arabidopsis	intracellular	Function	omegaA	age3__age1	34	66	34	0.6831683	0.7425743
Arabidopsis	intracellular	Function	omegaA	age3__age2	62	37	37	0.7425743	0.7425743
Arabidopsis	membrane	Function	omegaNA	age2__age1	0	100	0	0.0099010	0.0148515
Arabidopsis	membrane	Function	omegaNA	age3__age1	0	100	0	0.0099010	0.0148515
Arabidopsis	membrane	Function	omegaNA	age3__age2	4	96	4	0.0891089	0.0891089
Arabidopsis	membrane	Function	omegaA	age2__age1	34	66	34	0.6831683	0.6831683
Arabidopsis	membrane	Function	omegaA	age3__age1	15	85	15	0.3069307	0.6831683
Arabidopsis	membrane	Function	omegaA	age3__age2	24	76	24	0.4851485	0.6831683
Arabidopsis	metabolic process	Function	omegaNA	age2__age1	0	100	0	0.0099010	0.0297030
Arabidopsis	metabolic process	Function	omegaNA	age3__age1	2	98	2	0.0495050	0.0742574
Arabidopsis	metabolic process	Function	omegaNA	age3__age2	47	53	47	0.9405941	0.9405941
Arabidopsis	metabolic process	Function	omegaA	age2__age1	86	14	14	0.2871287	0.4306931
Arabidopsis	metabolic process	Function	omegaA	age3__age1	29	71	29	0.5841584	0.5841584
Arabidopsis	metabolic process	Function	omegaA	age3__age2	14	86	14	0.2871287	0.4306931
Arabidopsis	response to external stimulus	Function	omegaNA	age2__age1	1	99	1	0.0297030	0.0445545
Arabidopsis	response to external stimulus	Function	omegaNA	age3__age1	1	99	1	0.0297030	0.0445545
Arabidopsis	response to external stimulus	Function	omegaNA	age3__age2	26	74	26	0.5247525	0.5247525
Arabidopsis	response to external stimulus	Function	omegaA	age2__age1	82	18	18	0.3663366	0.3663366
Arabidopsis	response to external stimulus	Function	omegaA	age3__age1	12	88	12	0.2475248	0.3663366
Arabidopsis	response to external stimulus	Function	omegaA	age3__age2	10	90	10	0.2079208	0.3663366
Arabidopsis	response to stress	Function	omegaNA	age2__age1	1	99	1	0.0297030	0.0297030
Arabidopsis	response to stress	Function	omegaNA	age3__age1	0	100	0	0.0099010	0.0297030
Arabidopsis	response to stress	Function	omegaNA	age3__age2	1	99	1	0.0297030	0.0297030
Arabidopsis	response to stress	Function	omegaA	age2__age1	1	99	1	0.0297030	0.0891089
Arabidopsis	response to stress	Function	omegaA	age3__age1	28	72	28	0.5643564	0.5643564

Arabidopsis	response to stress	Function	omegaA	age3__age2	83	17	17	0.3465347	0.5198020
Drosophila	anatomical structure development	Function	omegaNA	age2__age1	3	97	3	0.0693069	0.1039604
Drosophila	anatomical structure development	Function	omegaNA	age3__age1	0	100	0	0.0099010	0.0297030
Drosophila	anatomical structure development	Function	omegaNA	age3__age2	25	75	25	0.5049505	0.5049505
Drosophila	anatomical structure development	Function	omegaA	age2__age1	24	76	24	0.4851485	0.9009901
Drosophila	anatomical structure development	Function	omegaA	age3__age1	45	54	45	0.9009901	0.9009901
Drosophila	anatomical structure development	Function	omegaA	age3__age2	66	34	34	0.6831683	0.9009901
Drosophila	anatomical structure formation involved in morphogenesis	Function	omegaNA	age2__age1	7	93	7	0.1485149	0.1485149
Drosophila	anatomical structure formation involved in morphogenesis	Function	omegaNA	age3__age1	1	99	1	0.0297030	0.0742574
Drosophila	anatomical structure formation involved in morphogenesis	Function	omegaNA	age3__age2	2	98	2	0.0495050	0.0742574
Drosophila	anatomical structure formation involved in morphogenesis	Function	omegaA	age2__age1	25	75	25	0.5049505	0.5445545
Drosophila	anatomical structure formation involved in morphogenesis	Function	omegaA	age3__age1	73	27	27	0.5445545	0.5445545
Drosophila	anatomical structure formation involved in morphogenesis	Function	omegaA	age3__age2	81	18	18	0.3663366	0.5445545
Drosophila	biosynthetic process	Function	omegaNA	age2__age1	2	98	2	0.0495050	0.0742574
Drosophila	biosynthetic process	Function	omegaNA	age3__age1	0	100	0	0.0099010	0.0297030
Drosophila	biosynthetic process	Function	omegaNA	age3__age2	21	79	21	0.4257426	0.4257426
Drosophila	biosynthetic process	Function	omegaA	age2__age1	57	43	43	0.8613861	0.8613861
Drosophila	biosynthetic process	Function	omegaA	age3__age1	35	65	35	0.7029703	0.8613861
Drosophila	biosynthetic process	Function	omegaA	age3__age2	36	64	36	0.7227723	0.8613861
Drosophila	cell cycle	Function	omegaNA	age2__age1	19	81	19	0.3861386	0.4851485
Drosophila	cell cycle	Function	omegaNA	age3__age1	21	79	21	0.4257426	0.4851485
Drosophila	cell cycle	Function	omegaNA	age3__age2	76	24	24	0.4851485	0.4851485
Drosophila	cell cycle	Function	omegaA	age2__age1	10	90	10	0.2079208	0.2079208
Drosophila	cell cycle	Function	omegaA	age3__age1	0	100	0	0.0099010	0.0297030
Drosophila	cell cycle	Function	omegaA	age3__age2	1	99	1	0.0297030	0.0445545
Drosophila	cell differentiation	Function	omegaNA	age2__age1	11	89	11	0.2277228	0.2277228
Drosophila	cell differentiation	Function	omegaNA	age3__age1	0	100	0	0.0099010	0.0297030
Drosophila	cell differentiation	Function	omegaNA	age3__age2	7	93	7	0.1485149	0.2227723
Drosophila	cell differentiation	Function	omegaA	age2__age1	46	54	46	0.9207921	0.9207921
Drosophila	cell differentiation	Function	omegaA	age3__age1	21	79	21	0.4257426	0.9207921
Drosophila	cell differentiation	Function	omegaA	age3__age2	33	67	33	0.6633663	0.9207921
Drosophila	cellular component assembly	Function	omegaNA	age2__age1	41	59	41	0.8217822	0.8217822
Drosophila	cellular component assembly	Function	omegaNA	age3__age1	14	86	14	0.2871287	0.8217822
Drosophila	cellular component assembly	Function	omegaNA	age3__age2	35	65	35	0.7029703	0.8217822
Drosophila	cellular component assembly	Function	omegaA	age2__age1	2	98	2	0.0495050	0.0693069
Drosophila	cellular component assembly	Function	omegaA	age3__age1	0	100	0	0.0099010	0.0297030
Drosophila	cellular component assembly	Function	omegaA	age3__age2	3	97	3	0.0693069	0.0693069
Drosophila	cellular nitrogen compound metabolic process	Function	omegaNA	age2__age1	0	100	0	0.0099010	0.0297030
Drosophila	cellular nitrogen compound metabolic process	Function	omegaNA	age3__age1	13	87	13	0.2673267	0.4009901
Drosophila	cellular nitrogen compound metabolic process	Function	omegaNA	age3__age2	50	50	50	1.0000000	1.0000000
Drosophila	cellular nitrogen compound metabolic process	Function	omegaA	age2__age1	9	91	9	0.1881188	0.2821782
Drosophila	cellular nitrogen compound metabolic process	Function	omegaA	age3__age1	1	99	1	0.0297030	0.0891089
Drosophila	cellular nitrogen compound metabolic process	Function	omegaA	age3__age2	18	82	18	0.3663366	0.3663366
Drosophila	chromosome	Function	omegaNA	age2__age1	46	54	46	0.9207921	0.9207921
Drosophila	chromosome	Function	omegaNA	age3__age1	5	95	5	0.1089109	0.3267327
Drosophila	chromosome	Function	omegaNA	age3__age2	25	75	25	0.5049505	0.7574257
Drosophila	chromosome	Function	omegaA	age2__age1	0	100	0	0.0099010	0.0148515
Drosophila	chromosome	Function	omegaA	age3__age1	0	100	0	0.0099010	0.0148515

Drosophila	chromosome	Function	omegaA	age3__age2	7	93	7	0.1485149	0.1485149
Drosophila	chromosome organization	Function	omegaNA	age2__age1	14	86	14	0.2871287	0.5495050
Drosophila	chromosome organization	Function	omegaNA	age3__age1	32	68	32	0.6435644	0.6435644
Drosophila	chromosome organization	Function	omegaNA	age3__age2	82	18	18	0.3663366	0.5495050
Drosophila	chromosome organization	Function	omegaA	age2__age1	10	90	10	0.2079208	0.2079208
Drosophila	chromosome organization	Function	omegaA	age3__age1	0	100	0	0.0099010	0.0297030
Drosophila	chromosome organization	Function	omegaA	age3__age2	1	99	1	0.0297030	0.0445545
Drosophila	cytoplasm	Function	omegaNA	age2__age1	41	59	41	0.8217822	0.8217822
Drosophila	cytoplasm	Function	omegaNA	age3__age1	2	98	2	0.0495050	0.1485149
Drosophila	cytoplasm	Function	omegaNA	age3__age2	14	86	14	0.2871287	0.4306931
Drosophila	cytoplasm	Function	omegaA	age2__age1	2	98	2	0.0495050	0.0742574
Drosophila	cytoplasm	Function	omegaA	age3__age1	2	98	2	0.0495050	0.0742574
Drosophila	cytoplasm	Function	omegaA	age3__age2	30	70	30	0.6039604	0.6039604
Drosophila	extracellular matrix	Function	omegaNA	age2__age1	5	95	5	0.1089109	0.1930693
Drosophila	extracellular matrix	Function	omegaNA	age3__age1	71	29	29	0.5841584	0.5841584
Drosophila	extracellular matrix	Function	omegaNA	age3__age2	94	6	6	0.1287129	0.1930693
Drosophila	extracellular matrix	Function	omegaA	age2__age1	3	97	3	0.0693069	0.0693069
Drosophila	extracellular matrix	Function	omegaA	age3__age1	0	100	0	0.0099010	0.0297030
Drosophila	extracellular matrix	Function	omegaA	age3__age2	2	98	2	0.0495050	0.0693069
Drosophila	extracellular region	Function	omegaNA	age2__age1	10	90	10	0.2079208	0.2079208
Drosophila	extracellular region	Function	omegaNA	age3__age1	0	100	0	0.0099010	0.0297030
Drosophila	extracellular region	Function	omegaNA	age3__age2	2	98	2	0.0495050	0.0742574
Drosophila	extracellular region	Function	omegaA	age2__age1	2	98	2	0.0495050	0.1485149
Drosophila	extracellular region	Function	omegaA	age3__age1	6	94	6	0.1287129	0.1930693
Drosophila	extracellular region	Function	omegaA	age3__age2	72	27	27	0.5445545	0.5445545
Drosophila	extracellular space	Function	omegaNA	age2__age1	1	99	1	0.0297030	0.0891089
Drosophila	extracellular space	Function	omegaNA	age3__age1	5	95	5	0.1089109	0.1633663
Drosophila	extracellular space	Function	omegaNA	age3__age2	68	32	32	0.6435644	0.6435644
Drosophila	extracellular space	Function	omegaA	age2__age1	8	92	8	0.1683168	0.2524752
Drosophila	extracellular space	Function	omegaA	age3__age1	1	99	1	0.0297030	0.0891089
Drosophila	extracellular space	Function	omegaA	age3__age2	22	78	22	0.4455446	0.4455446
Drosophila	intracellular	Function	omegaNA	age2__age1	20	80	20	0.4059406	0.4059406
Drosophila	intracellular	Function	omegaNA	age3__age1	0	100	0	0.0099010	0.0297030
Drosophila	intracellular	Function	omegaNA	age3__age2	6	94	6	0.1287129	0.1930693
Drosophila	intracellular	Function	omegaA	age2__age1	0	100	0	0.0099010	0.0297030
Drosophila	intracellular	Function	omegaA	age3__age1	2	98	2	0.0495050	0.0742574
Drosophila	intracellular	Function	omegaA	age3__age2	29	71	29	0.5841584	0.5841584
Drosophila	nervous system process	Function	omegaNA	age2__age1	15	85	15	0.3069307	0.3069307
Drosophila	nervous system process	Function	omegaNA	age3__age1	0	100	0	0.0099010	0.0148515
Drosophila	nervous system process	Function	omegaNA	age3__age2	0	100	0	0.0099010	0.0148515
Drosophila	nervous system process	Function	omegaA	age2__age1	0	100	0	0.0099010	0.0148515
Drosophila	nervous system process	Function	omegaA	age3__age1	44	56	44	0.8811881	0.8811881
Drosophila	nervous system process	Function	omegaA	age3__age2	100	0	0	0.0099010	0.0148515
Drosophila	nucleus	Function	omegaNA	age2__age1	2	98	2	0.0495050	0.0742574
Drosophila	nucleus	Function	omegaNA	age3__age1	1	99	1	0.0297030	0.0742574
Drosophila	nucleus	Function	omegaNA	age3__age2	69	31	31	0.6237624	0.6237624
Drosophila	nucleus	Function	omegaA	age2__age1	23	77	23	0.4653465	0.4653465
Drosophila	nucleus	Function	omegaA	age3__age1	0	100	0	0.0099010	0.0297030
Drosophila	nucleus	Function	omegaA	age3__age2	17	83	17	0.3465347	0.4653465
Drosophila	organelle	Function	omegaNA	age2__age1	38	62	38	0.7623762	0.7623762
Drosophila	organelle	Function	omegaNA	age3__age1	0	100	0	0.0099010	0.0297030
Drosophila	organelle	Function	omegaNA	age3__age2	2	98	2	0.0495050	0.0742574
Drosophila	organelle	Function	omegaA	age2__age1	2	98	2	0.0495050	0.1485149
Drosophila	organelle	Function	omegaA	age3__age1	49	51	49	0.9801980	0.9801980
Drosophila	organelle	Function	omegaA	age3__age2	95	5	5	0.1089109	0.1633663
Drosophila	plasma membrane	Function	omegaNA	age2__age1	10	90	10	0.2079208	0.3118812
Drosophila	plasma membrane	Function	omegaNA	age3__age1	0	100	0	0.0099010	0.0297030
Drosophila	plasma membrane	Function	omegaNA	age3__age2	29	71	29	0.5841584	0.5841584
Drosophila	plasma membrane	Function	omegaA	age2__age1	89	11	11	0.2277228	0.3415842

Drosophila	plasma membrane	Function	omegaA	age3_age1	20	80	20	0.4059406	0.4059406
Drosophila	plasma membrane	Function	omegaA	age3_age2	1	99	1	0.0297030	0.0891089
Drosophila	protein-containing complex	Function	omegaNA	age2_age1	58	42	42	0.8415842	0.8415842
Drosophila	protein-containing complex	Function	omegaNA	age3_age1	0	100	0	0.0099010	0.0297030
Drosophila	protein-containing complex	Function	omegaNA	age3_age2	3	97	3	0.0693069	0.1039604
Drosophila	protein-containing complex	Function	omegaA	age2_age1	45	55	45	0.9009901	0.9009901
Drosophila	protein-containing complex	Function	omegaA	age3_age1	0	100	0	0.0099010	0.0297030
Drosophila	protein-containing complex	Function	omegaA	age3_age2	7	93	7	0.1485149	0.2227723
Drosophila	reproduction	Function	omegaNA	age2_age1	45	55	45	0.9009901	0.9009901
Drosophila	reproduction	Function	omegaNA	age3_age1	0	100	0	0.0099010	0.0148515
Drosophila	reproduction	Function	omegaNA	age3_age2	0	100	0	0.0099010	0.0148515
Drosophila	reproduction	Function	omegaA	age2_age1	3	97	3	0.0693069	0.2079208
Drosophila	reproduction	Function	omegaA	age3_age1	11	89	11	0.2277228	0.3415842
Drosophila	reproduction	Function	omegaA	age3_age2	60	40	40	0.8019802	0.8019802
Drosophila	response to stress	Function	omegaNA	age2_age1	0	100	0	0.0099010	0.0297030
Drosophila	response to stress	Function	omegaNA	age3_age1	1	99	1	0.0297030	0.0445545
Drosophila	response to stress	Function	omegaNA	age3_age2	26	74	26	0.5247525	0.5247525
Drosophila	response to stress	Function	omegaA	age2_age1	0	100	0	0.0099010	0.0297030
Drosophila	response to stress	Function	omegaA	age3_age1	10	90	10	0.2079208	0.2079208
Drosophila	response to stress	Function	omegaA	age3_age2	97	3	3	0.0693069	0.1039604
Drosophila	signal transduction	Function	omegaNA	age2_age1	0	100	0	0.0099010	0.0148515
Drosophila	signal transduction	Function	omegaNA	age3_age1	0	100	0	0.0099010	0.0148515
Drosophila	signal transduction	Function	omegaNA	age3_age2	65	35	35	0.7029703	0.7029703
Drosophila	signal transduction	Function	omegaA	age2_age1	100	0	0	0.0099010	0.0148515
Drosophila	signal transduction	Function	omegaA	age3_age1	45	55	45	0.9009901	0.9009901
Drosophila	signal transduction	Function	omegaA	age3_age2	0	100	0	0.0099010	0.0148515
Drosophila	structural molecule activity	Function	omegaNA	age2_age1	3	97	3	0.0693069	0.1039604
Drosophila	structural molecule activity	Function	omegaNA	age3_age1	1	99	1	0.0297030	0.0891089
Drosophila	structural molecule activity	Function	omegaNA	age3_age2	46	54	46	0.9207921	0.9207921
Drosophila	structural molecule activity	Function	omegaA	age2_age1	96	4	4	0.0891089	0.2673267
Drosophila	structural molecule activity	Function	omegaA	age3_age1	25	75	25	0.5049505	0.5049505
Drosophila	structural molecule activity	Function	omegaA	age3_age2	10	90	10	0.2079208	0.3118812

checking the functions coded by genes present in clades 6 & 7 (Figure S8):

```
# sub-setting the data to keep only the genes present in clades 6&7
clades_peak <- subset(dmel_go, Clade == 6 | Clade == 7)

# estimating the total number of genes in each function
genes.clades <- ddply(dmel_go, "GO_slim", function(x) {
  n_total <- nrow(x)
  data.frame(n_total)
})

# number of annotated genes in the functions present in clades 6&7:
ngenes_peak <- ddply(clades_peak, "GO_slim", function(x) {
  n <- nrow(x)
  data.frame(n)
})

# combining both tableS:
clades_go <- merge(ngenes_peak, genes.clades, by = c("GO_slim"))

# estimating the frequency of genes in these clades:
```

```r
clades_go$p.genes <- clades_go$n/clades_go$n_total
clades_go <- clades_go[order(clades_go$p.genes, decreasing = FALSE),]

# estimating the summary:
s.clades <- summary(clades_go$p.genes)

# plotting only those functions for which the frequency is higher than the median value:
sub.clades <- clades_go[clades_go$p.genes > as.numeric(s.clades[3]),]

# plotting
p.clades <- ggplot(sub.clades, aes(GO_slim)) +
  geom_bar(aes(GO_slim, p.genes), position = "dodge", stat = "identity") +
  ylab("Frequency of genes") +
  xlab("Protein Functional Classification") +
  scale_x_discrete(limits = as.character(sub.clades$GO_slim)) +
  theme_bw() +
  theme.plot() +
  coord_flip()
p.clades
```

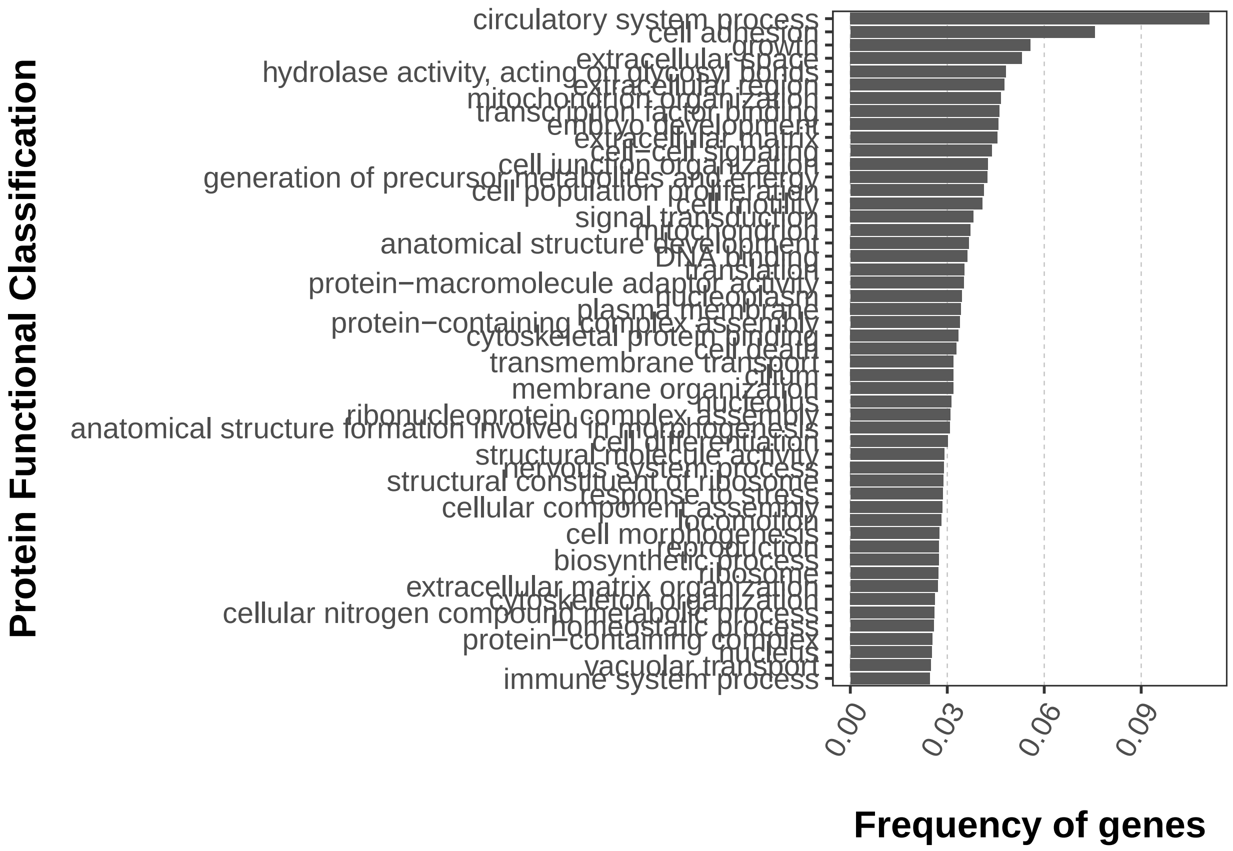

Supplement: S3 File — (PDF) [file pbio.3001775.s049.pdf]
