# Supplementary material for: Strong evidence for the adaptive walk model of gene evolution in Drosophila and Arabidopsis
Source: PLoS Biol. 2022 Sep 13;20(9):e3001775. doi: 10.1371/journal.pbio.3001775 (PMC9470001; doi:10.1371/journal.pbio.3001775)
Supplement: S4 File — (PDF) [file pbio.3001775.s050.pdf]

Gene age and Grantham's distance

af_moutinho

18/10/2021

This script shows the correlation between gene age and the grantham's distance between amino-acids.

```
setwd("~/Dropbox/SupplementaryData_GeneAge/Data")
# change here to the path where you keep the data

# Libraries
library(plyr)
library(dplyr)
library(reshape2)
library(ggplot2)
library(data.table)
library(doBy)
library(data.table)
library(cowplot)
library(knitr)
library(kableExtra)
#

# theme of the plot
theme.plot <- function(x) {
  theme(axis.title = element_text(face = "bold", color = "black", size=14),
        text = element_text(size=14),
        axis.title.x = element_text(margin = margin(t = 18, r = 10, b = 0, l = 0)),
        axis.title.y = element_text(margin = margin(t = 18, r = 10, b = 0, l = 0)),
        panel.grid.minor=element_blank(),
        panel.grid.major = element_line(colour = "grey", linetype = "dashed", size = 0.2),
        panel.grid.major.y=element_blank(),
        #strip.text.y = element_blank(),
        axis.text.x = element_text(angle = 60, hjust = 1))
}

# calling data tables
data.files <- list.files(".", ".csv")

# reading each table into a list
data.list <- lapply(data.files, fread, header = TRUE, sep = "\t")

# keeping only geneID and grantham's distance between aa's
granthamD.age <- lapply(data.list, function(x) {
  subset(x, select = c("GeneID", "Clade", "mean.DGrantham", "species"))
})
```

```r
granthamD.age2 <- lapply(granthamD.age, function(x) {
  tbl <- na.omit(unique(x))
})

# saving the table (S24_Data):
granthamD_age_df <- rbindlist(granthamD.age2)
write.table(granthamD_age_df, file = "../GranthamsDistance/S27_Data.csv", sep = "\t",
            col.names = T, row.names = F, quote = F)

# summarizing the mean grantham's distance per gene for each clade
fun.q <- function(x) {
  q <- quantile(x$mean.DGrantham, na.rm = TRUE)
  median <- q[[3]]
  firstQ <- q[[2]]
  thirdQ <- q[[4]]
  tbl <- data.frame(median, firstQ, thirdQ)
}

sum.granthamD.age <- lapply(granthamD.age2, function(x) {
  ddply(x, c("Clade", "species"), fun.q)
})

# plotting each of the output tables
plot.age.granthamD <- lapply(sum.granthamD.age, function(x) {
  ggplot(x, aes(x = Clade, y = median)) +
  geom_line(col = "black", size = 0.2)+
  geom_ribbon(aes(ymax=thirdQ, ymin=firstQ), alpha=0.2) +
  geom_point(size=.2)+
  #geom_smooth(method = "glm", formula = y~x, se = FALSE) +
  scale_x_continuous(breaks = c(1,2,3,4,5,6,7,8,9,10,11,12,13,14,15)) +
  ylab("Grantham's distance") +
  xlab("Phylostrata") +
  #scale_x_sqrt() +
  scale_fill_grey() +
  scale_color_grey() +
  #ggtitle(as.character(x$species)) +
  theme_bw() +
  theme.plot()
})
plot.age.granthamD
```

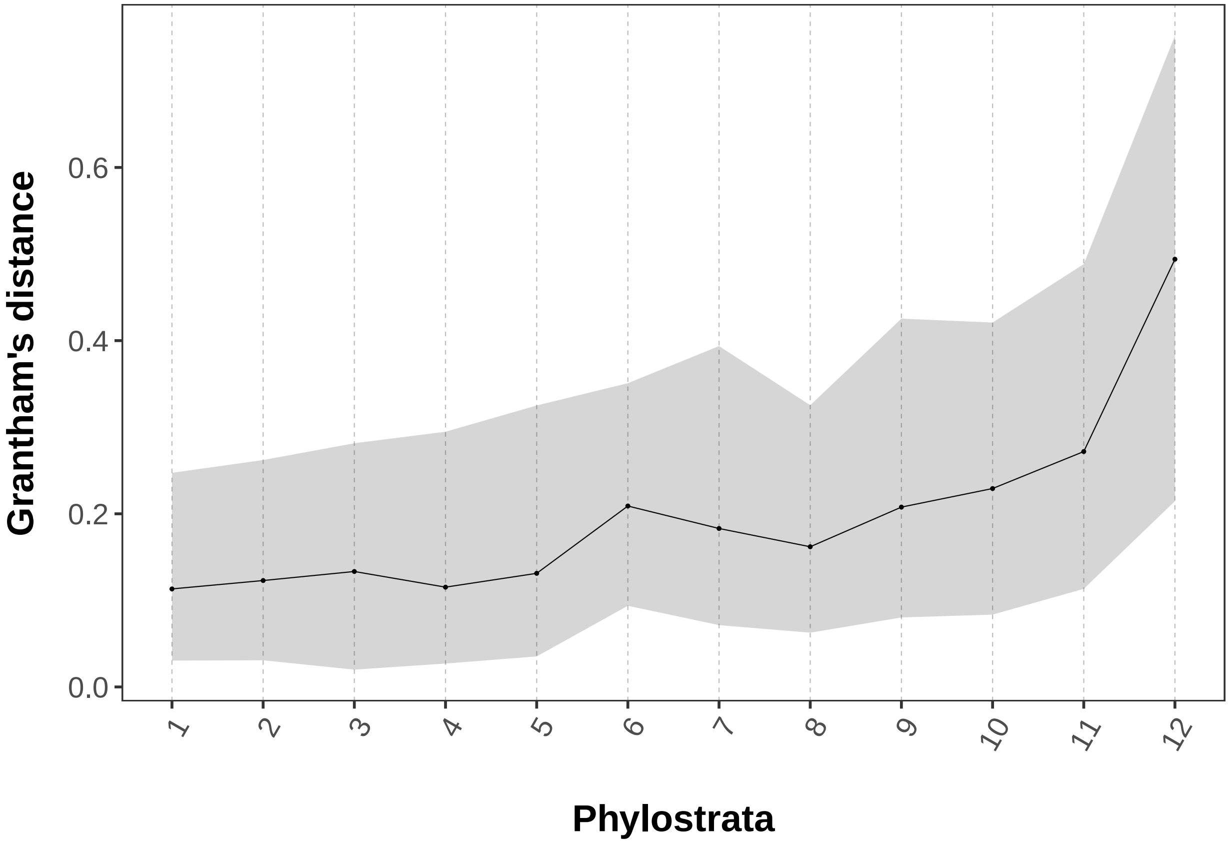
Grantham's distance
0.6
0.4
0.2
0.0
1
2
3
4
5
6
7
8
9
10
11
12
Phylostrata

[[1]]

Table 1: Correlation between gene age and Grantham's distance between aminoacids in Drosophila

	species	Kendall.tau	p.value
tau	Drosophila	0.7878788	0.0003628

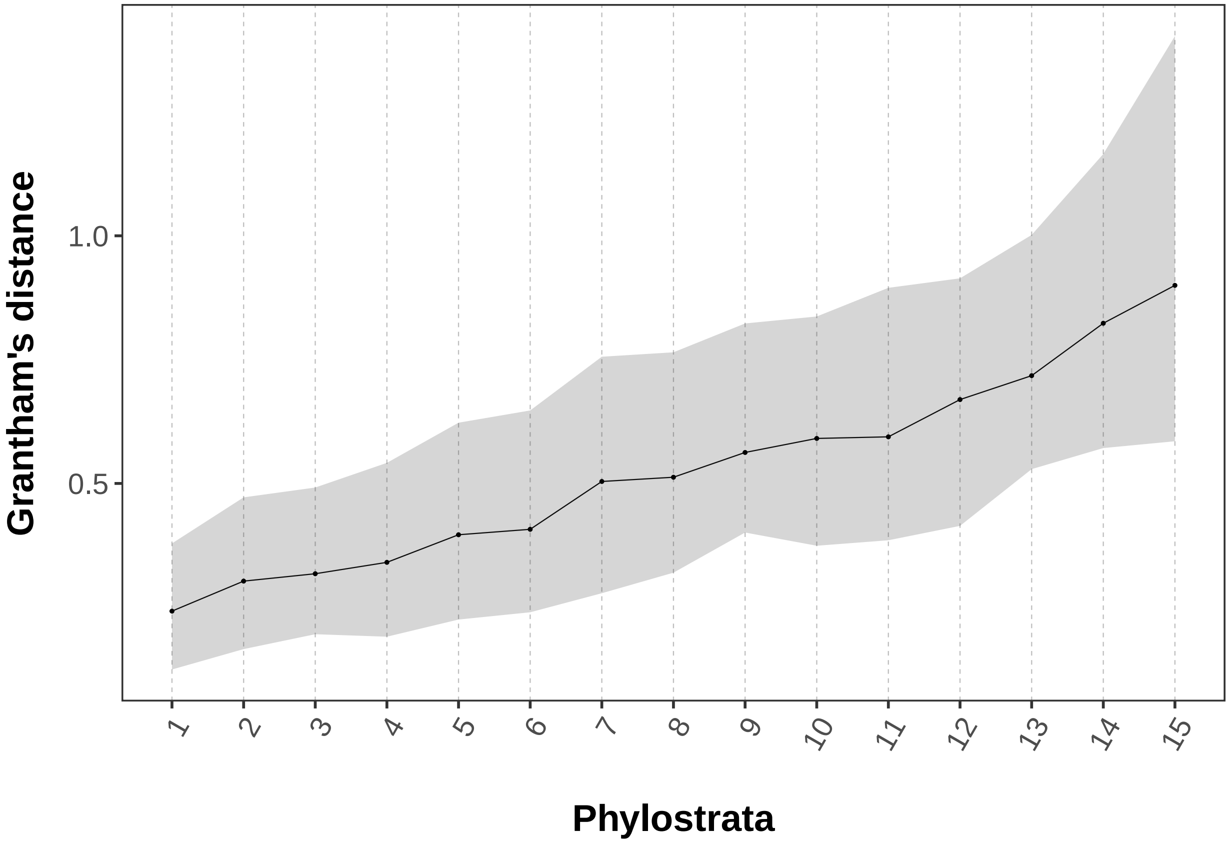

[[2]]

```
# estimating the correlations
stats.age.grantham <- lapply(sum.granthamD.age, function(x) {
  cor.df <- cor.test(x$Clade, x$median, method = "kendall", exact = FALSE)
  species <- unique(x$species)
  Kendall.tau <- cor.df$estimate
  p.value <- cor.df$p.value
  data.frame(species, Kendall.tau, p.value)
})

# showing the tables
for(i in stats.age.grantham) {
  print(kable(x = i, caption = paste0("Correlation between gene age
                                      and Grantham's distance between aminoacids in ",
                                      unique(i$species))))
}
```

The next chunk of the script assesses the proportion of adaptive substitutions across Grantham's distances categories in each age class.

Table 2: Correlation between gene age and Grantham's distance between aminoacids in Arabidopsis

	species	Kendall.tau	p.value
tau	Arabidopsis	1	2e-07

```r
setwd("~/Dropbox/SupplementaryData_GeneAge/GranthamsDistance/")

# Libraries
library(ggpubr)
library(cowplot)
#

age_grantham <- read.table(file = "S28_Data.csv",
                           sep = "\t", header = T)
head(age_grantham)
```

variable GranthamD Age species value.mean value.sd N 1 omega[a] 0.05713825 1 Arabidopsis 0.223222 0.01439402 18543 2 omega[a] 0.05713825 2 Arabidopsis 0.165071 0.02712651 19146 3 omega[a] 0.05713825 3 Arabidopsis 0.097627 0.04765551 2060 4 omega[a] 0.05713825 4 Arabidopsis 0.213187 0.04783916 7567 5 omega[a] 0.05713825 5 Arabidopsis 0.210306 0.05124394 2592 6 omega[a] 0.05713825 6 Arabidopsis 0.168169 0.06050969 1758

```r
# estimating the proportion of adaptive and non-adaptive mutations in each Grantham's
# distance category in each age class
age_grantham$G <- age_grantham$GranthamD*age_grantham$value.mean*age_grantham$N

# estimating the weighted average for each age class:
vars_df <- ddply(age_grantham, c("species", "variable", "Age"), function(x) {
  sum.G <- sum(x$G)
  sum.N <- sum(x$N)
  G_weighted <- sum.G/sum.N
  data.frame(G_weighted)
})

# plotting the relationship between Pa with gene age:
omega_a_df <- subset(vars_df, variable == "omega[a]")

plot_Ga_age <- ggplot(omega_a_df, aes(Age, G_weighted)) +
  geom_point(size = 0.8) +
  geom_smooth(formula = y~x, method = "lm", se = F) +
  stat_cor(label.y.npc="top", label.x.npc = "left", method = "pearson", size = 3) +
  ylab(expression(italic(bar(G)[a]))) +
  xlab("Phylostrata level") +
  facet_wrap(.~species, scales = "free_y", labeller = label_parsed) +
  theme_bw()

# plotting the relationship between Pna with gene age:
omega_na_df <- subset(vars_df, variable == "omega[na]")

plot_Gna_age <- ggplot(omega_na_df, aes(Age, G_weighted)) +
  geom_point(size = 0.8) +
  geom_smooth(formula = y~x, method = "lm", se = F) +
```

```
  stat_cor(label.y.npc="top", label.x.npc = "left", method = "pearson", size = 3) +
  ylab(expression(italic(bar(G)[na]))) +
  xlab("Phylostrata level") +
  facet_wrap(.~species, scales = "free_y", labeller = label_parsed) +
  theme_bw()

# combining the two plots:
plot_Ga_Gna <- plot_grid(plot_Ga_age + xlab("") +
                           theme(plot.margin = unit(c(0.1, 0.1, 0, 0.1), "cm")),
                         plot_Gna_age +
                           theme(plot.margin = unit(c(0, 0.1, 0, 0.1), "cm")),
                         nrow = 2, align = "hv")
plot_Ga_Gna
```

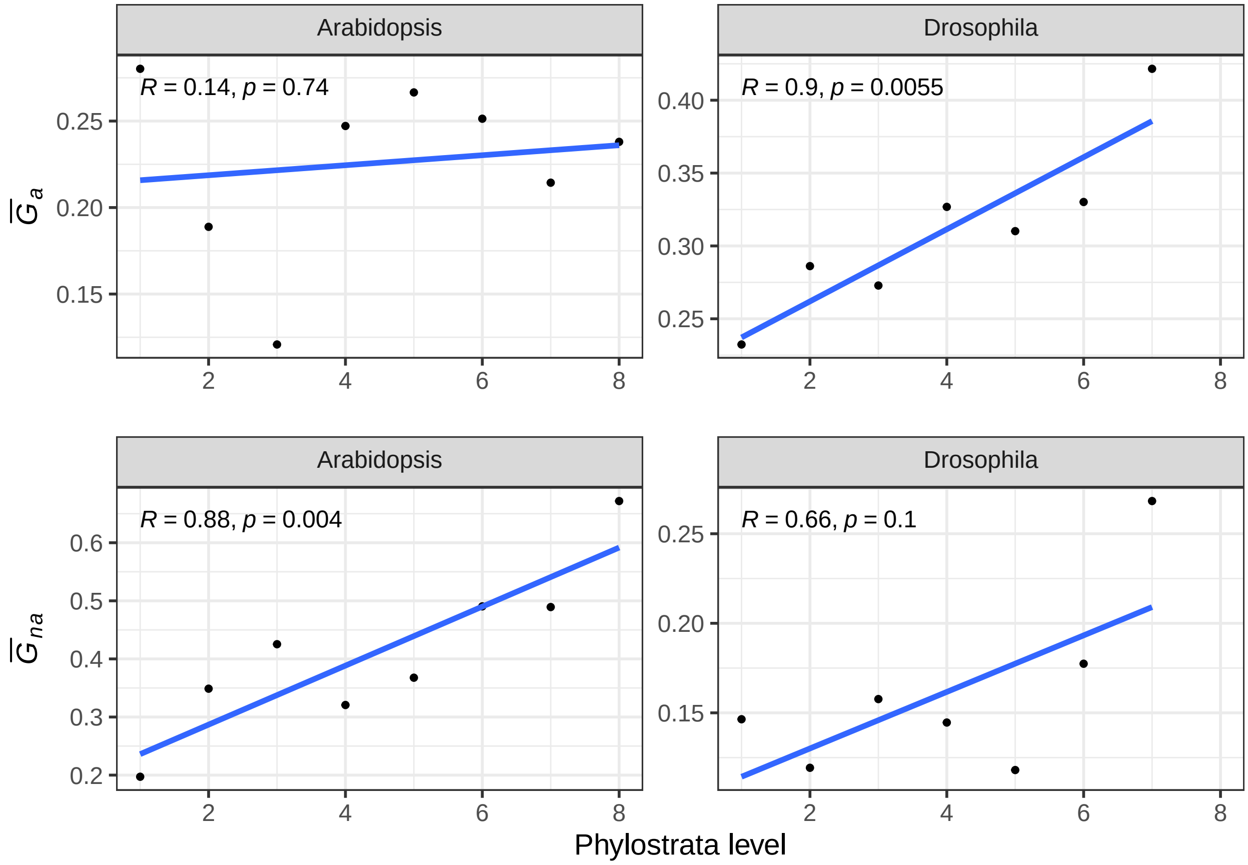

The next chunk of the script assess whether the correlation between gene age and Grantham's distances were dependent on the level of intrinsic disorder of proteins. This analysis was not included in the main text and was only performed as an extra check of the relationship between Grantham's distances and gene age.

```
# Gene Age vs. Protein Intrinsic Disorder vs. Grantham's Distance
Grantham.age <- lapply(data.list, function(x) {
  na.omit(unique(subset(x, select = c("GeneID", "Clade", "mean.DGrantham", "mean.dis",
                                      "cat.dis", "species"))))
})

# function to estimate the median Grantham's distance in ache protein disorder
# category for each age class
```

```r
fun.q <- function(x) {
  q <- quantile(x$mean.DGrantham, na.rm = TRUE)
  median <- q[[3]]
  firstQ <- q[[2]]
  thirdQ <- q[[4]]
  tbl <- data.frame(median, firstQ, thirdQ)
}

# applying the function
sum.granthamD.age <- lapply(Grantham.age, function(x) {
  ddply(x, c("Clade", "cat.dis", "species"), fun.q)
})

# changing the values of the category from c(1,2) to c("Low", "High")
sum.granthamD.age <- lapply(sum.granthamD.age, function(x) {
  x$cat.dis <- factor(x$cat.dis, levels = c(1,2))
  levels(x$cat.dis) <- c("Low", "High")
  return(x)
})

# combing the tables from the two species
sum.granthamD.age.dis <- rbindlist(sum.granthamD.age)
colnames(sum.granthamD.age.dis)[2] <- "Disorder"

# plotting
plot.age.granthamD <- ggplot(sum.granthamD.age.dis, aes(x = Clade, y = median,
                                                        fill = Disorder)) +
  geom_line(col = "black", size = 0.2)+
  geom_ribbon(aes(ymax=thirdQ, ymin=firstQ, fill = Disorder), alpha=0.6) +
  geom_point(size=.2)+
  scale_x_continuous(breaks = c(1,2,3,4,5,6,7,8,9,10,11,12,13,14,15)) +
  ylab("Grantham's distance") +
  xlab("Phylostrata") +
  #scale_x_sqrt() +
  scale_fill_grey() +
  scale_color_grey() +
  facet_grid(.~species, scales = "free_x", labeller = label_parsed) +
  theme_bw() +
  theme.plot()
plot.age.granthamD
```

Table 3: Correlation between gene age and Grantham's distance for each category of protein disorder in Drosophila

cat.dis	species	Kendall.tau	p.value
Low	Drosophila	0.6363636	0.0039762
High	Drosophila	0.6060606	0.0060899

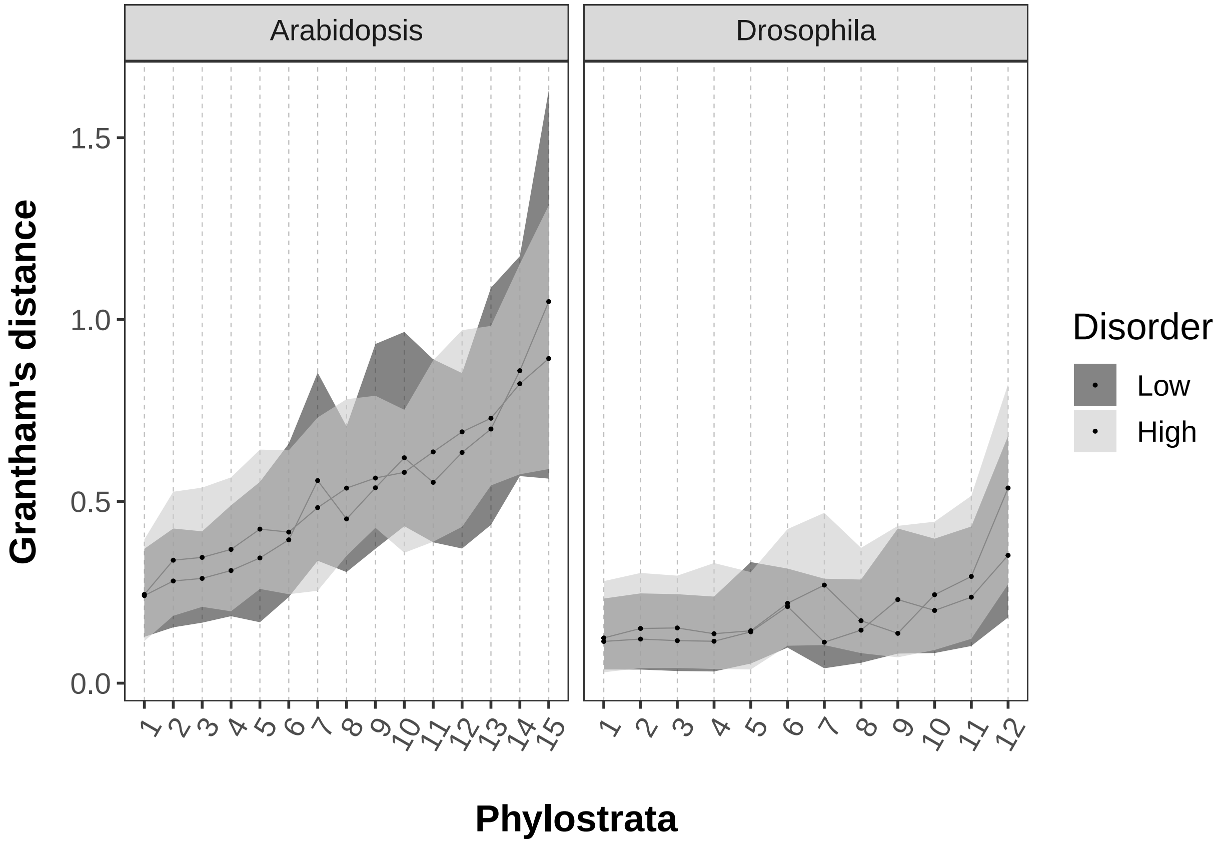


```
# estimating the correlations
stats.GD.dis <- lapply(sum.granthamD.age, function(x) {
  ddply(x, c("cat.dis"), function(a) {
    cor.df <- cor.test(a$Clade, a$median, method = "kendall", exact = FALSE)
    species <- unique(x$species)
    Kendall.tau <- cor.df$estimate
    p.value <- cor.df$p.value
    data.frame(species, Kendall.tau, p.value)
  })
})

for(i in stats.GD.dis) {
  print(kable(x = i, caption = paste0("Correlation between gene age
                                      and Grantham's distance for each category
                                      of protein disorder in ", unique(i$species))))
}
```

Table 4: Correlation between gene age and Grantham's distance for each category of protein disorder in Arabidopsis

cat.dis	species	Kendall.tau	p.value
Low	Arabidopsis	0.9238095	1.6e-06
High	Arabidopsis	0.9809524	3.0e-07
