# Supplementary material for: Strong evidence for the adaptive walk model of gene evolution in Drosophila and Arabidopsis
Source: PLoS Biol. 2022 Sep 13;20(9):e3001775. doi: 10.1371/journal.pbio.3001775 (PMC9470001; doi:10.1371/journal.pbio.3001775)
Supplement: S5 File — (PDF) [file pbio.3001775.s051.pdf]

Protein association analysis

af_moutinho

23/04/2021

This script shows the analysis performed with protein association data.

```
setwd("~/Dropbox/SupplementaryData_GeneAge/PPI/")
# change here to the respective folder where you keep the data

# Libraries
library(plyr)
library(dplyr)
library(data.table)
library(ggplot2)
library(reshape2)
library(doBy)
library(knitr)
library(kableExtra)
library(agricolae)
#

# theme of the plot
theme.plot <- function(x) {
  theme(axis.title = element_text(face = "bold", color = "black", size=14),
        text = element_text(size=14),
        axis.title.x = element_text(margin = margin(t = 18, r = 10, b = 0, l = 0)),
        axis.title.y = element_text(margin = margin(t = 18, r = 10, b = 0, l = 0)),
        panel.grid.minor=element_blank(),
        panel.grid.major = element_line(colour = "grey", linetype = "dashed", size = 0.2),
        panel.grid.major.y=element_blank(),
        #strip.text.y = element_blank(),
        axis.text.x = element_text(angle = 60, hjust = 1))
}
#

df_prot_links <- read.table(file = "S29_Data.csv", sep = "\t", header = TRUE)
df_prot_links$Clade <- as.character(df_prot_links$Clade)

df_prot_links$Clade <- factor(df_prot_links$Clade,
                              levels = c("1", "2", "3", "4", "5", "6",
                                         "7", "8", "9", "10", "11", "12"))
df_prot_links <- na.omit(df_prot_links)
# doing a box plot with the distribution of the combined scores for ach protein pair in each clade:
### all prot links:
plot.scores.age <- ggplot(df_prot_links, aes(Clade, combined_score)) +
  geom_point(size = 0.1) +
```

```r
  geom_boxplot(stat = "boxplot", position = "dodge", outlier.size = 0.01) +
  scale_y_sqrt() +
  xlab("Clade") +
  ylab("Combined scores of PPI") +
  theme.plot() +
  theme_bw()
plot.scores.age
```

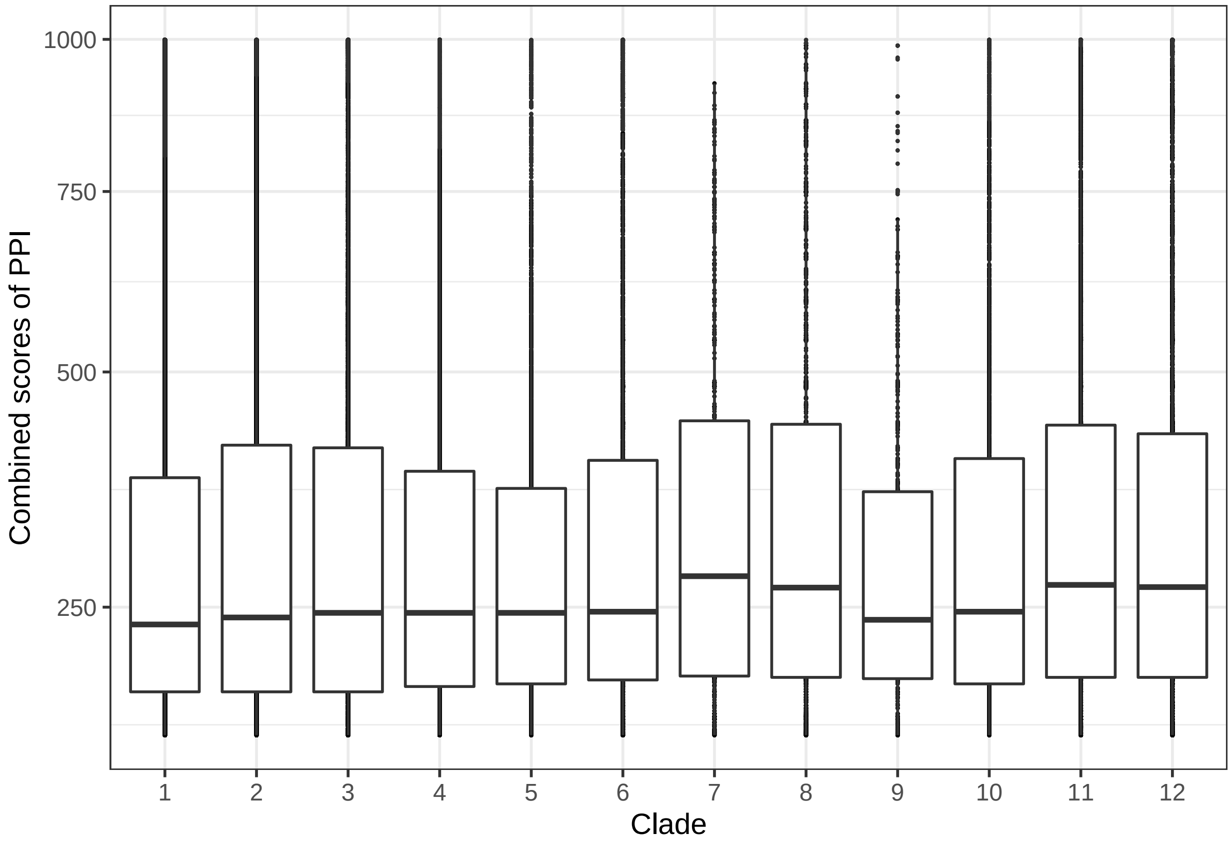


```r
### statistical analyses:
kruskal.test(combined_score ~ Clade, df_prot_links)
```

```
Kruskal-Wallis rank sum test
```

data: combined_score by Clade Kruskal-Wallis chi-squared = 753.07, df = 11, p-value < 2.2e-16

```r
stats_links <- kruskal(df_prot_links$combined_score, df_prot_links$Clade, p.adj = "fdr", group = TRUE)
stat_group <- stats_links$groups
stat_group$Clade <- rownames(stat_group)

# presenting the table:
kable(x = stat_group, caption = "Statistical group for the combined scores in each clade")
```

Table 1: Statistical group for the combined scores in each clade

	df_prot_links$combined_score	groups	Clade
7	446431.9	a	7
11	438037.7	a	11
12	434399.6	a	12
8	433011.5	a	8
6	414336.9	b	6
3	412601.4	b	3
10	412129.4	bc	10
2	412099.4	bc	2
4	406057.7	cd	4
5	401936.3	de	5
1	400438.9	e	1
9	390720.1	e	9
