# Supplementary material for: Strong evidence for the adaptive walk model of gene evolution in Drosophila and Arabidopsis
Source: PLoS Biol. 2022 Sep 13;20(9):e3001775. doi: 10.1371/journal.pbio.3001775 (PMC9470001; doi:10.1371/journal.pbio.3001775)
Supplement: S6 File — (PDF) [file pbio.3001775.s052.pdf]

co-factor_continuous

af_moutinho

19/03/2021

This script includes the data analysis of the gene age data together with the analysed co-factors:
co.factors[[1]] <- protein intrinsic disorder co.factors[[2]] <- gene expression
co.factors[[3]] <- protein length
co.factors[[4]] <- mean relative solvent accessability per gene

The first chunk of the scipt will show the correlations between gene age and each of the co-factors

```
setwd("~/Dropbox/SupplementaryData_GeneAge/Data/")
# change here to the respective folder where you keep the data

# Libraries
library(plyr)
library(dplyr)
library(data.table)
library(ggplot2)
library(reshape2)
library(doBy)
library(knitr)
library(kableExtra)
#

# theme of the plot
theme.plot <- function(x) {
  theme(axis.title = element_text(face = "bold", color = "black", size=14),
        text = element_text(size=14),
        axis.title.x = element_text(margin = margin(t = 18, r = 10, b = 0, l = 0)),
        axis.title.y = element_text(margin = margin(t = 18, r = 10, b = 0, l = 0)),
        panel.grid.minor=element_blank(),
        panel.grid.major = element_line(colour = "grey", linetype = "dashed", size = 0.2),
        panel.grid.major.y=element_blank(),
        #strip.text.y = element_blank(),
        axis.text.x = element_text(angle = 60, hjust = 1))
}

# calling data tables
data.files <- list.files(".", ".csv")

# reading each table into a list
data.list <- lapply(data.files, fread, header = TRUE, sep = "\t")


#############################
```

```r
#############################
# Gene Age vs. Protein Length
length.age <- lapply(data.list, function(x) {
  na.omit(unique(subset(x, select = c("GeneID", "Clade", "Length", "species"))))
})

# saving the table S2 Data
length_age_df <- rbindlist(length.age)
write.table(length_age_df, file = "../Co-factors_continuous/S2_Data.csv", sep = "\t",
            col.names = T, row.names = F, quote = F)


# summarizing protein length data for each clade
fun.q <- function(x) {
  q <- quantile(x$Length, na.rm = TRUE)
  firstQ <- q[[2]]
  thirdQ <- q[[4]]
  med.length <- q[[3]]
  tbl <- data.frame(med.length, firstQ, thirdQ)
}

sum.length.age <- lapply(length.age, function(x) {
  ddply(x, c("Clade", "species"), fun.q)
})

# plotting each of the output tables
plot.age.length <- lapply(sum.length.age, function(x) {
  ggplot(x, aes(x = Clade, y = med.length)) +
  geom_line(col = "black", size = 0.2)+
  geom_ribbon(aes(ymax=thirdQ, ymin=firstQ), alpha=0.2) +
  geom_point(size=.2)+
  ylab("") +
  xlab("Clade") +
  #scale_x_sqrt() +
  scale_fill_grey() +
  scale_color_grey() +
  ggtitle(as.character(x$species)) +
  theme_bw() +
  theme.plot()
})
plot.age.length
```

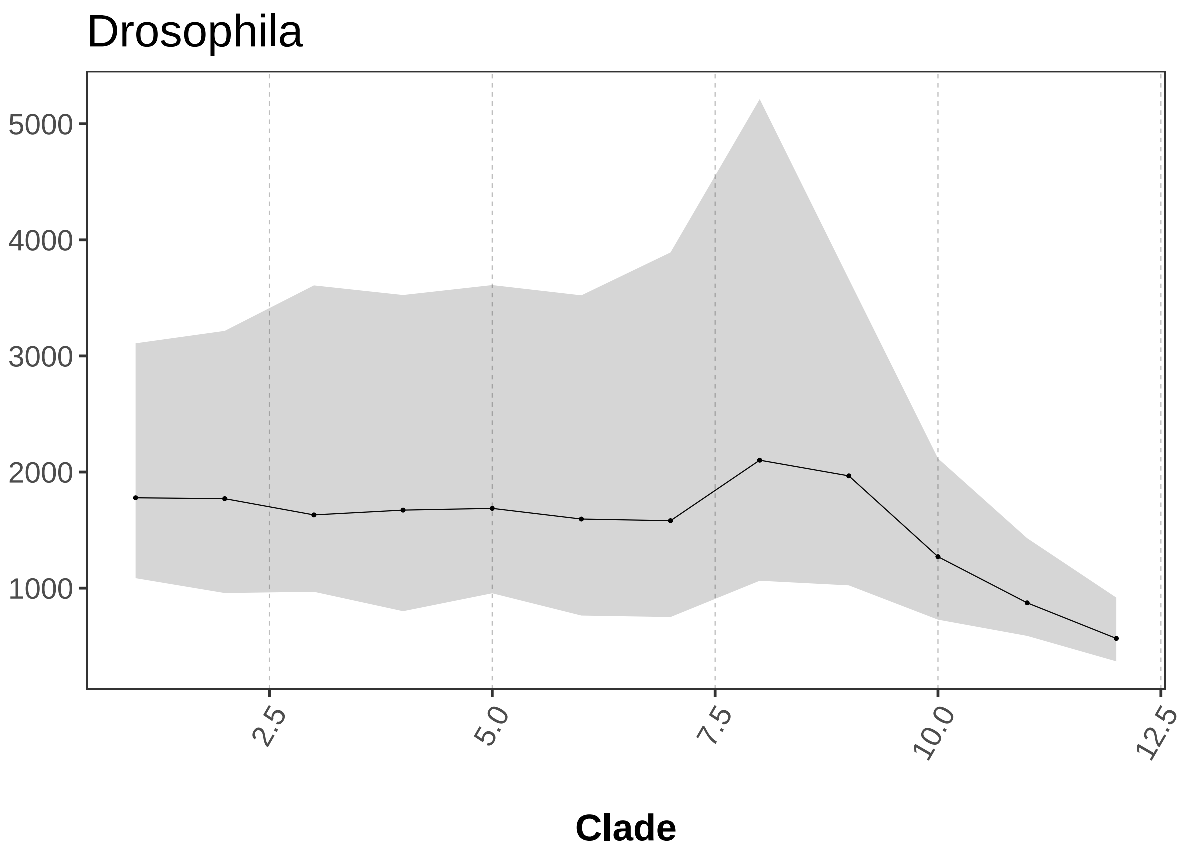

[[1]]

Table 1: Correlation between gene age and protein length in Drosophila

	species	Kendall.tau	p.value
tau	Drosophila	-0.4848485	0.0282123

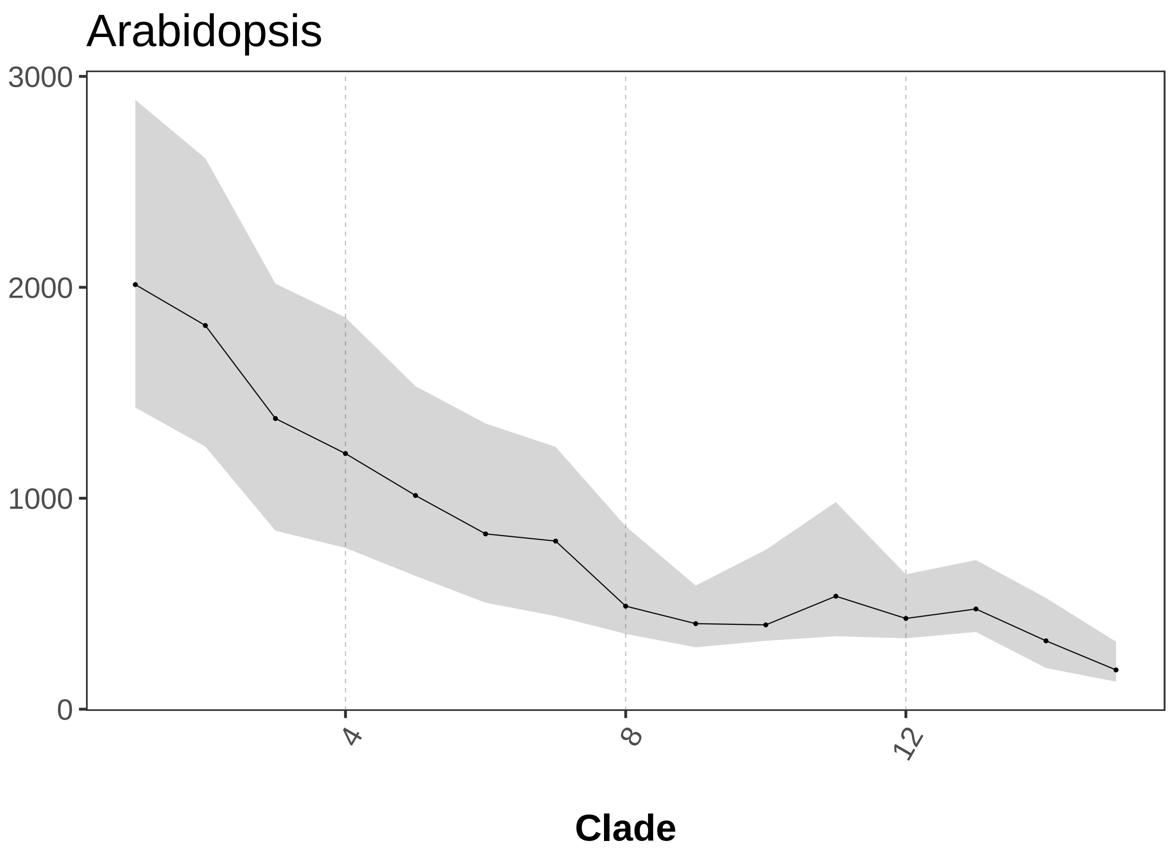

[[2]]

```
# estimating the correlations
stats.age.length <- lapply(sum.length.age, function(x) {
  cor.df <- cor.test(x$Clade, x$med.length, method = "kendall", exact = FALSE)
  species <- unique(x$species)
  Kendall.tau <- cor.df$estimate
  p.value <- cor.df$p.value
  data.frame(species, Kendall.tau, p.value)
})

# showing the tables
for(i in stats.age.length) {
  print(kable(x = i, caption = paste0("Correlation between gene age
                                       and protein length in ", unique(i$species))))
}
```

```
##############################
##############################
# Gene Age vs. Gene Expression
exp.age <- lapply(data.list, function(x) {
```

Table 2: Correlation between gene age and protein length in Arabidopsis

	species	Kendall.tau	p.value
tau	Arabidopsis	-0.847619	1.06e-05

```
  na.omit(unique(subset(x, select = c("GeneID", "Clade", "ExpressionMean", "species"))))
})

# saving the table S3 Data
exp_age_df <- rbindlist(exp.age)
write.table(exp_age_df, file = "../Co-factors_continuous/S3_Data.csv", sep = "\t",
            col.names = T, row.names = F, quote = F)

# summarizing protein length data for each clade
fun.q <- function(x) {
  q <- quantile(x$ExpressionMean, na.rm = TRUE)
  firstQ <- q[[2]]
  thirdQ <- q[[4]]
  med.exp <- q[[3]]
  tbl <- data.frame(med.exp, firstQ, thirdQ)
}

sum.exp.age <- lapply(exp.age, function(x) {
  ddply(x, c("Clade", "species"), fun.q)
})

# plotting each of the output tables
plot.age.exp <- lapply(sum.exp.age, function(x) {
  ggplot(x, aes(x = Clade, y = med.exp)) +
  geom_line(col = "black", size = 0.2)+
  geom_ribbon(aes(ymax=thirdQ, ymin=firstQ), alpha=0.2) +
  geom_point(size=.2)+
  ylab("") +
  xlab("Clade") +
  #scale_x_sqrt() +
  scale_fill_grey() +
  scale_color_grey() +
  ggtitle(as.character(x$species)) +
  theme_bw() +
  theme.plot()
})
plot.age.exp
```

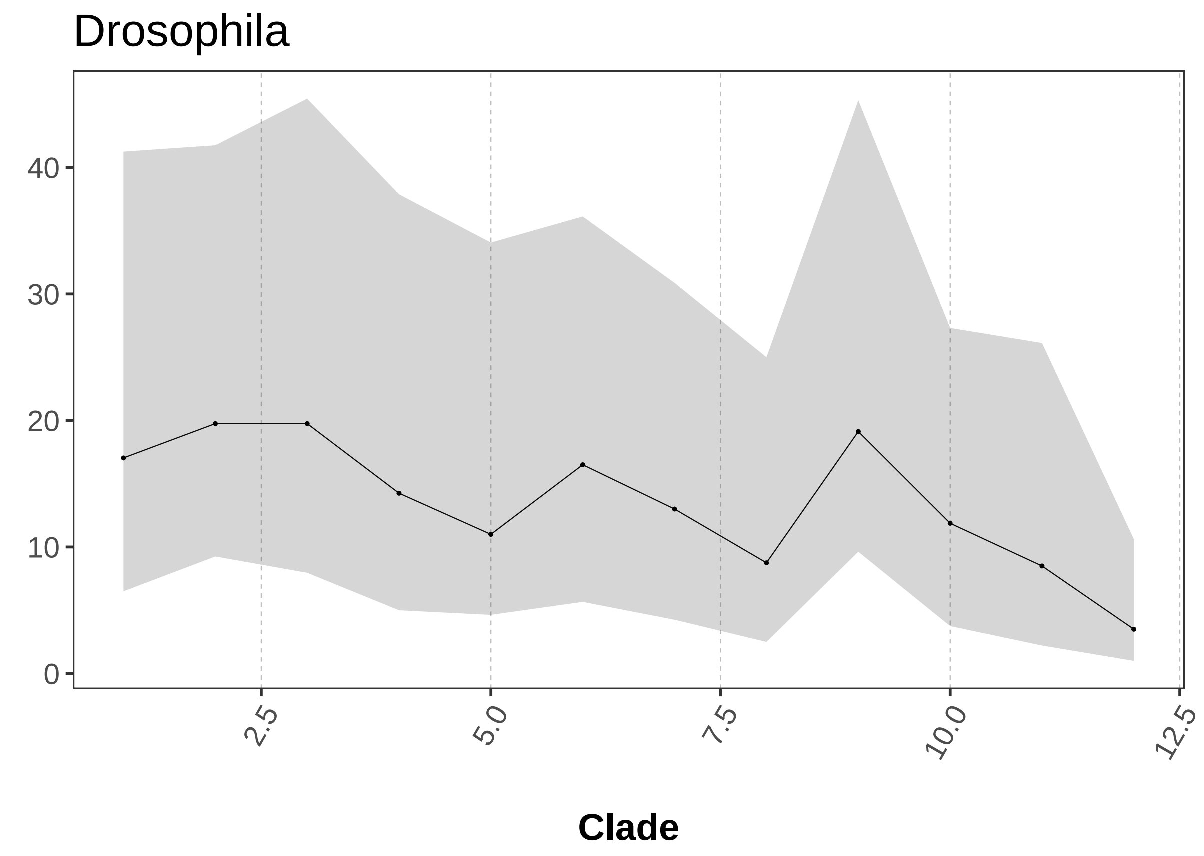

[[1]]

Table 3: Correlation between gene age and gene expression in Drosophila

	species	Kendall.tau	p.value
tau	Drosophila	-0.5954372	0.0073482

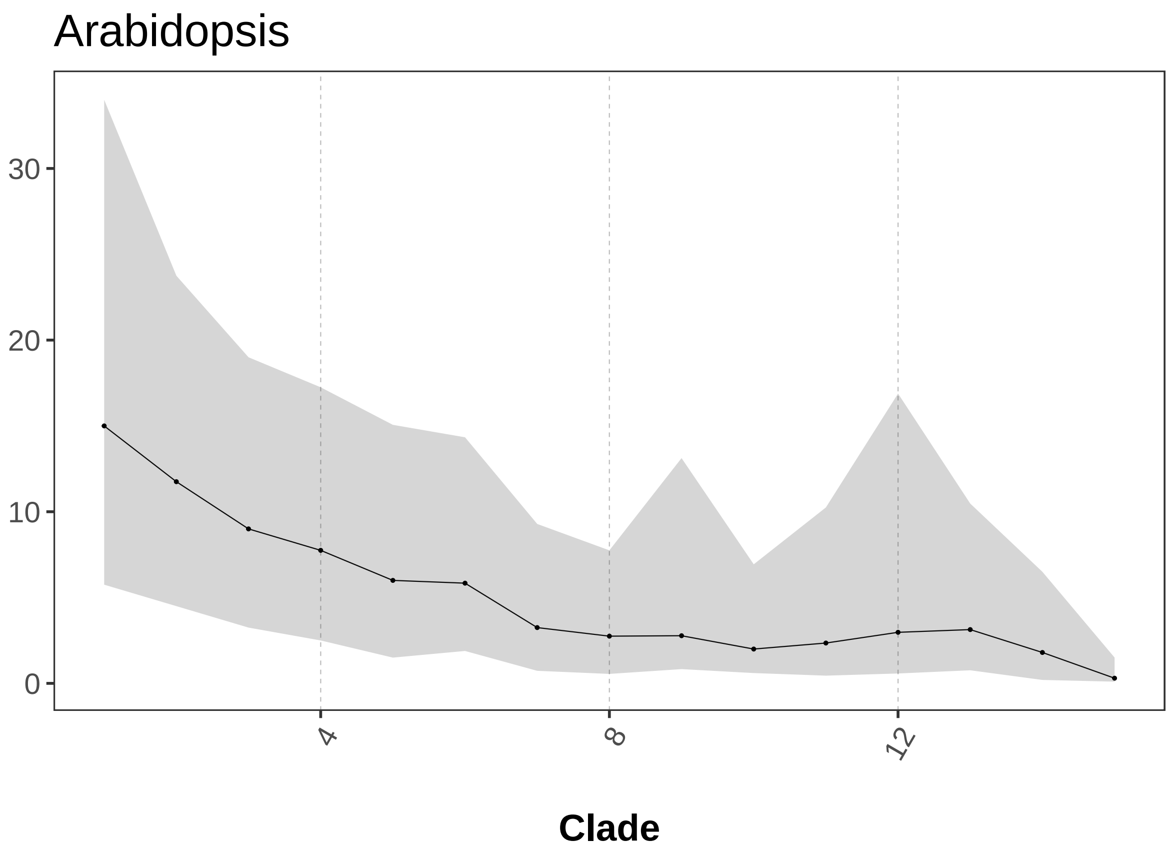

[[2]]

```
# estimating the correlations
stats.age.exp <- lapply(sum.exp.age, function(x) {
  cor.df <- cor.test(x$Clade, x$med.exp, method = "kendall", exact = FALSE)
  species <- unique(x$species)
  Kendall.tau <- cor.df$estimate
  p.value <- cor.df$p.value
  data.frame(species, Kendall.tau, p.value)
})

# showing the tables
for(i in stats.age.exp) {
  print(kable(x = i, caption = paste0("Correlation between gene age
                                      and gene expression in ", unique(i$species))))
}
```

```
##############################
##############################
# Gene Age vs. RSA
rsa.age <- lapply(data.list, function(x) {
```

Table 4: Correlation between gene age and gene expression in Arabidopsis

	species	Kendall.tau	p.value
tau	Arabidopsis	-0.7904762	4e-05

```
  na.omit(unique(subset(x, select = c("GeneID", "Clade", "mean.rsa", "species"))))
})

# saving the table S3 Data
rsa_age_df <- rbindlist(rsa.age)
write.table(rsa_age_df, file = "../Co-factors_continuous/S4_Data.csv", sep = "\t",
            col.names = T, row.names = F, quote = F)

# summarizing protein length data for each clade
fun.q <- function(x) {
  q <- quantile(x$mean.rsa, na.rm = TRUE)
  firstQ <- q[[2]]
  thirdQ <- q[[4]]
  med.rsa <- q[[3]]
  tbl <- data.frame(med.rsa, firstQ, thirdQ)
}

sum.rsa.age <- lapply(rsa.age, function(x) {
  ddply(x, c("Clade", "species"), fun.q)
})

# plotting each of the output tables
plot.age.rsa <- lapply(sum.rsa.age, function(x) {
  ggplot(x, aes(x = Clade, y = med.rsa)) +
  geom_line(col = "black", size = 0.2)+
  geom_ribbon(aes(ymax=thirdQ, ymin=firstQ), alpha=0.2) +
  geom_point(size=.2)+
  ylab("") +
  xlab("Clade") +
  #scale_x_sqrt() +
  scale_fill_grey() +
  scale_color_grey() +
  ggtitle(as.character(x$species)) +
  theme_bw() +
  theme.plot()
})
plot.age.rsa
```

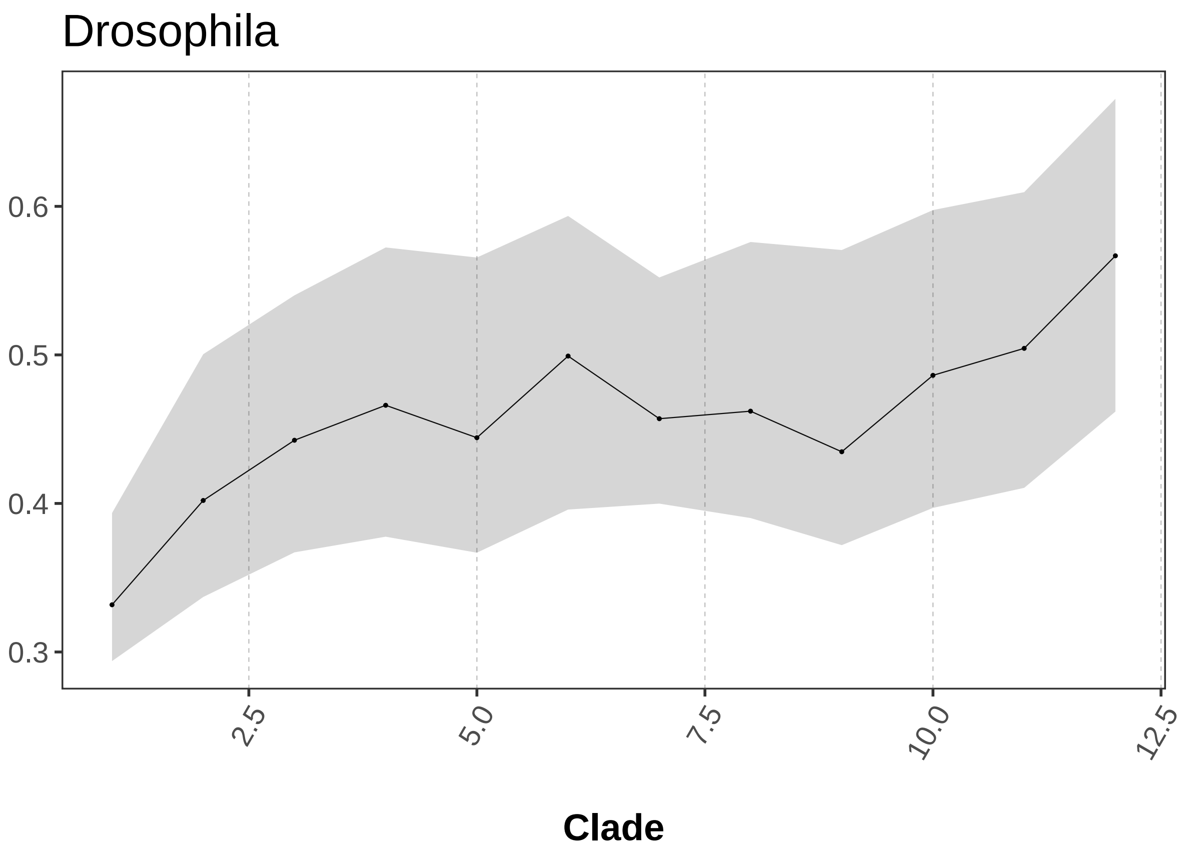

[[1]]

Table 5: Correlation between gene age and rsa in Drosophila

	species	Kendall.tau	p.value
tau	Drosophila	0.6363636	0.0039762

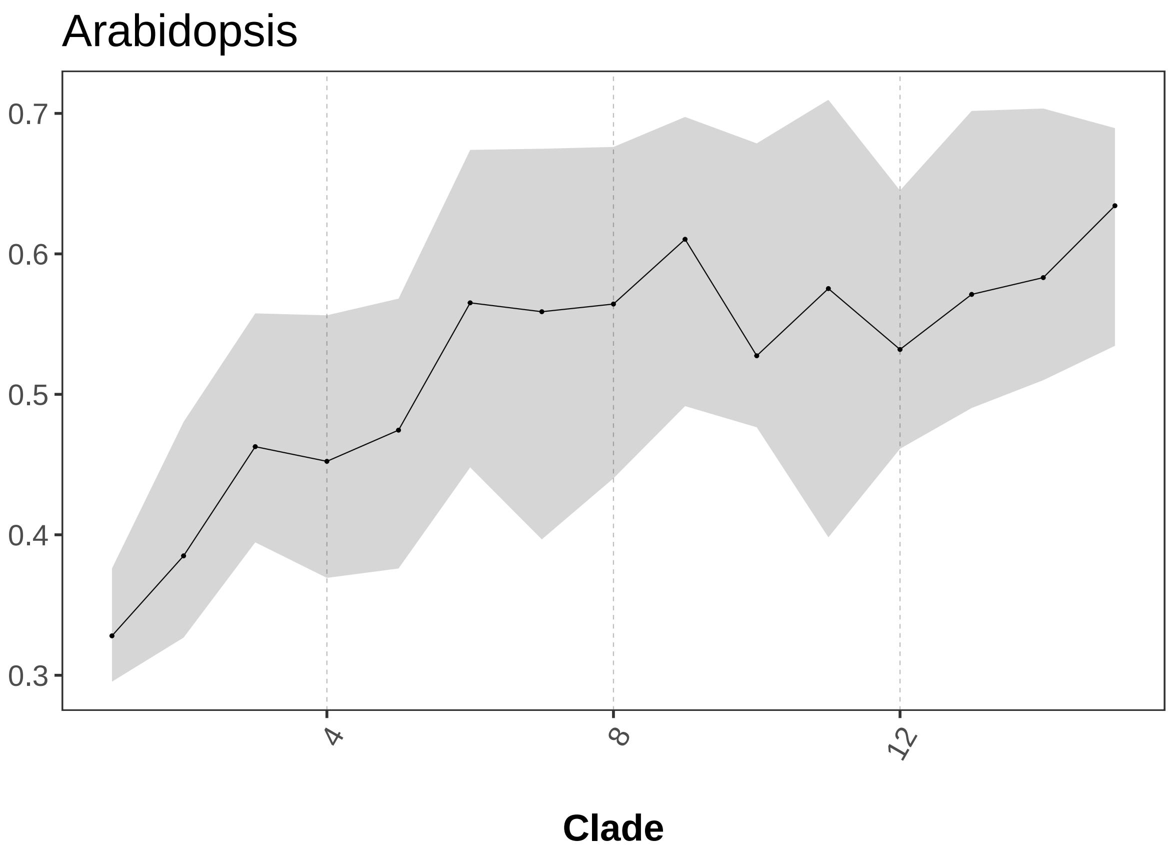

[[2]]

```
# estimating the correlations
stats.age.rsa <- lapply(sum.rsa.age, function(x) {
  cor.df <- cor.test(x$med.rsa, x$Clade, method = "kendall", exact = FALSE)
  species <- unique(x$species)
  Kendall.tau <- cor.df$estimate
  p.value <- cor.df$p.value
  data.frame(species, Kendall.tau, p.value)
})

# showing the tables
for(i in stats.age.rsa) {
  print(kable(x = i, caption = paste0("Correlation between gene age
                                      and rsa in ", unique(i$species))))
}
```

```
##############################
##############################
# Gene Age vs. Protein Intrinsic Disorder
disorder.age <- lapply(data.list, function(x) {
```

Table 6: Correlation between gene age and rsa in Arabidopsis

	species	Kendall.tau	p.value
tau	Arabidopsis	0.6952381	0.0003032

```
  na.omit(unique(subset(x, select = c("GeneID", "Clade", "mean.dis", "species"))))
})

# saving the table S3 Data
dis_age_df <- rbindlist(disorder.age)
write.table(dis_age_df, file = "../Co-factors_continuous/S5_Data.csv", sep = "\t",
            col.names = T, row.names = F, quote = F)

# summarizing protein length data for each clade
fun.q <- function(x) {
  q <- quantile(x$mean.dis, na.rm = TRUE)
  firstQ <- q[[2]]
  thirdQ <- q[[4]]
  med.dis <- q[[3]]
  tbl <- data.frame(med.dis, firstQ, thirdQ)
}

sum.dis.age <- lapply(disorder.age, function(x) {
  ddply(x, c("Clade", "species"), fun.q)
})

# plotting each of the output tables
plot.age.dis <- lapply(sum.dis.age, function(x) {
  ggplot(x, aes(x = Clade, y = med.dis)) +
  geom_line(col = "black", size = 0.2)+
  geom_ribbon(aes(ymax=thirdQ, ymin=firstQ), alpha=0.2) +
  geom_point(size=.2)+
  ylab("") +
  xlab("Clade") +
  #scale_x_sqrt() +
  scale_fill_grey() +
  scale_color_grey() +
  ggtitle(as.character(x$species)) +
  theme_bw() +
  theme.plot()
})
plot.age.dis
```

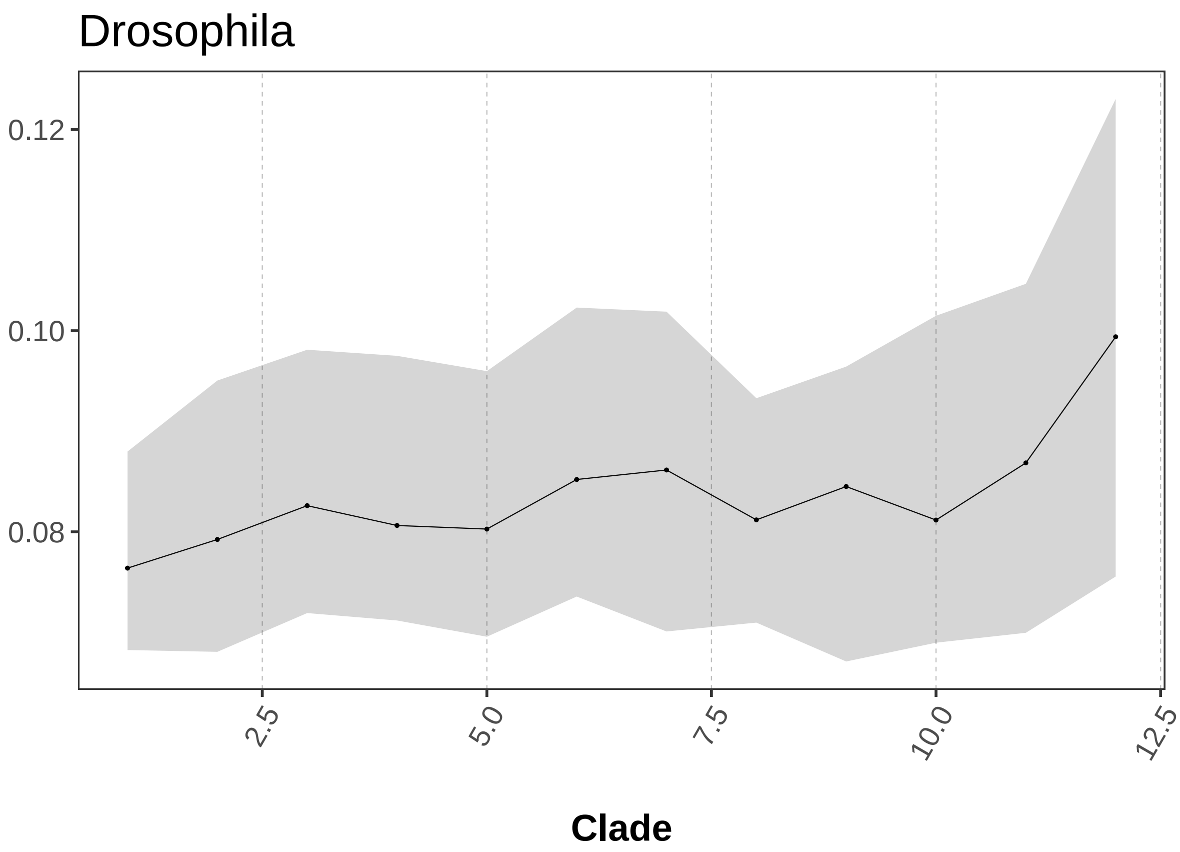

[[1]]

Table 7: Correlation between gene age and disorder in Drosophila

	species	Kendall.tau	p.value
tau	Drosophila	0.6060606	0.0060899

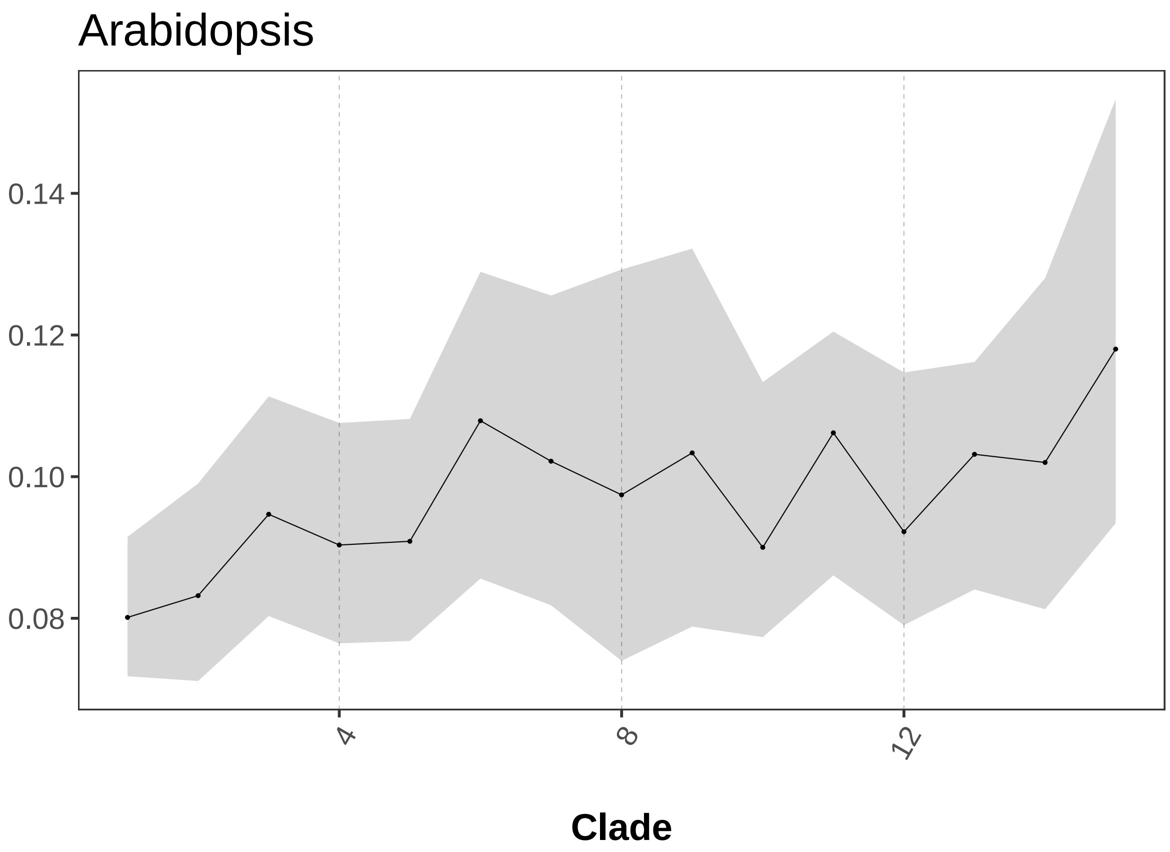

[[2]]

```
# estimating the correlations
stats.age.dis <- lapply(sum.dis.age, function(x) {
  cor.df <- cor.test(x$med.dis, x$Clade, method = "kendall", exact = FALSE)
  species <- unique(x$species)
  Kendall.tau <- cor.df$estimate
  p.value <- cor.df$p.value
  data.frame(species, Kendall.tau, p.value)
})

# showing the tables
for(i in stats.age.dis) {
  print(kable(x = i, caption = paste0("Correlation between gene age
                                       and disorder in ", unique(i$species))))
}
```

```
setwd("~/Dropbox/SupplementaryData_GeneAge/Co-factors_continuous/grapes_analysis/")
# change here to the respective folder where you keep the data

# calling all output tables
```

Table 8: Correlation between gene age and disorder in Arabidopsis

	species	Kendall.tau	p.value
tau	Arabidopsis	0.4666667	0.0153138

```
co.factors <- list.files(".", ".csv")

# reading each table into a list
tbl.list <- lapply(co.factors, read.table, header = TRUE)

# arranging the table for plotting:
tbl.list2 <- lapply(tbl.list, function(x) {
  melt(x, id.vars = c("GeneAge", "category", "co_factor", "species"),
       measure.vars = c("dnds", "omegaNA", "omegaA"))
})

# function to estimate the mean and standard deviation to plot the results with the
# mean of the bootstrap replicates and the 95% confidence interval
fun <- function(x){
  c(mean=mean(x), sd=sd(x))
}

# applying the above function to each output table for each value of each estimate
# (dnds, omegaA, omegaNA) for each value of the variable being analyzed for each species
tbl.sum <- lapply(tbl.list2, function(x) {
  summaryBy(value ~ variable + GeneAge + category + species + co_factor, data=x, FUN = fun)
})

# to change the estimate name to the respective symbol
tbl.sum2 <- lapply(tbl.sum, function(x) {
  ddply(x, c("GeneAge", "category", "co_factor", "species"), function(y) {
    y$variable <- factor(y$variable, levels = c("dnds", "omegaNA", "omegaA"))
    levels(y$variable) <- c(expression(omega), expression(omega[na]),
                                 expression(omega[a]))
    return(y)
  })
})
```

Including Plots

In the next chunk the script to plot the results is represented. The same order will follow.

```
# plotting each of the output tables
plot.co_factors <- lapply(tbl.sum2, function(x) {
  ggplot(x, aes(x = GeneAge, y = value.mean, fill = category)) +
  geom_line(col = "black", size = 0.2)+
  geom_ribbon(aes(ymin=value.mean + 1.96*value.sd,
                  ymax=value.mean - 1.96*value.sd, fill = category), alpha=0.6) +
  geom_point(size=.2)+
  facet_grid(species~variable, scales = "free_x", labeller = label_parsed) +
  ylab("") +
```

```
  xlab("Gene Age") +
  #scale_x_sqrt() +
  scale_fill_grey() +
  scale_color_grey() +
  theme_bw() +
  ggtitle(as.character(x$co_factor)) +
  theme.plot()
})

plot.co_factors
```

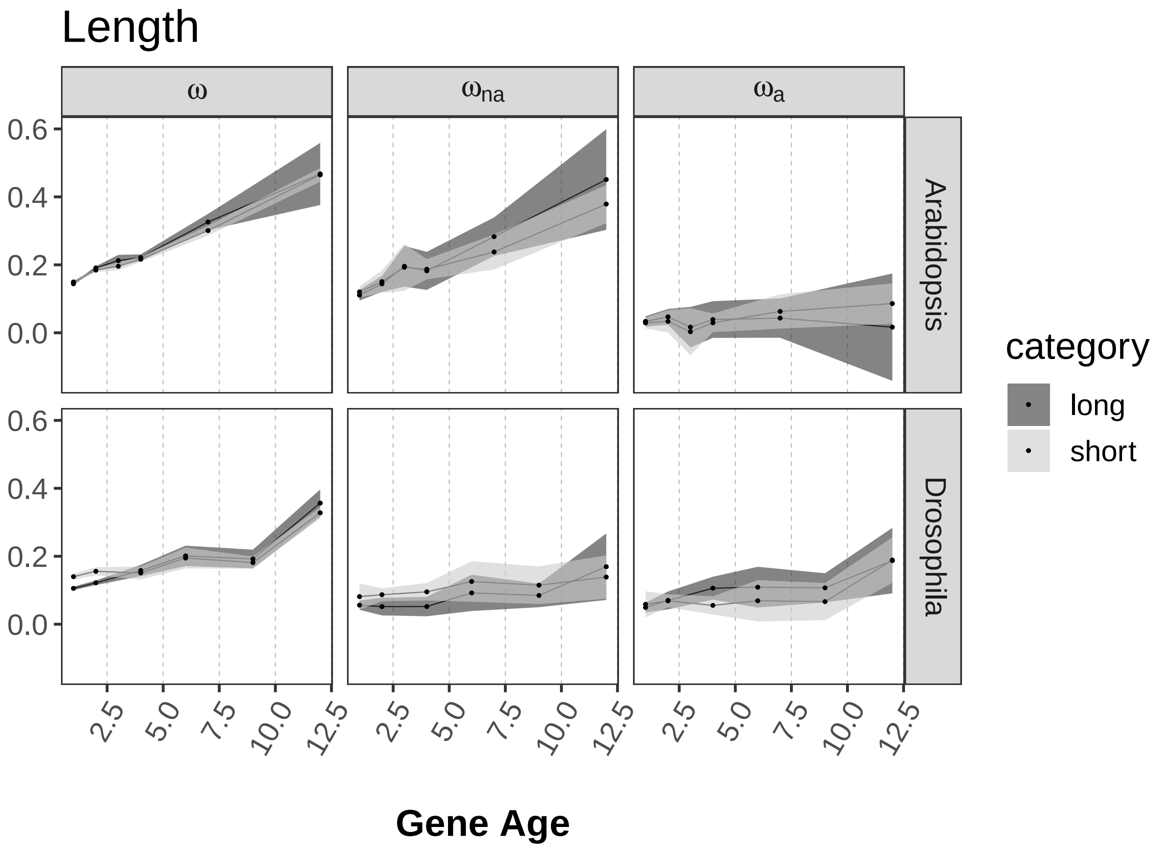

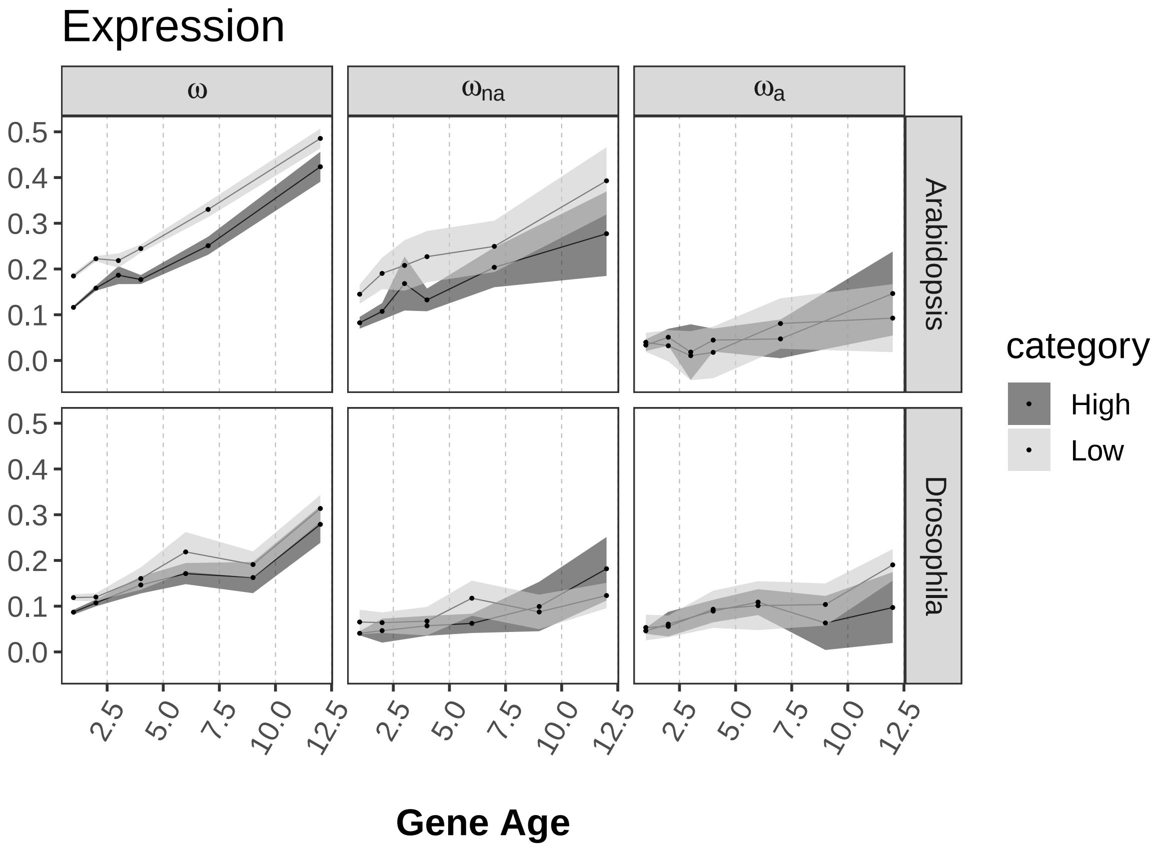

Expression
ω
ω_{na}
ω_a
Arabidopsis
Drosophila
0.5
0.4
0.3
0.2
0.1
0.0
2.5
5.0
7.5
10.0
12.5
Gene Age
category
High
Low

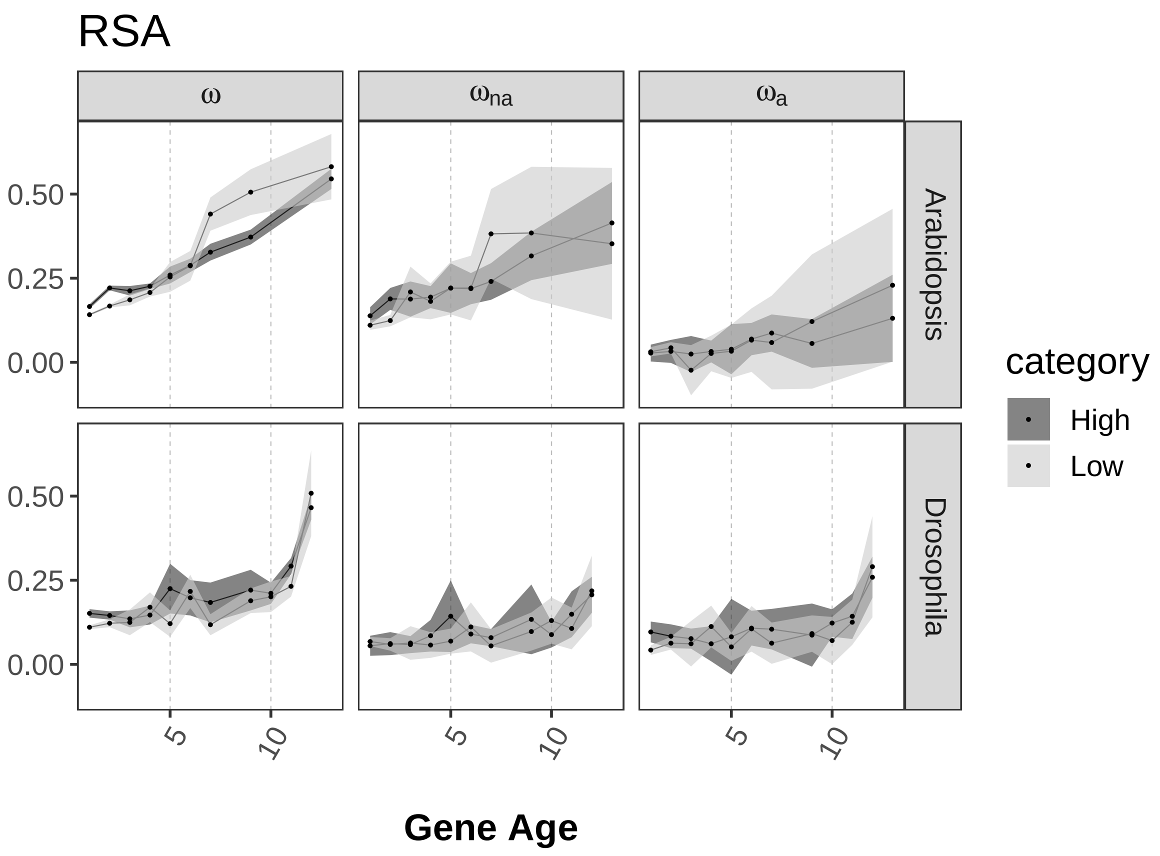

RSA
ω
ωna
ωa
Arabidopsis
Drosophila
0.50
0.25
0.00
5
10
Gene Age
category
High
Low

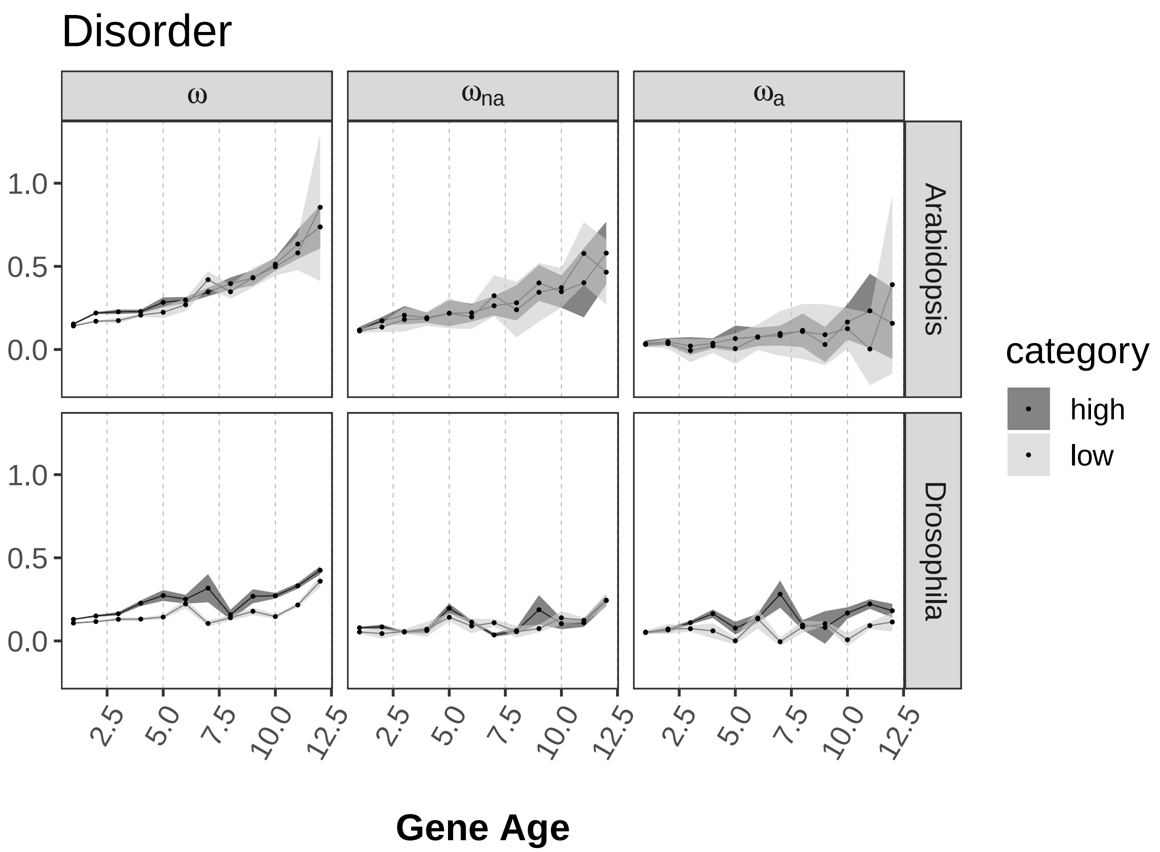

The last section shows how the statistical analyses were performed.

```
tbl.stat <- lapply(tbl.sum2, function(x) {
  ddply(x, c("co_factor", "species", "variable", "category"), function(x) {
    var <- as.numeric(factor(x$GeneAge))
    variable.value <- as.numeric(factor(x$value.mean))
    corr = cor.test(var, variable.value, method = "kendall", exact = FALSE)
    Kendall.tau = corr$estimate
    p.value = corr$p.value
    dat = data.frame(Kendall.tau, p.value)
  })
})

# showing the tables
for(i in tbl.stat) {
  print(kable(x = i, caption = paste0("Statistics for ", unique(i$co_factor))))
}
```

Table 9: Statistics for Length

co_factor	species	variable	category	Kendall.tau	p.value
Length	Arabidopsis	omega	long	1.0000000	0.0048322
Length	Arabidopsis	omega	short	1.0000000	0.0048322
Length	Arabidopsis	omega[na]	long	0.8666667	0.0145950
Length	Arabidopsis	omega[na]	short	0.8666667	0.0145950
Length	Arabidopsis	omega[a]	long	-0.2000000	0.5730251
Length	Arabidopsis	omega[a]	short	0.6000000	0.0908739
Length	Drosophila	omega	long	0.8666667	0.0145950
Length	Drosophila	omega	short	0.7333333	0.0387775
Length	Drosophila	omega[na]	long	0.6000000	0.0908739
Length	Drosophila	omega[na]	short	0.8666667	0.0145950
Length	Drosophila	omega[a]	long	0.8666667	0.0145950
Length	Drosophila	omega[a]	short	0.4666667	0.1884860

Table 10: Statistics for Expression

co_factor	species	variable	category	Kendall.tau	p.value
Expression	Arabidopsis	omega	High	0.8666667	0.0145950
Expression	Arabidopsis	omega	Low	0.8666667	0.0145950
Expression	Arabidopsis	omega[na]	High	0.8666667	0.0145950
Expression	Arabidopsis	omega[na]	Low	1.0000000	0.0048322
Expression	Arabidopsis	omega[a]	High	0.4666667	0.1884860
Expression	Arabidopsis	omega[a]	Low	0.3333333	0.3475580
Expression	Drosophila	omega	High	0.8666667	0.0145950
Expression	Drosophila	omega	Low	0.8666667	0.0145950
Expression	Drosophila	omega[na]	High	1.0000000	0.0048322
Expression	Drosophila	omega[na]	Low	0.7333333	0.0387775
Expression	Drosophila	omega[a]	High	0.6000000	0.0908739
Expression	Drosophila	omega[a]	Low	1.0000000	0.0048322

Table 11: Statistics for RSA

co_factor	species	variable	category	Kendall.tau	p.value
RSA	Arabidopsis	omega	High	0.9444444	0.0003930
RSA	Arabidopsis	omega	Low	1.0000000	0.0001746
RSA	Arabidopsis	omega[na]	High	0.8888889	0.0008492
RSA	Arabidopsis	omega[na]	Low	0.7777778	0.0035093
RSA	Arabidopsis	omega[a]	High	0.7222222	0.0067144
RSA	Arabidopsis	omega[a]	Low	0.6666667	0.0123434
RSA	Drosophila	omega	High	0.6363636	0.0064351
RSA	Drosophila	omega	Low	0.6363636	0.0064351
RSA	Drosophila	omega[na]	High	0.6727273	0.0039711
RSA	Drosophila	omega[na]	Low	0.4909091	0.0355579
RSA	Drosophila	omega[a]	High	0.5636364	0.0158068
RSA	Drosophila	omega[a]	Low	0.5636364	0.0158068

Table 12: Statistics for Disorder

co_factor	species	variable	category	Kendall.tau	p.value
Disorder	Arabidopsis	omega	high	1.0000000	0.0000060
Disorder	Arabidopsis	omega	low	0.9696970	0.0000114
Disorder	Arabidopsis	omega[na]	high	0.9393939	0.0000212
Disorder	Arabidopsis	omega[na]	low	0.9090909	0.0000388
Disorder	Arabidopsis	omega[a]	high	0.6363636	0.0039762
Disorder	Arabidopsis	omega[a]	low	0.4545455	0.0396693
Disorder	Drosophila	omega	high	0.6969697	0.0016086
Disorder	Drosophila	omega	low	0.6363636	0.0039762
Disorder	Drosophila	omega[na]	high	0.3030303	0.1702344
Disorder	Drosophila	omega[na]	low	0.5757576	0.0091672
Disorder	Drosophila	omega[a]	high	0.5151515	0.0197288
Disorder	Drosophila	omega[a]	low	0.2727273	0.2170890
