# Supplementary material for: Strong evidence for the adaptive walk model of gene evolution in Drosophila and Arabidopsis
Source: PLoS Biol. 2022 Sep 13;20(9):e3001775. doi: 10.1371/journal.pbio.3001775 (PMC9470001; doi:10.1371/journal.pbio.3001775)
Supplement: S7 File — (PDF) [file pbio.3001775.s053.pdf]

PiN_PiS analysis

af_moutinho

2022-07-12

This script contains the analysis performed to check whether w_a was correlated to w_na

```
# Libraries
library(plyr)
library(dplyr)
library(data.table)
library(ggplot2)
library(reshape2)
library(doBy)
library(knitr)
library(kableExtra)
#

# theme of the plot
theme.plot <- function(x) {
  theme(axis.title = element_text(face = "bold", color = "black", size=14),
        text = element_text(size=14),
        axis.title.x = element_text(margin = margin(t = 18, r = 10, b = 0, l = 0)),
        axis.title.y = element_text(margin = margin(t = 18, r = 10, b = 0, l = 0)),
        panel.grid.minor=element_blank(),
        panel.grid.major = element_line(colour = "grey", linetype = "dashed", size = 0.2),
        panel.grid.major.y=element_blank(),
        #strip.text.y = element_blank(),
        axis.text.x = element_text(angle = 60, hjust = 1))
}

# change path to where you kep the data
pinpis_age <- read.table(file = "~/Dropbox/SupplementaryData_GeneAge/PiN_PiS/S30_Data.csv",
                         sep = "\t", header = T)

# arranging the table for plotting:
pinpis_age2 <- melt(pinpis_age, id.vars = c("GeneAge", "species"),
      measure.vars = c("dnds", "omegaNA", "omegaA"))

# function to estimate the mean and standard deviation to plot the results with the
# mean of the bootstrap replicates and the 95% confidence interval
fun <- function(x){
  c(mean=mean(x), sd=sd(x))
}

# applying the above function for each value of each estimate
# (dnds, omegaA, omegaNA)
```

```r
tbl.sum <- summaryBy(value ~ variable + GeneAge + species, data=pinpis_age2, FUN = fun)


# to change the estimate name to the respective symbol
tbl.sum$variable <- factor(tbl.sum$variable, levels = c("dnds", "omegaNA", "omegaA"))
levels(tbl.sum$variable) <- c(expression(omega), expression(omega[na]),
                                     expression(omega[a]))

# plotting
plot.age <- ggplot(tbl.sum, aes(x = GeneAge, y = value.mean)) +
  geom_line(col = "black", size = 0.2)+
  geom_ribbon(aes(ymin=value.mean + 1.96*value.sd,
                  ymax=value.mean - 1.96*value.sd), alpha=0.6) +
  geom_point(size=.2)+
  facet_grid(species~variable, scales = "free_x", labeller = label_parsed) +
  ylab("") +
  xlab("Gene Age") +
  #scale_x_sqrt() +
  scale_fill_grey() +
  scale_color_grey() +
  theme_bw() +
  theme.plot()
plot.age
```

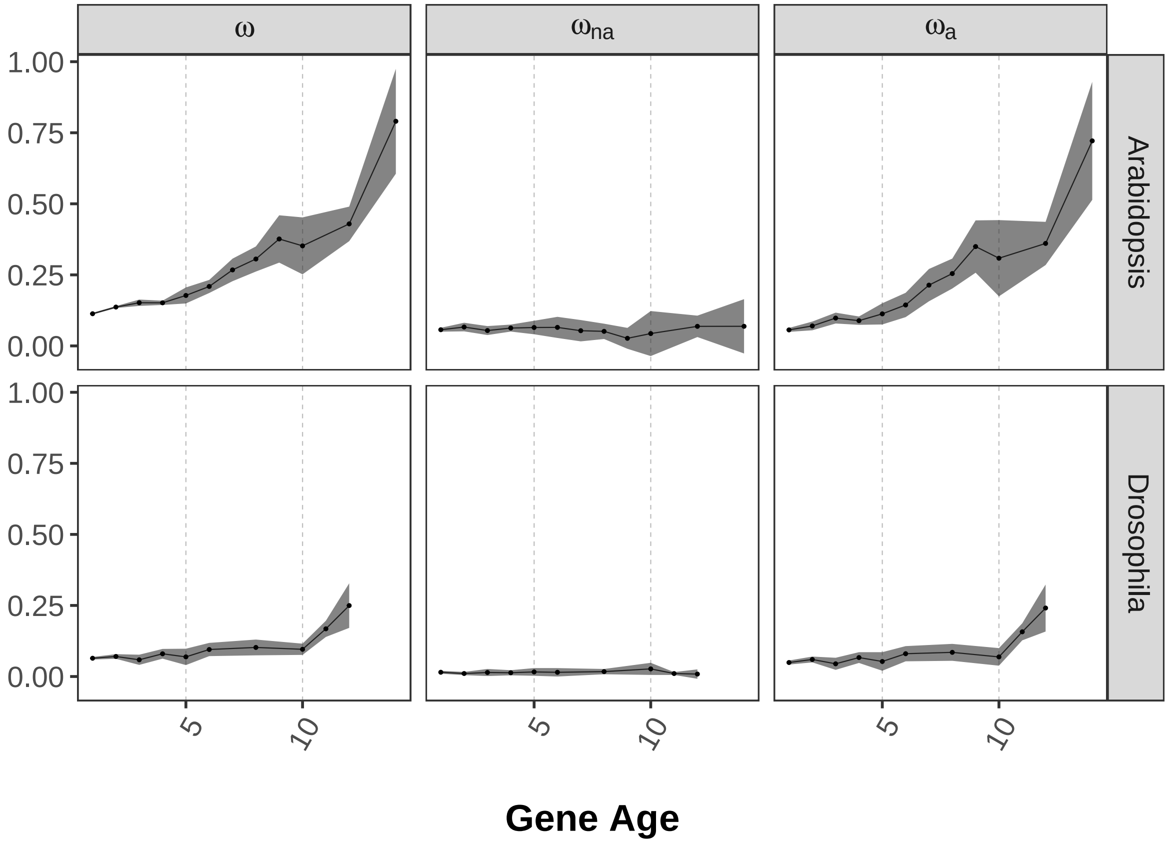

```r
# correlation between age and rates of protein evolution
# ARABIDOPSIS
oA_arab <- subset(tbl.sum, variable == "omega[a]" & species == "Arabidopsis")
corr <- cor.test(oA_arab$GeneAge, oA_arab$value.mean, method = "kendall", exact = F)
Kendall.tau <- corr$estimate
p.value <- corr$p.value
oA_arab_st <- data.frame(Kendall.tau, p.value)
kable(oA_arab_st, caption = "w_a ~ age Arabidopsis")

oNA_arab <- subset(tbl.sum, variable == "omega[na]" & species == "Arabidopsis")
corr <- cor.test(oNA_arab$GeneAge, oNA_arab$value.mean, method = "kendall", exact = F)
Kendall.tau <- corr$estimate
p.value <- corr$p.value
oNA_arab_st <- data.frame(Kendall.tau, p.value)
kable(oNA_arab_st, caption = "w_na ~ age Arabidopsis")

o_arab <- subset(tbl.sum, variable == "omega" & species == "Arabidopsis")
corr <- cor.test(o_arab$GeneAge, o_arab$value.mean, method = "kendall", exact = F)
Kendall.tau <- corr$estimate
p.value <- corr$p.value
o_arab_st <- data.frame(Kendall.tau, p.value)
kable(o_arab_st, caption = "w ~ age Arabidopsis")

# DROSOPHILA
oA_dmel <- subset(tbl.sum, variable == "omega[a]" & species == "Drosophila")
corr <- cor.test(oA_dmel$GeneAge, oA_dmel$value.mean, method = "kendall", exact = F)
Kendall.tau <- corr$estimate
p.value <- corr$p.value
oA_dmel_st <- data.frame(Kendall.tau, p.value)
kable(oA_dmel_st, caption = "w_a ~ age Drosophila")

oNA_dmel <- subset(tbl.sum, variable == "omega[na]" & species == "Drosophila")
corr <- cor.test(oNA_dmel$GeneAge, oNA_dmel$value.mean, method = "kendall", exact = F)
Kendall.tau <- corr$estimate
p.value <- corr$p.value
oNA_dmel_st <- data.frame(Kendall.tau, p.value)
kable(oNA_dmel_st, caption = "w_na ~ age Drosophila")

o_dmel <- subset(tbl.sum, variable == "omega" & species == "Drosophila")
corr <- cor.test(o_dmel$GeneAge, o_dmel$value.mean, method = "kendall", exact = F)
Kendall.tau <- corr$estimate
p.value <- corr$p.value
o_dmel_st <- data.frame(Kendall.tau, p.value)
kable(o_dmel_st, caption = "w ~ age Drosophila")
```
