# Supplementary material for: Strong evidence for the adaptive walk model of gene evolution in Drosophila and Arabidopsis
Source: PLoS Biol. 2022 Sep 13;20(9):e3001775. doi: 10.1371/journal.pbio.3001775 (PMC9470001; doi:10.1371/journal.pbio.3001775)
Supplement: S8 File — (PDF) [file pbio.3001775.s054.pdf]

weighted Z-scores

af_moutinho

20/04/2021

This script includes the data analysis of the combined probabilities for each of co-factors within and across species using the weighted Z-method.

```
setwd("~/Dropbox/SupplementaryData_GeneAge/Stats/")

# Libraries
library(plyr)
library(dplyr)
library(data.table)
library(ggplot2)
library(reshape2)
library(doBy)
library(knitr)
library(kableExtra)
#if (!require("BiocManager", quietly = TRUE))
#    install.packages("BiocManager")
#BiocManager::install("multtest")
#library(multtest)
#install.packages("metap")
library(metap)
#

# calling the tables containing the kendall's correlations between gene age and each of
#the co-factors:
stats_files <- list.files(".", ".csv")

# reading each table:
stats_df <- lapply(stats_files, read.table, header = TRUE)

# re-order column names before combining the tables:
stats_df_order <- lapply(stats_df, function(x) {
  df <- x[,order(names(x))]
})

# combining all tables:
stats.co_factor <- as.data.frame(rbindlist(stats_df_order))
```

In the next chunk of the script, we will estimate the weight each p-value in each co-factor. We used a linear modeling approach with omega_a and omega_na as response variables, and gene age and potential co-factors as explanatory variables and inferred the reciprocal of the squared standard error of the residuals in each model.

```
# calling all output tables
setwd("~/Dropbox/SupplementaryData_GeneAge/Co-factors_continuous/grapes_analysis")

co.factors <- list.files(".", ".csv")

# reading each table into a list
tbl.list <- lapply(co.factors, read.table, header = TRUE)

# combining all tables:
co_factor_boot <- as.data.frame(rbindlist(tbl.list))

# arranging the table:
df.co_factor_boot <- reshape2::melt(co_factor_boot,
                                   id.vars = c("GeneAge", "co_factor", "category", "species"),
                                   measure.vars = c("dnds", "omegaNA", "omegaA"))

# splitting the data.frame to combine with the list of residuals:
df.split <- df.co_factor_boot %>%
  group_by(co_factor, variable, species)

df.list <- group_split(df.split)

# linear regression for each co-factor in each species:
lm.co_factor <- dlply(df.co_factor_boot, .(co_factor, variable, species), lm,
                      formula = value ~ GeneAge + category)

# residuals:
res.lm.co_factor <- llply(lm.co_factor, residuals)

# residuals as data.frame:
df.res <- llply(res.lm.co_factor, as.data.frame)

# mapping residuals to each data point:
list.co_factor_boot <- Map(cbind, df.list, df.res)
df2.co_factor_boot <- as.data.frame(rbindlist(list.co_factor_boot))

# estimating the weight for each co-factor
weight.co_factor <- ddply(df2.co_factor_boot,
                          c("co_factor", "category", "variable", "species"),
                          function(x) {
  weight <- 1/var(x$`X[[i]]`)
  data.frame(weight)
})
```

Now we will combine the weights with the p-values top obtain the combined probabilities:

```
weight.co_factor$variable <- factor(weight.co_factor$variable,
                                   levels = c("dnds", "omegaNA", "omegaA"))
levels(weight.co_factor$variable) <- c("omega", "omega[na]", "omega[a]")

stats.co_factor2 <- merge(stats.co_factor, weight.co_factor,
                          by = c("co_factor", "category", "variable", "species"))
```

Table 1: Combined probabilities for each co-factor in each species

co_factor	variable	species	p.value
Disorder	omega	Arabidopsis	0.0000000
Disorder	omega	Drosophila	0.0000385
Disorder	omega[a]	Arabidopsis	0.0011981
Disorder	omega[a]	Drosophila	0.0417852
Disorder	omega[na]	Arabidopsis	0.0000000
Disorder	omega[na]	Drosophila	0.0058011
Expression	omega	Arabidopsis	0.0015094
Expression	omega	Drosophila	0.0010904
Expression	omega[a]	Arabidopsis	0.1855687
Expression	omega[a]	Drosophila	0.0022389
Expression	omega[na]	Arabidopsis	0.0003712
Expression	omega[na]	Drosophila	0.0016846
Length	omega	Arabidopsis	0.0006458
Length	omega	Drosophila	0.0026432
Length	omega[a]	Arabidopsis	0.1338833
Length	omega[a]	Drosophila	0.0105340
Length	omega[na]	Arabidopsis	0.0016062
Length	omega[na]	Drosophila	0.0052864
RSA	omega	Arabidopsis	0.0000062
RSA	omega	Drosophila	0.0003667
RSA	omega[a]	Arabidopsis	0.0012363
RSA	omega[a]	Drosophila	0.0015467
RSA	omega[na]	Arabidopsis	0.0001406
RSA	omega[na]	Drosophila	0.0007760

```
#### combined probabilities for each co-factor in each species:
weightZ_sp <- ddply(stats.co_factor2,
                    c("co_factor", "variable", "species"),
                    function(x) {
  sumz.df <- sumz(x$p.value, x$weight)
  p.value <- sumz.df$p
  data.frame(p.value)
})

# showing the table:
kable(weightZ_sp, caption = "Combined probabilities for each co-factor in each species")
```

```
#### combined probabilities for each co-factor across species:
weightZ_factor <- ddply(stats.co_factor2,
                        c("co_factor", "variable"),
                        function(x) {
  sumz.df <- sumz(x$p.value, x$weight)
  p.value <- sumz.df$p
  data.frame(p.value)
})

# showing the table:
kable(weightZ_factor, caption = "Combined probabilities for each co-factor across species")
```

Table 2: Combined probabilities for each co-factor across species

co_factor	variable	p.value
Disorder	omega	0.0000000
Disorder	omega[a]	0.0025252
Disorder	omega[na]	0.0000066
Expression	omega	0.0000693
Expression	omega[a]	0.0035345
Expression	omega[na]	0.0000069
Length	omega	0.0001561
Length	omega[a]	0.0079842
Length	omega[na]	0.0000771
RSA	omega	0.0000001
RSA	omega[a]	0.0000137
RSA	omega[na]	0.0000009
