# Supplementary material for: Strong evidence for the adaptive walk model of gene evolution in Drosophila and Arabidopsis
Source: PLoS Biol. 2022 Sep 13;20(9):e3001775. doi: 10.1371/journal.pbio.3001775 (PMC9470001; doi:10.1371/journal.pbio.3001775)
Supplement: S9 File — (PDF) [file pbio.3001775.s055.pdf]

Gene Age Analysis

jy_dutheil

28/05/2022

This script includes extra analyses to correct for intra-category variation of the cofactors.

```
# libraries
library(plyr)
library(kableExtra)
#


d.len <- read.table("~/Dropbox/SupplementaryData_GeneAge/IntraClassVariation/S16_Data.csv",
                    header=T)
d.exp <- read.table("~/Dropbox/SupplementaryData_GeneAge/IntraClassVariation/S17_Data.csv",
                    header=T)
d.dis <- read.table("~/Dropbox/SupplementaryData_GeneAge/IntraClassVariation/S15_Data.csv",
                    header=T)
d.rsa <- read.table("~/Dropbox/SupplementaryData_GeneAge/IntraClassVariation/S14_Data.csv",
                    header=T)
```

Co-factor ~ omegas

```
# RSA
stats_rsa <- ddply(d.rsa, c("species", "category", "variable"), function(x) {
  cor_test <- cor.test(x$value, x$mean.rsa, method = "kendall", exact = F)
  estimate <- cor_test$estimate
  p_value <- cor_test$p.value
  data.frame(estimate, p_value)
})
kable(stats_rsa, caption = "Correlation between RSA and rates of
      protein evolution in each high and low group")
```

```
# Disorder
stats_dis <- ddply(d.dis, c("species", "category", "variable"), function(x) {
  cor_test <- cor.test(x$value, x$mean.dis, method = "kendall", exact = F)
  estimate <- cor_test$estimate
  p_value <- cor_test$p.value
  data.frame(estimate, p_value)
})
kable(stats_dis, caption = "Correlation between protein disorder and rates of
      protein evolution in each high and low group")
```

Table 1: Correlation between RSA and rates of protein evolution in each high and low group

species	category	variable	estimate	p_value
Arabidopsis	High	mean_o	0.6666667	0.0123434
Arabidopsis	High	mean_oA	0.6666667	0.0123434
Arabidopsis	High	mean_oNA	0.6111111	0.0218101
Arabidopsis	Low	mean_o	0.5000000	0.0605689
Arabidopsis	Low	mean_oA	0.2777778	0.2971465
Arabidopsis	Low	mean_oNA	0.5000000	0.0605689
Drosophila	High	mean_o	0.5272727	0.0239677
Drosophila	High	mean_oA	0.4545455	0.0516250
Drosophila	High	mean_oNA	0.7090909	0.0023962
Drosophila	Low	mean_o	0.2363636	0.3115148
Drosophila	Low	mean_oA	0.3818182	0.1020810
Drosophila	Low	mean_oNA	0.0909091	0.6970916

Table 2: Correlation between protein disorder and rates of protein evolution in each high and low group

species	category	variable	estimate	p_value
Arabidopsis	high	mean_o	0.7575758	0.0006066
Arabidopsis	high	mean_oA	0.3939394	0.0746048
Arabidopsis	high	mean_oNA	0.8181818	0.0002131
Arabidopsis	low	mean_o	0.1515152	0.4928862
Arabidopsis	low	mean_oA	-0.1212121	0.5832934
Arabidopsis	low	mean_oNA	0.1515152	0.4928862
Drosophila	high	mean_o	0.3636364	0.0998171
Drosophila	high	mean_oA	0.2424242	0.2725711
Drosophila	high	mean_oNA	0.3939394	0.0746048
Drosophila	low	mean_o	-0.1515152	0.4928862
Drosophila	low	mean_oA	-0.0303030	0.8909161
Drosophila	low	mean_oNA	-0.1515152	0.4928862

Table 3: Correlation between gene length and rates of protein evolution in each high and low group

species	category	variable	estimate	p_value
Arabidopsis	long	mean_o	-0.7333333	0.0387775
Arabidopsis	long	mean_oA	0.2000000	0.5730251
Arabidopsis	long	mean_oNA	-0.6000000	0.0908739
Arabidopsis	short	mean_o	-1.0000000	0.0048322
Arabidopsis	short	mean_oA	-0.6000000	0.0908739
Arabidopsis	short	mean_oNA	-0.8666667	0.0145950
Drosophila	long	mean_o	-0.0666667	0.8509807
Drosophila	long	mean_oA	-0.0666667	0.8509807
Drosophila	long	mean_oNA	-0.3333333	0.3475580
Drosophila	short	mean_o	-0.7333333	0.0387775
Drosophila	short	mean_oA	-0.4666667	0.1884860
Drosophila	short	mean_oNA	-0.8666667	0.0145950

```
# Disorder
stats_length <- ddply(d.len, c("species", "category", "variable"), function(x) {
  cor_test <- cor.test(x$value, x$mean.length, method = "kendall", exact = F)
  estimate <- cor_test$estimate
  p_value <- cor_test$p.value
  data.frame(estimate, p_value)
})
kable(stats_length, caption = "Correlation between gene length and rates of
      protein evolution in each high and low group")
```

```
# Expression
stats_exp <- ddply(d.exp, c("species", "category", "variable"), function(x) {
  cor_test <- cor.test(x$value, x$mean.exp, method = "kendall", exact = F)
  estimate <- cor_test$estimate
  p_value <- cor_test$p.value
  data.frame(estimate, p_value)
})
kable(stats_exp, caption = "Correlation between gene expression and rates of
      protein evolution in each high and low group")
```

Co-factor ~ Gene age

```
co_factor_age <- read.table(file = "~/Dropbox/SupplementaryData_GeneAge/IntraClassVariation/S18_Data.cs
                            sep = "\t", header = T)

cor_df <- ddply(co_factor_age, c("species", "co_factor", "category"), function(x) {
  cor_tbl <- cor.test(x$Clade, x$median_value, method = "kendall", exact = F)
  tau <- cor_tbl$estimate
  p_value <- cor_tbl$p.value
  data.frame(tau, p_value)
})
```

Table 4: Correlation between gene expression and rates of protein evolution in each high and low group

species	category	variable	estimate	p_value
Arabidopsis	High	mean_o	0.0666667	0.8509807
Arabidopsis	High	mean_oA	0.2000000	0.5730251
Arabidopsis	High	mean_oNA	0.0666667	0.8509807
Arabidopsis	Low	mean_o	-0.8666667	0.0145950
Arabidopsis	Low	mean_oA	-0.3333333	0.3475580
Arabidopsis	Low	mean_oNA	-1.0000000	0.0048322
Drosophila	High	mean_o	-0.2000000	0.5730251
Drosophila	High	mean_oA	-0.4666667	0.1884860
Drosophila	High	mean_oNA	-0.0666667	0.8509807
Drosophila	Low	mean_o	-0.6000000	0.0908739
Drosophila	Low	mean_oA	-0.7333333	0.0387775
Drosophila	Low	mean_oNA	-0.7333333	0.0387775

Table 5: Correlation between the co-factor and gene age in each high and low group

species	co_factor	category	tau	p_value
Arabidopsis	Disorder	high	0.5047619	0.0087205
Arabidopsis	Disorder	low	0.2000000	0.2986976
Arabidopsis	Expression	high	-0.0956949	0.6202622
Arabidopsis	Expression	low	-0.8421149	0.0000130
Arabidopsis	Length	long	-0.7904762	0.0000400
Arabidopsis	Length	short	-0.3942490	0.0419555
Arabidopsis	RSA	high	0.5428571	0.0047909
Arabidopsis	RSA	low	0.6380952	0.0009143
Drosophila	Disorder	high	0.4545455	0.0396693
Drosophila	Disorder	low	-0.4242424	0.0548539
Drosophila	Expression	high	-0.2595495	0.2426117
Drosophila	Expression	low	-0.4187179	0.0622294
Drosophila	Length	long	-0.3636364	0.0998171
Drosophila	Length	short	-0.4545455	0.0396693
Drosophila	RSA	high	0.7575758	0.0006066
Drosophila	RSA	low	0.4848485	0.0282123

```
kable(cor_df, caption = "Correlation between the co-factor and gene age in each high
      and low group")
```

The following chunks contain the linear model analyses.

Model fitting

Fit linear models with intra-category co-factor values as explanatory variables, together with some interactions, including:

- Co-factor nested within category (category + category:cofactor)
- Gene age with category
- Gene age with species

Omega

```
m.len.o <- step(lm(value ~ GeneAge + species + GeneAge:species +
                           category + mean.length:category + GeneAge:category,
                   d.len, subset = variable == "mean_o"))
m.exp.o <- step(lm(value ~ GeneAge + species + GeneAge:species +
                           category + mean.exp:category + GeneAge:category,
                   d.exp, subset = variable == "mean_o"))
m.dis.o <- step(lm(value ~ GeneAge + species + GeneAge:species +
                           category + mean.dis:category + GeneAge:category,
                   d.dis, subset = variable == "mean_o"))
m.rsa.o <- step(lm(value ~ GeneAge + species + GeneAge:species +
                           category + mean.rsa:category + GeneAge:category,
                   d.rsa, subset = variable == "mean_o"))
```

OmegaA

```
m.len.oa <- step(lm(value ~ GeneAge + species + GeneAge:species +
                            category + mean.length:category + GeneAge:category,
                    d.len, subset = variable == "mean_oA"))
m.exp.oa <- step(lm(value ~ GeneAge + species + GeneAge:species +
                            category + mean.exp:category + GeneAge:category,
                    d.exp, subset = variable == "mean_oA"))
m.dis.oa <- step(lm(value ~ GeneAge + species + GeneAge:species +
                            category + mean.dis:category + GeneAge:category,
                    d.dis, subset = variable == "mean_oA"))
m.rsa.oa <- step(lm(value ~ GeneAge + species + GeneAge:species +
                            category + mean.rsa:category + GeneAge:category,
                    d.rsa, subset = variable == "mean_oA"))
```

OmegaNA

```
m.len.ona <- step(lm(value ~ GeneAge + species + GeneAge:species +
                             category + mean.length:category + GeneAge:category,
                     d.len, subset = variable == "mean_oNA"))
m.exp.ona <- step(lm(value ~ GeneAge + species + GeneAge:species +
                             category + mean.exp:category + GeneAge:category,
                     d.exp, subset = variable == "mean_oNA"))
m.dis.ona <- step(lm(value ~ GeneAge + species + GeneAge:species +
                             category + mean.dis:category + GeneAge:category,
                     d.dis, subset = variable == "mean_oNA"))
m.rsa.ona <- step(lm(value ~ GeneAge + species + GeneAge:species +
                             category + mean.rsa:category + GeneAge:category,
                     d.rsa, subset = variable == "mean_oNA"))
```

Diagnostic plots

```
getdf <- function(m, var.name) {
  df <- data.frame(Variable = var.name, Fitted = fitted(m), Residuals = residuals(m))
  return(df)
}

getdf.all <- function(m.len, m.exp, m.dis, m.rsa) {
  df.len <- getdf(m.len, "Gene length")
  df.exp <- getdf(m.exp, "Gene expression")
  df.dis <- getdf(m.dis, "Protein disorder")
  df.rsa <- getdf(m.rsa, "Relative solvent accessibility")
  df <- rbind(df.len, df.exp, df.dis, df.rsa)
}

library(ggplot2)
library(ggpubr)
```

```
##
## Attaching package: 'ggpubr'

## The following object is masked from 'package:plyr':
##
##     mutate
```

Omega

Histograms of residuals:

```
dat.o <- getdf.all(m.len.o, m.exp.o, m.dis.o, m.rsa.o)
p.dist.o <- ggplot(dat.o) +
            geom_histogram(aes(x = Residuals, y = ..density..), bins = 20) +
            facet_wrap(~Variable) +
            theme_pubclean()
p.dist.o
```

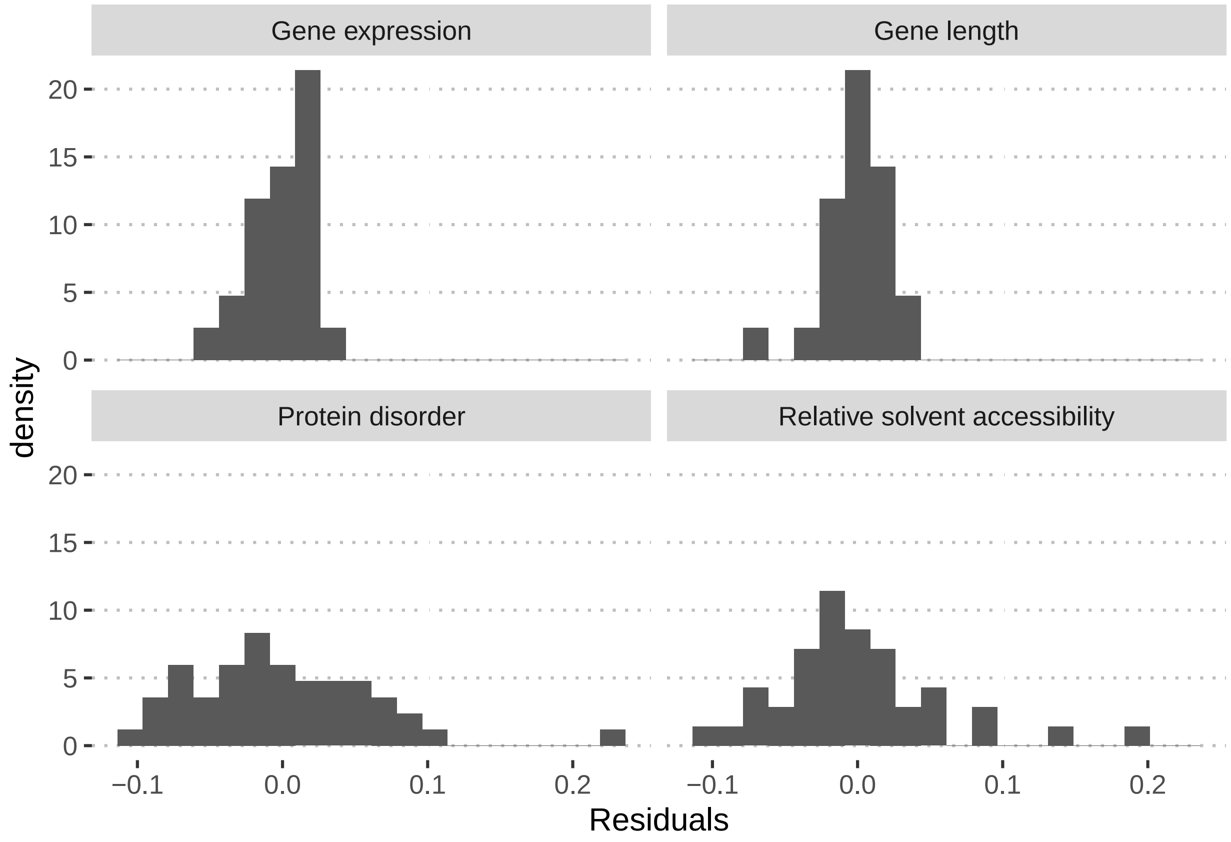

Quantile-quantile plots:

```
p.qq.o <- ggplot(dat.o) +
          geom_qq(aes(sample = Residuals)) +
          geom_qq_line(aes(sample = Residuals), col = "orange") +
          facet_wrap(~Variable) +
          theme_pubclean()
p.qq.o
```

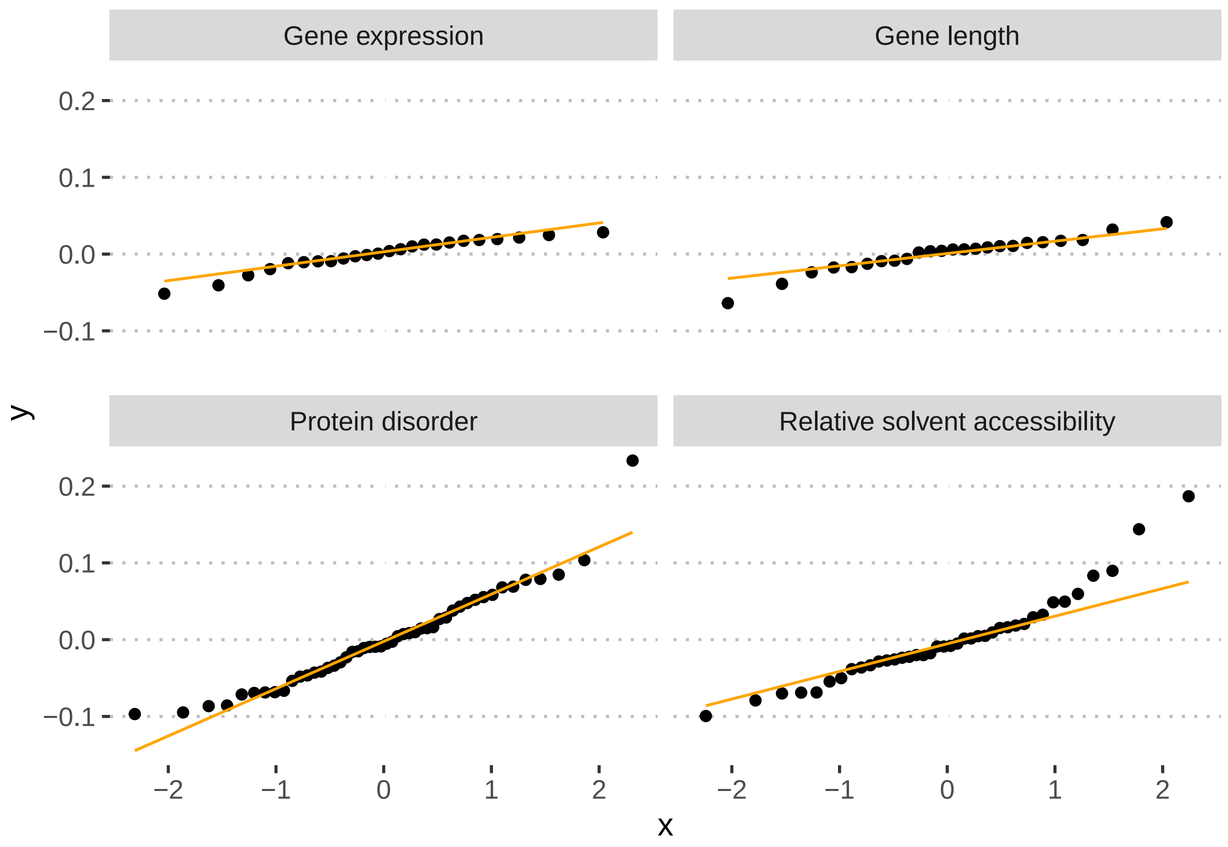

Residuals vs. predicted plots:

```
p.pred.o <- ggplot(dat.o) +
            geom_point(aes(y = Residuals, x = Fitted)) +
            geom_hline(yintercept = 0, col = "orange") +
            facet_wrap(~Variable) +
            theme_pubclean()
p.pred.o
```

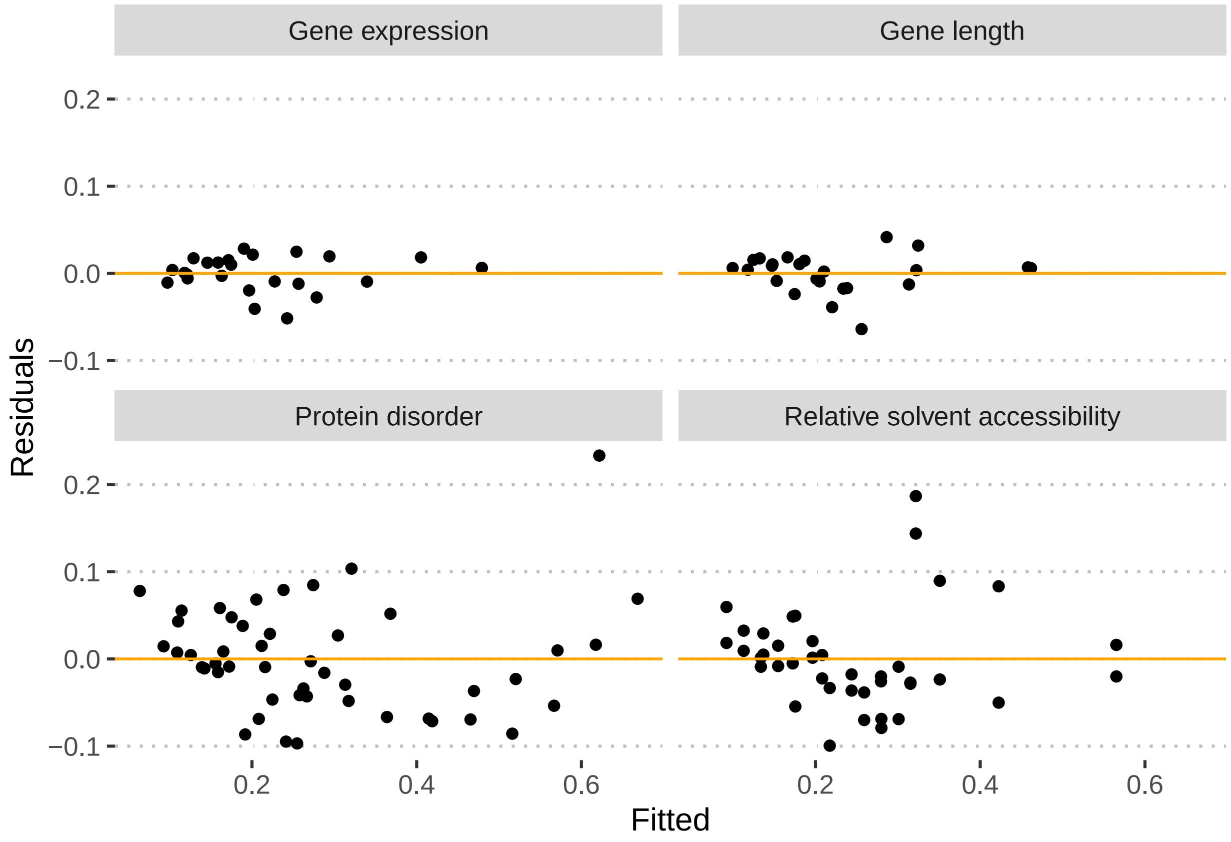

OmegaA

Histograms of residuals:

```
dat.oa <- getdf.all(m.len.oa, m.exp.oa, m.dis.oa, m.rsa.oa)
p.dist.oa <- ggplot(dat.oa) +
             geom_histogram(aes(x = Residuals, y = ..density..), bins = 20) +
             facet_wrap(~Variable) +
             theme_pubclean()
p.dist.oa
```

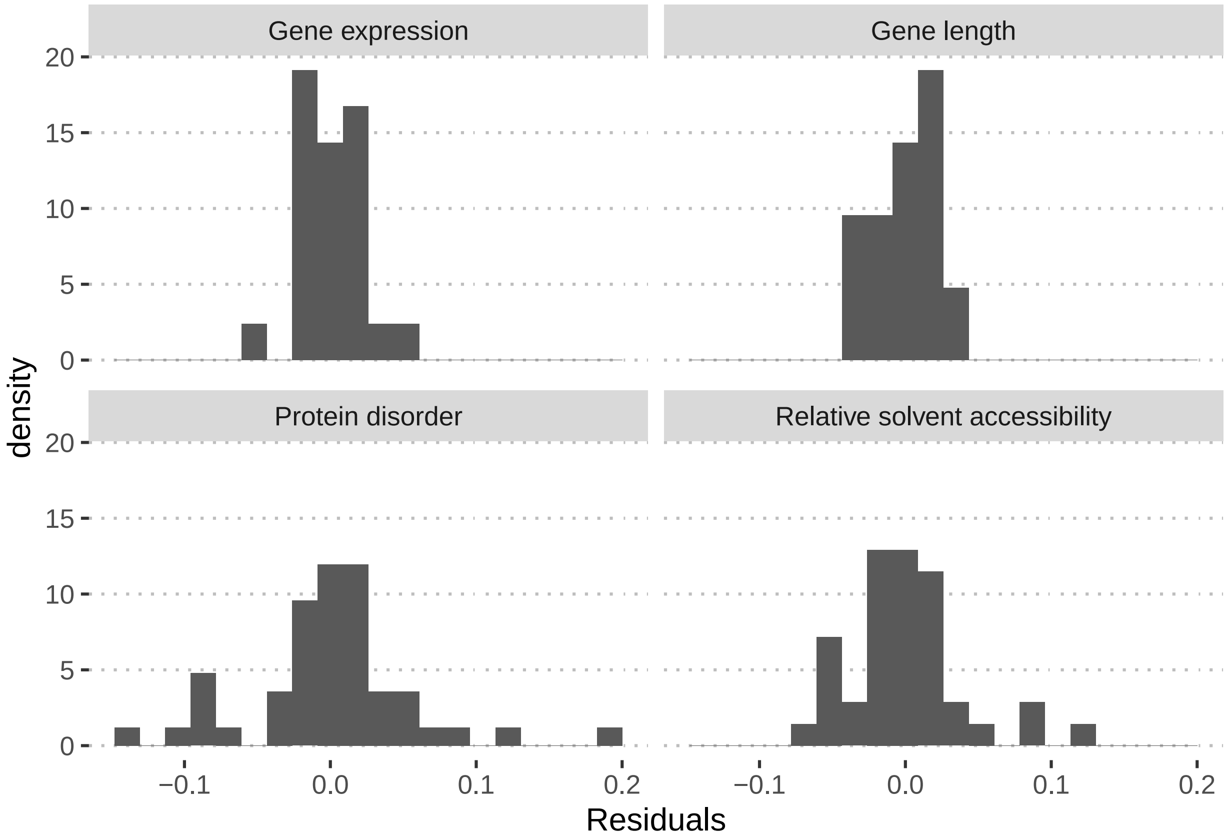

Quantile-quantile plots:

```
p.qq.oa <- ggplot(dat.oa) +
           geom_qq(aes(sample = Residuals)) +
           geom_qq_line(aes(sample = Residuals), col = "orange") +
           facet_wrap(~Variable) +
           theme_pubclean()
p.qq.oa
```

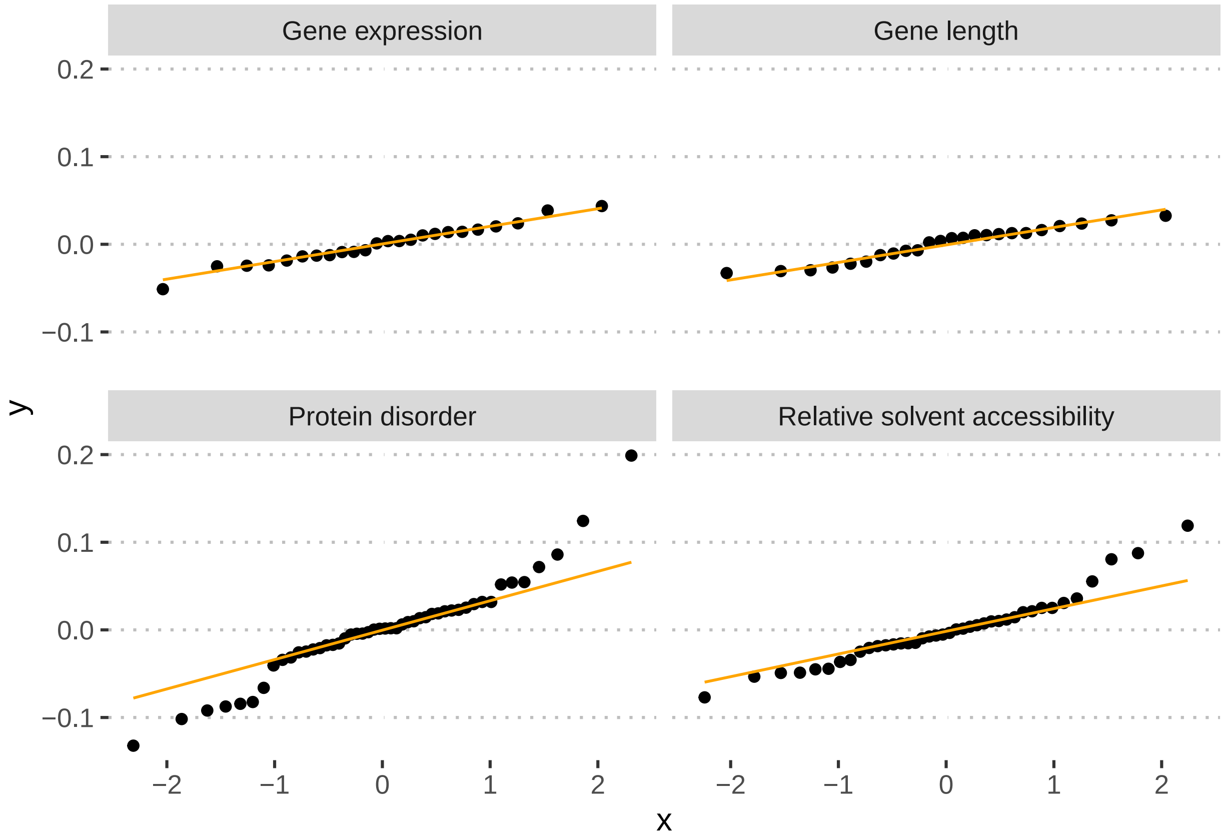

Residuals vs. predicted plots:

```
p.pred.oa <- ggplot(dat.oa) +
             geom_point(aes(y = Residuals, x = Fitted)) +
             geom_hline(yintercept = 0, col = "orange") +
             facet_wrap(~Variable) +
             theme_pubclean()
p.pred.oa
```

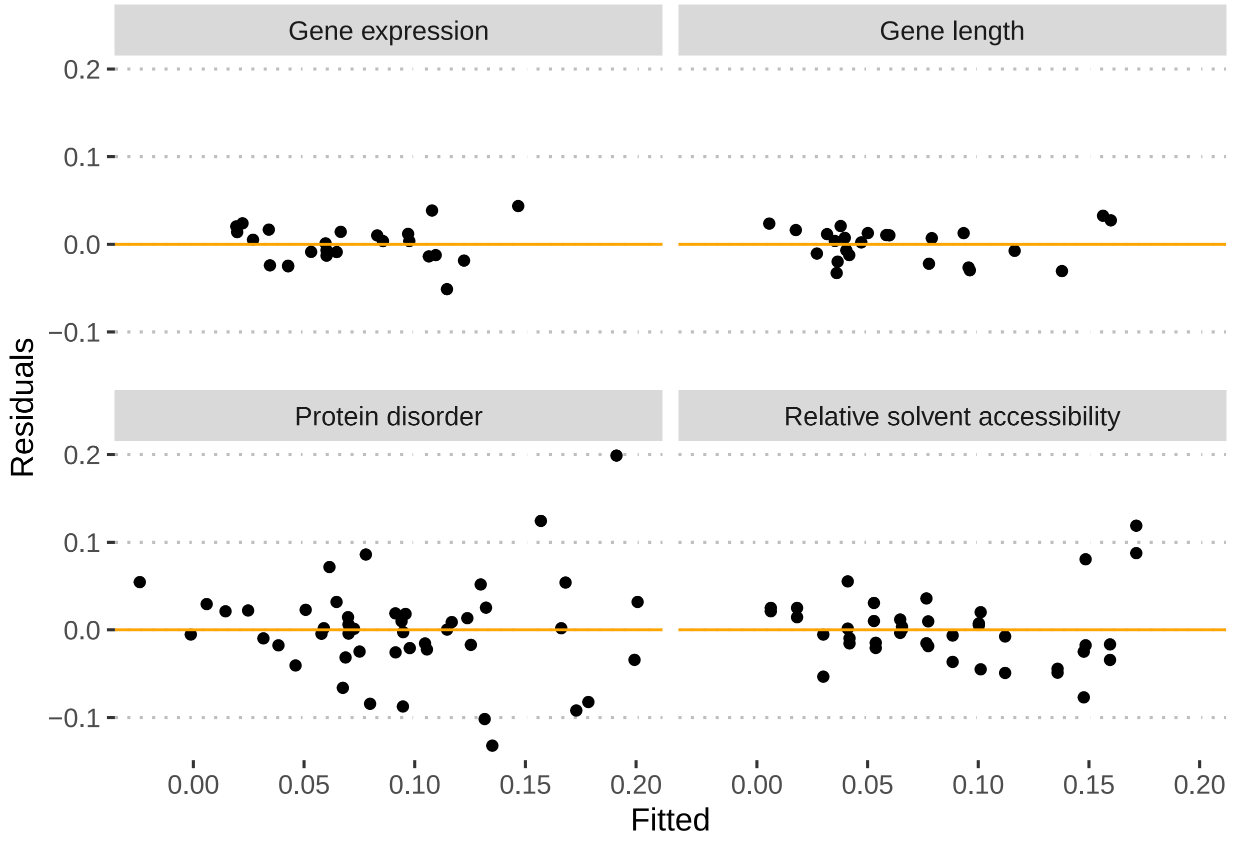

OmegaNA

Histograms of residuals:

```
dat.ona <- getdf.all(m.len.ona, m.exp.ona, m.dis.ona, m.rsa.ona)
p.dist.ona <- ggplot(dat.ona) +
              geom_histogram(aes(x = Residuals, y = ..density..), bins = 20) +
              facet_wrap(~Variable) +
              theme_pubclean()
p.dist.ona
```

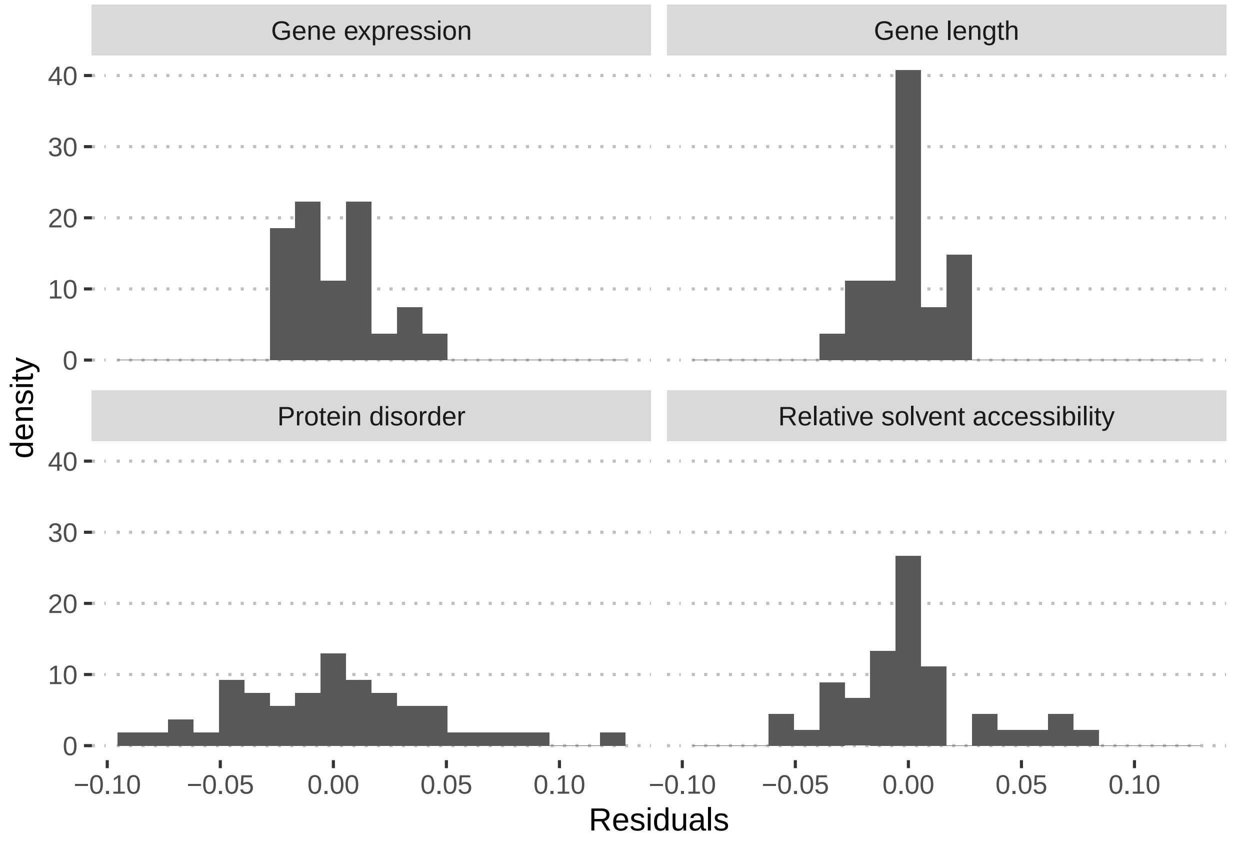

Quantile-quantile plots:

```
p.qq.ona <- ggplot(dat.ona) +
            geom_qq(aes(sample = Residuals)) +
            geom_qq_line(aes(sample = Residuals), col = "orange") +
            facet_wrap(~Variable) +
            theme_pubclean()
p.qq.ona
```

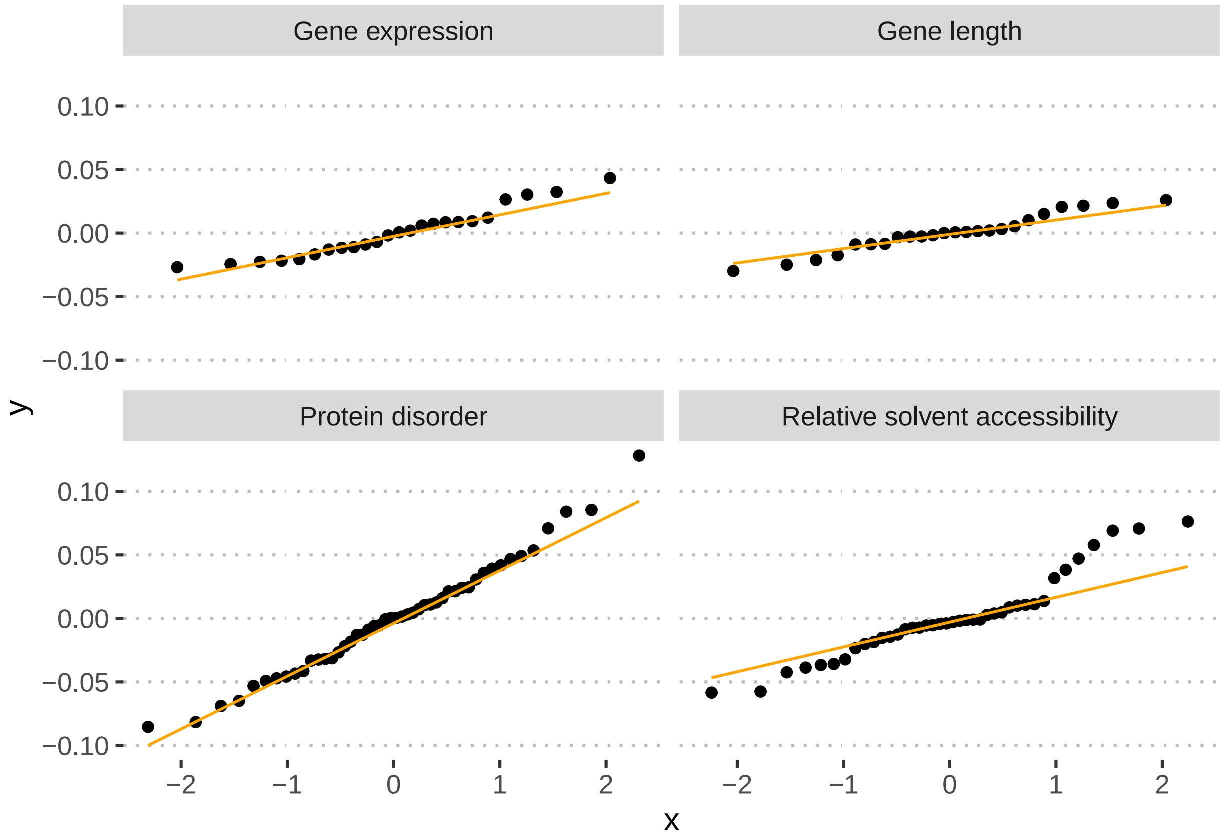

Residuals vs. predicted plots:

```
p.pred.ona <- ggplot(dat.ona) +
              geom_point(aes(y = Residuals, x = Fitted)) +
              geom_hline(yintercept = 0, col = "orange") +
              facet_wrap(~Variable) +
              theme_pubclean()
p.pred.ona
```

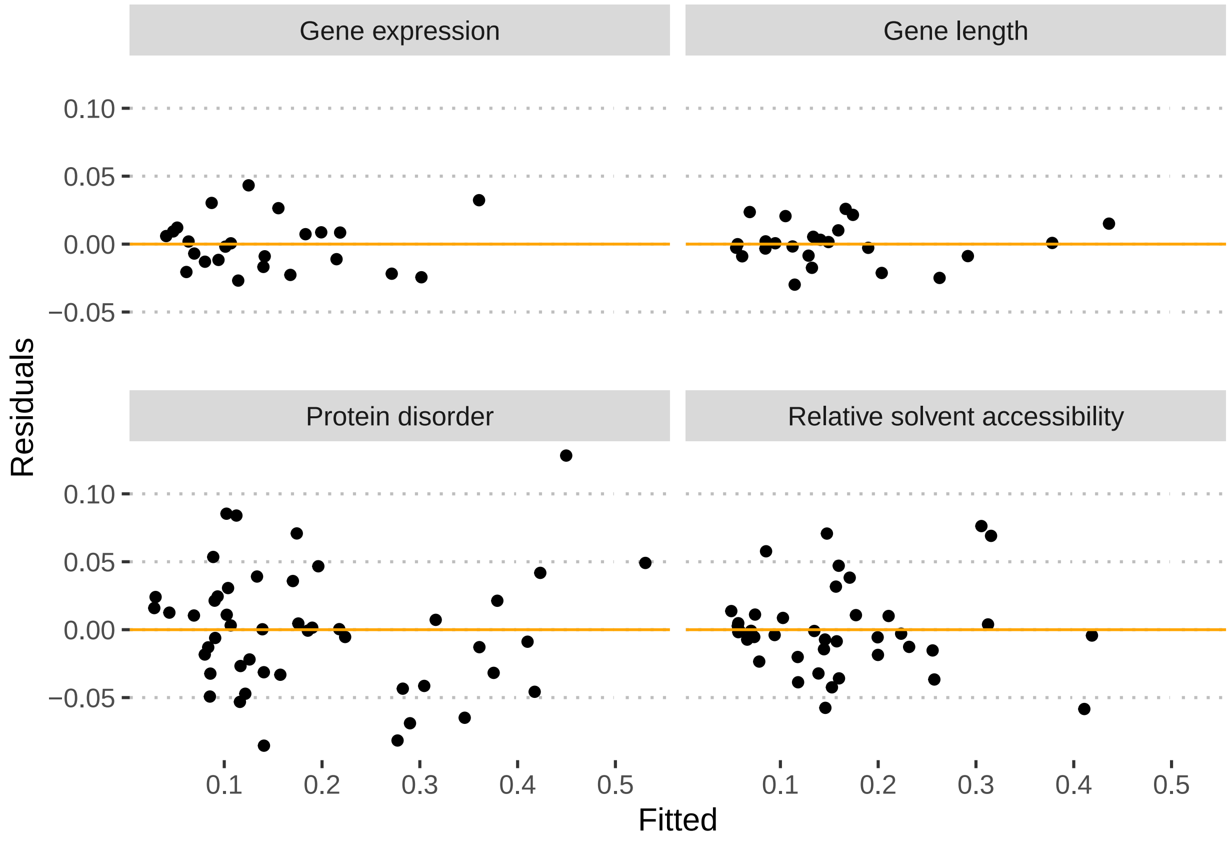

Summarize results

Utilitary functions

```
# From gtools:
stars.pval <- function(p.value) {
  unclass(
    symnum(p.value, corr = FALSE, na = FALSE,
           cutpoints = c(0, 0.001, 0.01, 0.05, 0.1, 1),
           symbols = c("(***)", "(**)", "(*)", "(.)", ""))
  )
}

getres<-function(m, type) {
  s<-summary(m)
  df<-as.data.frame(s$coefficients[,c(1,4)])
  df$Pvalue <- stars.pval(df[,2])
  colnames(df) <- paste(colnames(df), type, sep = "")
  df$Variable <- row.names(df)
  row.names(df) <- NULL
  df <- df[,c(4, 1, 2, 3)]
  return(df)
}
```

```r
getres.all<-function(m.o, m.oa, m.ona, var.name) {
  df.o <- getres(m.o, ".omega")
  df.oa <- getres(m.oa, ".omegaA")
  df.ona <- getres(m.ona, ".omegaNA")
  df <- merge(df.o, df.oa, by = "Variable", all = TRUE, sort = FALSE)
  df <- merge(df, df.ona, by = "Variable", all = TRUE, sort = FALSE)
  df$Cofactor <- var.name
  return(df)
}
```

Gather results

```r
df.len <- getres.all(m.len.o, m.len.oa, m.len.ona, "Gene length")
df.exp <- getres.all(m.exp.o, m.exp.oa, m.exp.ona, "Gene expression")
df.dis <- getres.all(m.dis.o, m.dis.oa, m.dis.ona, "Protein disorder")
df.rsa <- getres.all(m.rsa.o, m.rsa.oa, m.rsa.ona, "Relative solvent accessibility")
df <- rbind(df.len, df.exp, df.dis, df.rsa)
df <- df[, c(11, 1:10)]
write.csv(df, "ModelResults.csv")
```
