# Supplementary material for: Strong evidence for the adaptive walk model of gene evolution in Drosophila and Arabidopsis
Source: PLoS Biol. 2022 Sep 13;20(9):e3001775. doi: 10.1371/journal.pbio.3001775 (PMC9470001; doi:10.1371/journal.pbio.3001775)
Supplement: Attachment — Submitted filename: Response_reviewers-v5.pdf [file pbio.3001775.s056.pdf]

Response to the reviewers

Reviewer #1:

The manuscript by Moutinho et al. seeks to examine molecular adaptation and adaptive walks in natural populations of Drosophila and Arabidopsis. The adaptive walk model predicts that the initial steps of adaptation will consist primarily of many mutations of larger effects. Then, as the population gets closer to the fitness optimum, more small-effect mutations will predominate. Testing this prediction has been challenging for a number of reasons, some of which are related to the myriad of genomic confounders of genomic studies in natural populations. In this study, the authors hypothesize that newly arisen genes will be in the earlier stages of adaptive walks, experiencing more adaptive mutations and larger effect mutations while older genes will have smaller effect mutations. Indeed, by analyzing patterns of polymorphism and divergence in multiple species, the authors find support for this model. Specifically, they find that younger genes tend to have a higher rate of adaptive evolution than older genes. Further, substitutions in younger genes appear to result in changes that are less chemically similar to substitutions seen in older genes.

Overall, I found this paper to be a very creative and elegant study of an important fundamental question in evolutionary biology. The methods appear generally robust and the manuscript is clearly written. However, I have a number of concerns and suggestions on how to improve the manuscript.

We thank the reviewer for the positive comments about the manuscript.

Major comments:

1. Figure 2 and related analyses of confounders: I appreciate that the authors have attempted control for many of the confounders of other factors like gene expression level, protein length, and the amount of intrinsic disorder among proteins that can affect the rate of evolution and rate of adaptation. Indeed, the correlation between rate of adaptation and gene age seems to persist, even when only analyzing genes that are matched on the confounder. However, the analyses seemed to only control for a single confounder at a time. Do associations between the rate of adaptation and the other confounders persist when controlling for a single confounder. Put another way, does the association between rate of adaptation and protein length persist when controlling for expression level, for example? I wonder whether the main effect of gene age on evolutionary rate would still persist when somehow controlling for multiple confounders simultaneously? I realize that the authors indicate they cannot stratify into additional groups because they will not have enough genes in each group for a meaningful comparison. However, might some of the confounders also be correlated with each other? If so, might it possible to control for the joint effects of the confounder?

A1: *We thank the reviewer for their suggestion. To further assess the joint effect of the confounders, we have extended the MK regression analysis* [1]. *The MK regression extends the MK test with a generalized linear model and estimates the direct and indirect effects on the rate of adaptive evolution at the site level by analysing multiple factors simultaneously. The possibility to jointly assess multiple factors, however, comes at the cost of not modelling the distribution of fitness effects. To correct for this, variants segregating at low frequencies (below 50%) must be removed to control for slightly deleterious mutations, which can bias estimates of* ω_a. *We think that the MK analysis is complementary to our stratification approach. It showed a strong effect of gene age (Table S3) even after removing substantial amounts of data and jointly accounting for all confounding factors (lines 191-201).*

2. Related to point 1 above and controlling for confounders—In figure 2 the authors report diving the genes into 2 equal groups based on the confounder and then testing for the main effect within each group. I'm not sure whether this is sufficient to adequately remove the effects of the confounder. For example, in Figure 2b, do genes in the "high expression" group still show a correlation between gene expression and rates of evolution? I worry that there will still be some variability in gene expression

within each of the two large groups that could account for some of the association between the gene age and the rate of evolution. My comment holds for the other confounders as well.

A2: *This is a relevant concern, which we have addressed in the revised manuscript with three tests: (1) the correlation between ω_a and the mean value of each co-factor in each age class within the "high" and "low" groups; (2) the correlation between the co-factor and gene age in each "high" and "low" group; and (3) a linear model where ω_a is the response value, and gene age, category, species, and the within category cofactor values are explanatory variables. While we do observe a general correlation of the co-factor value with gene age within each group, we found that nearly all correlations between ω_a and the co-factor are non-significant (Table S1). Moreover, the linear models showed that gene age was highly significant in all cases but gene length, which is only significant in Drosophila (Table S2). We further note that this concern is also addressed by the MK regression, which models individual sites without any categorization (see A1). We believe that these additional analyses consistently support an effect of gene age that is independent of the effect of the co-factors (lines 178-201).*

3. Lines 212-241: The authors correctly are concerned that young and old genes may have different biological functions. If the young and old genes have different biological function, they may have different distributions of fitness effects and could have different rates of adaptation because of the differences in gene function, rather than the genes only differing in terms of where they are in adaptative walks. The authors attempt to control for this confounder using GO analyses. However, I'm not sure their control is adequate. Might it be possible to match the sets of "younger" and "older" genes based on having a similar set of GO terms and then testing whether the rates of adaptation differ between the younger and older genes? This matching on GO terms might better ensure that genes being compared have similar functional properties.

A3: *We agree with the reviewer that the suggested analysis would be a more powerful attempt to correct the effect of gene function. Unfortunately, the distribution of GO terms is highly unbalanced between age categories, making the matching unfeasible. Therefore, we used the alternative approach of assessing the effect of gene age within GO categories, albeit restricting the analysis to the most abundant GO terms and pooling some age classes, independently for each category.*

4. Lines 493-508: I like the analysis of amino acid exchangeability and gene age. However, I had a really hard time understanding how "G_a" and "G_na" were estimated in these analyses. I found the explanation in the Methods section to be rather vague and unclear, For example, "f_AGT" wasn't defined. Related, how well does this method using the SFS to estimate the rate of adaptation actually work for estimating G_a? Given the importance of these results for the overall conclusions of the paper, I think more description is required as well as some way of showing that the methods work. I tried looking at Bergman and Eyre-Walker [106], but that didn't help clarify things as much as needed.

A4: *We have now rephrased the description of this test with the goal to improve its clarity. We have defined all terms and have rephrased the statistics $\bar{G}_a$ and $\bar{G}_{na}$; now defined as the average Grantham's distance amongst adaptive and non-adaptive substitutions. We have now clarified this analysis in the Methods section (lines 523-550).*

Minor comments:

1. Line 76: Also, experimental studies are limited to only certain organisms.

A5: *We added the following sentence to the text (lines 77-78): "Experimental studies, however, can only assess patterns of adaptation at relatively short time scales in artificial environments and are limited to certain organisms."*

2. Line 204: The use of the word "prevailed" here seems a little awkward. Maybe say "remained" or

"persisted" instead.

***A6:** We changed the wording as suggested (line 226).*

3. It would be informative to include the P-values somehow on each plot in Figure 2.

***A7:** We have added the significance levels to each plot in Figure 2.*

4. Figure S7: P_a and P_na are listed in the caption but G_a and G_na are listed in the figure labels. Are these the same thing? Please clarify. I'm also confused by the point of this figure more generally. Please explain more.

***A8:** We thank the reviewer for noting this annotation error. The revised manuscript now includes a corrected caption. As we observed a strong correlation between the Grantham distance and gene age, we aimed at assessing whether this effect was due to adaptive or non-adaptive substitutions. This figure shows the correlation between the average Grantham's distance amongst adaptive (Ga) and non-adaptive (Gna) substitutions and gene age. Our findings suggest that, in Arabidopsis, we have a larger effect of non-adaptive substitutions, whereas, in Drosophila, substitutions with a larger biochemical distance are mostly adaptive. We further clarified this analysis in the text (lines 278-288).*

Reviewer #2:

In this manuscript the authors test Fisher's geometric model and the idea of the adaptive walk where populations further away from the fitness optimum are more likely to fix beneficial mutations of larger effect first and of smaller effects later. They do so by testing this hypothesis in old vs new genes in Arabidopsis and Drosophila. Overall, I find the idea interesting and definitely worth testing. However, I'm not convinced of the results. Here are some specific and detailed comments:

We thank the reviewer for their comments. Thanks to the reviewers' comments and suggestions, we have improved our manuscript and conducted additional checks to strengthen the evidence supporting our conclusions.

Major Comments:
- I'm not entirely convinced of the results, which seem a bit over-sold throughout the manuscript without the explicit listing of caveats.
a) For instance, from reading the abstract it appears that you have really estimated the selection coefficients of the adaptive substitutions; however, you have used only a proxy for that.

***A9:** Our manuscript reports two types of results: estimates of the rates of adaptive non-synonymous substitutions in various categories of genes, and measures of the average effects of such mutations. While the latter uses a proxy for fitness effects (Grantham's distance), the former is based on population genetic modelling that includes a distribution of fitness effects, which is fitted to the data. The sentence in the abstract "we fitted models of the distribution of fitness effects" referred to this aspect. We rephrased this part of the abstract to improve its clarity:*
"While controlling for these factors, we used population genomic datasets of Arabidopsis and Drosophila and estimated the rate of adaptive substitutions across genes from different phylostrata. We found that a gene's evolutionary age significantly impacts the molecular rate of adaptation. Moreover, we observed that substitutions in young genes tend to have larger physicochemical effects." (lines 47-51).

b) Table S1 does show a positive correlation with gene age but there's a much stronger correlation with other factors like gene length and RSA.

A10: *In this study, we were not looking into whether gene age had the strongest effect, but rather whether its effect persisted after controlling for multiple confounding factors. We have now included an analysis estimating the relative contribution of each variable to the regression model and discuss its results in the main text (lines 202-213). We show that, while gene age does not exhibit the largest effect size, it has a significant effect on the rate of adaptive and non-adaptive evolution that is independent of the effect of the other factors, consistent with an adaptive walk.*

c) I'm also not convinced of the positive correlations between w_a and gene age, especially after you account for gene length and disorder (Figure 2a Arabidopsis; Figure 2d both). They appear extremely weak, unfortunately.
I would urge the authors to tone down the interpretation of their observations and discuss the caveats more.

A11: *Indeed, the correlation is weaker when controlling for some factors. This is expected, as the stratification approach that we employed implies a reduction of statistical power because of data subsetting. We discuss these caveats in the discussion section, where we consider the differences between species (lines 301-334). Particularly, we found that in Arabidopsis, we seem to have a stronger effect of purifying selection rather than positive selection. In the revised manuscript, we extended our MK regression analysis, which allows us to assess all confounding factors simultaneously and consistently, without a need for taking subsets of the data. We found that, when controlling for all confounding factors simultaneously, the effect of gene age is still significant, suggesting that the age of a gene independently impacts the rate of adaptive evolution (see also our response to reviewer #1, A1).*

-It would be a good idea to more thoroughly describe the idea behind using Grantham's distance in the Results section. It's not exactly a standard test and is not used very widely.

A12: *We have now clarified this analysis (see our response to reviewer #1, A8).*

-In this particular study, I'm not really sure that using Grantham's distance is the best way to show that fitness effects of substitutions are large for young genes. If young genes are more likely to be disordered, they might also be less likely to have strong fitness effects when the physicochemical distances are larger. So this appears to be a confounding factor here. Have you looked at the correlations between Grantham's distance and gene age while accounting for the confounding factors? If I missed it, I apologize!

A13: *As discussed in A9, our aim in using Grantham's distance was to look at the effect size of the mutation and not directly at its strength of selection. It is difficult to estimate the strength of selection acting upon advantageous mutations, so we have elected to use physicochemical measures that are likely to be correlated to the mean strength of selection. We have now made it clear that we are considering physicochemical distances rather than fitness (see A9).*

Besides, by looking at the correlation between gene age and Grantham's distances after accounting for the effect of protein disorder, we observed that it is still significant (see below Figure R1). Hence, the correlation between gene age and Grantham's distances does not depend on protein intrinsic disorder. In order to not lengthen the manuscript, we have decided not to include this figure in the text, but it can be reproduced from the scripts provided as supplementary material (file S4 in supplementary data online).

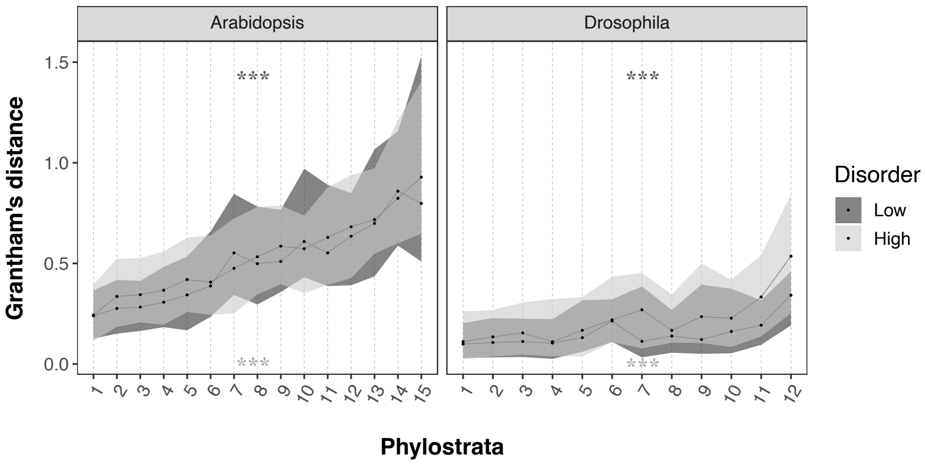

Figure R1. *Relationship between gene age and Grantham's distance for A. thaliana and D. melanogaster. This analysis was performed by categorizing gene age according to the clades defined in Figure 1a. For each clade and each disorder group, the median value of Grantham's distances is depicted with the black dot. The shaded area represents the Grantham's distances values within the 1st and 3rd quartile. Significance levels are shown for the correlation between gene age and Grantham's distances in each protein disorder category (*P < 0.05; **P < 0.01; ***P < 0.001).*

Minor Comments:

-Lines 131-134 -> perhaps elaborate on the methods used here?

A13: *We have updated the Results section and this part has been removed. Furthermore, we now have clarified the methods used in the first and second sections of the Results.*

-line 139 -> briefly explain how you got gene age.

A14: *This is now included in the text (lines 136-137): "Gene age data were obtained from published data sets [28,33]."*

Reviewer #3:
[identifies himself as Nicolas Lartillot]

This manuscript explores the relation between gene age (such as determined by phylostratigraphy) and the rate and patterns of adaptive and non adaptive evolution (such as measured by MacDonald Kreitman approaches), in Drosophila and Arabidopsis. The main results are as follows:
- younger genes undergo higher rates of molecular evolution, both adaptive and non adaptive; this correlation appears to be robust when controlling for multiple confounding factors;
- younger genes tend to make more radical amino-acid changes, and this, both for adaptive and non-adaptive events.
These results are interpreted in terms of an adaptive walk model of gene adaptation and subsequent molecular evolution.

This is a very interesting article. The results presented in it appear to be robust, and the evolutionary hypotheses are stimulating. The article is also well written: very clear, it is a pleasure to read it. I highly recommend it for publication.

We thank the reviewer for his enthusiastic feedback on our work.

I would just have two main comments, one concerning the statistical details of the methods, and

another one concerning the interpretation of the results. Then, there are a few very minor comments below at the end.

1. Statistical analyses: strength of evidence versus strength of correlation.

As a general rule, it is useful to discriminate between strength of evidence, on one side, and strength of correlation on the other side. In a typical statistical analysis, information is given separately about the two aspects (typically, a p-value for the strength of evidence, and then a correlation coefficient for the strength of correlation). Strength of evidence scales with sample size (p-values converge to 0 for larger data samples), whereas strength of correlation is a population-level property (correlation coefficients don't systematically increase, they are just more accurately estimated, with larger data samples).

In the present case, however, it seems to me that there is a latent confusion between these two aspects. For instance, the kendall's tau values presented in table 1 are correlation coefficients, so at first sight, one would be tempted to interpret them as a measure of the strength of correlation between gene age and, e.g. the rate of adaptive evolution. Sentences such as 'the effect of gene age prevailed for all estimates in the two species (omega: tau = 0.929, p =1.30e-03)' implicitly convey this message that tau is giving a measure of the strength of correlation, while p is giving a measure of the strength of evidence for this effect size.

However, these tau values are very close to 1, some of them are even essentially equal to 1. This surprised me at first sight. But then I realized that, given the design of the experiment, this is just a consequence of sample size: these tau values measure the correlation between bin median age and bin *mean* effect. The mean effect of a given bin is an average over many genes, and thus much of the variance in gene adaptive or non-adaptive rate has already been factored out at that step. Ultimately, these tau values should all be equal to 1 for very large numbers of genes per bin, which suggests that they should certainly not be taken as a measure of the intrinsic, population-level, strength of the correlation between gene age and other quantities such as omega_a. They cannot be intrinsic, since they scale with sample size (or, here, with bin size).

Similarly, the confidence intervals displayed on the figures such as figure 1 are obtained by bootstrapping the genes within each bin. But then this means that their width should shrink as 1/sqrt(n), with n the number of genes per bin. So, again, the confidence regions displayed on the figures represent the strength of evidence for the correlation patterns, but not directly the strength of the correlation itself: again, they scale with sample size.

Conversely, I don't see any statistical quantity across the article, whether on the text, tables, or figures, that could be taken as a measure of the intrinsic strength of correlation at the gene level: basically, a measure of how much the age of a randomly chosen gene can predict the evolutionary/adaptive rate of this gene (typically, how much of the variance is explained by the covariate). This is missing a lot, I think.

In this respect, there is a point in the discussion:
"we observe that young genes present a 25-fold higher rate of adaptation than older genes in Drosophila species and around 30-fold higher in Arabidopsis. "
-> this sounds like a measure of effect size; but again, it is only about the mean for a given age class; also it is related to the slope of the regression of omega versus gene age, but not to the strength of the correlation. If we take the linear regression case, which is simpler to understand, you can have a steep relation between Y and X (i.e. mean[Y|X=x_highest] can be much higher than mean[Y|X=x_lowest], while still having a weak correlation (var[Y | X] still large, compared to the variance of mean[Y|X] over X).

And thus, I was wondering if the Authors could think a bit about this point and clarify and, perhaps even, enrich, this aspect of their statistical analyses.

- clarify: clearly say, in the main text, or figures, or table legends, whenever a correlation coeff or a confidence interval scales with sample size (possibly indirectly, i.e. by playing with the number of bins, discretization scheme, etc). perhaps expand a bit on the fact that all this does not really measure the intrinsic strength of the correlation between gene age and gene rate of evolution;
- enrich: if possible, give some meaningful measure of the intrinsic gene-level strength of correlation between gene age and molecular evolutionary rates, or proportion of variance explained.

I think one way to estimate the proportion of variance explained would be to compare the bootstrap variance obtained in the experiments done here, with the same bootstrap variance but in a control experiment where genes have been randomly reshuffled across bins, while keeping the same number of genes per bin (thus erasing all information about gene age). If gene age is a good predictor, then the first variance should be smaller than the second, and 1 - v1/v2 should be a measure of the percentage of variance explained by gene age.

Another quicker way would be as follows: assuming that omega_a (or omega_na) is an additive property, such that the mean omega_a for a set of genes is just, conceptually, the mean of the n gene-specific omega_a's, then it would make sense to just inflate the bootstrap variance estimates by n; this should give a rough estimate of the intra-class (i.e. gene-level) true variance of the effect being measured. And then it is relatively simple to compare this intra-class variance (averaged over all bins) with the inter-class variance of the means. One problem is that genes are of varying length, so one should perhaps inflate the variances, not by the true but by the effective number of genes: n_eff = (sum_i L_i)^2 / (sum_i L_i^2), where L_i is the length of gene i.

There are probably better approaches than those suggested here. Of note, I don't think it is a problem if gene age turns out to explain a small proportion of the total variance. Molecular evolutionary patterns are always rather subtle, so one should not consider this possible outcome as a weakness in itself. But it is just that it would be nice to have at least some hint, some quantitative evaluation, or some discussion, about this in the manuscript. In any case, it is important to clarify and to avoid any misunderstanding about the meaning of the statistical measures of strength of signal that are presented.

A15: *We thank the reviewer for pointing this out. We agree that the correlation coefficients do not allow a proper quantification of the size effects, as they do not account for the intra-category variance. We have clarified this in Table 1 and in the main text (lines 202-205):*

"Lastly, we aimed at assessing the effect size of gene age on ω_a relative to other factors. Because correlation coefficients were computed from values averaged over multiple genes and genes were categorized differently for each analysis, the comparison of correlation coefficients does not provide a reliable estimate of relative effect sizes."

In order to address this issue, together with the other concern of jointly assessing all confounding factors, we added a new set of analyses based on the recently developed MK regression. We further implemented a pseudo-R2 approach. Pseudo-R2 is interpretable in terms of explained variance in the case of ordinary least-squares. In the general case, it is a measure of model fit, which allows us to assess the relative contribution of each factor.
Pseudo-R^2 values were estimated by comparing the log-likelihood (obtained from the MK regression) of the full model (all factors included) with that from each reduced model (each factor removed) using the following equation [2]*:*

$$R2 = 1 - (exp\,(-\frac{2}{N} * (ln_{full} - ln_{reduced}))$$

where N represents the number of sites analysed with the MK regression, ln_{full} represents the log-likelihood of the model with all factors included, and $ln_{reduced}$ represents the log-likelihood of the model excluding each factor (e.g., a model including all variables except gene age). Importantly, each factor contribution is here assessed at the nucleotide site level, not per gene, as this is the unit used in

the MK regression, resulting in overall very low R2 values. These values, however, allowed us to compute the relative contribution of each factor. We found that, while significant, gene age contributes comparatively little to the variation of ω_a, compared to other confounding factors. We discuss these new results in the discussion section of the revised manuscript.

2. Interpretation of the results.

The interpretation of the results in terms of adaptive walk is definitely an interesting one. However, I could think of other interpretations. For instance:

Less constrained genes are more easily lost: they can more easily accommodate mis-sense mutations, so they can probably also more easily accommodate non-sense mutations. Therefore, on average, less constrained genes are younger (because the older ones have been lost). In addition, since they accept a larger number of mutations that are otherwise not too deleterious for the folding and for the primary function of the protein, less constrained genes also represent a bigger mutation target for serendipitous adaptations. So less constrained proteins show a higher rate of adaptive evolution. And thus, younger genes, which are enriched in less constrained genes, show a higher rate of both non-adaptive and adaptive evolution. Of note, this mutational target size argument also explains why omega_na and omega_a are correlated, irrespective of gene age. Also, note that the alternative interpretation just suggested is based on a stationary scenario. In contrast, the adaptive walk idea is fundamentally non-stationary.

To be clear: this is not to dismiss the interpretation proposed by the Authors. But it is just that I found the manuscript perhaps a bit too exclusively oriented toward one single 'story' for explaining the pattern, and this at the cost of a broader - and richer - discussion about what could be responsible for these observations. Perhaps, in their discussion, the Authors could give some hints as to other possible interpretations; also, they could give some suggestions as to how one could, in the future, discriminate between these alternative explanations. For instance, since the interpretation proposed by the Author is deeply committed to a non-stationary pattern, it should be detectable by estimating variation in dN/dS across a phylogenetic tree: analyses along those lines would definitely be an interesting perspective, as a way to discriminate between stationary or non-stationary explanations of these findings more generally.

A16: *We thank the reviewer for these very interesting suggestions, we have now added a broader discussion on what could explain the patterns we observe (lines 389-401). Intriguingly, we found the correlation between gene age and ω_a remains significant after removing the variation in pn/ps. pn/ps is a measure of the segregating neutral and slightly deleterious variation in the population. If ω_a was dependent upon ω_{na}, we would expect that the correlation between gene age and ω_a would disappear if we removed the correlation between ω_{na} and gene age. However, this is not what we find. Although it does not discard the hypothesis of a stationary mode of adaptation, it does support the adaptive walk model.*

Minor points:

The overall effect of gene age on omega_a and omega_na in each co-factor was assessed by...
-> not clear what is meant by overall effect in each co-factor. perhaps rephrase?

A17: *This has been rephrased (lines 165-168): "To control for the impact of each confounding factor using Grapes, we split our data into two roughly equal-sized groups according to protein length, expression level, average RSA, and average intrinsic disorder and reran the analysis within the "high" and "low" groups, combining probabilities from the two analyses using the weighted Z-method [54]"*

To do so, we first assessed the correlation of gene age with the rates of molecular evolution in distinct categories of genes, according to a putative confounding factor.

-> not totally clear, phrased like this. Am I correct to understand this: We first sorted genes into classes, according to a putative confounding factor, and then assessed the correlation of gene age with the rates of molecular evolution within each class?

***A18:** We thank the reviewer for this suggestion. However, we have decided to remove this part of the results section to minimize repetition. This analysis is now clarified in lines 165-168 (see A17).*

page 5, lines 59: tau = -8.48 ??
not clear why there are two p-values and two tau values. perhaps be more explicit.

***A19:** The value of tau had a typo; this is now corrected. Moreover, we have clarified the meaning of the two p-values (lines 152-155).*

- 'controls for confounding effects are only considering two categories (low and high)'
is this control sufficiently tight? Isn't there still some gene age / gene length (or other confounding factor) stratification within each class? In fact, this point could be tested internally: do you still see a significant correlation between gene age and e.g. gene length within the high or within the low class ? An alternative control would have relied on a bins of differing gene ages that are matched for their underlying distribution for gene length (or for any other counfounding factor), although it is not clear to me whether it would be easy to do such matched subsampling in the present case while still guaranteeing sufficiently large sample size within each bin. Of note, the Authors are also using an alternative approach, using linear regression, based on Huang, 2021, which makes their analysis much less dependent on this single control experiment.

***A20:** We thank the reviewer for these suggestions. As previously noted, in the revised manuscript, we decided to develop and emphasize the analyses based on MK regression, as it addresses several of the concerns of the stratification approach. We believe that the two statistical approaches complement each other: the MK regression addresses the issue of jointly accounting for multiple confounding factors without discretization and data subsetting. Yet, it comes at the cost of not modelling the DFE, which the stratification approach permits, allowing better estimates of ω_a. Moreover, we have performed three additional analyses to assess the effect of the residual variance of the co-factor within each "high" and "low" group". Our findings suggest that the effect of gene age on ω_a is independent of the co-factor (see our response to reviewer #1, A2).*

- 'We first examined which functions are encoded by young genes in A. thaliana and D. melanogaster...'
-> why only young genes? Wouldn't that make sense to contrast young versus old ? Exactly like the correlation between gene length and gene age was verified before controlling for gene length, earlier in the manuscript, wouldn't that make sense here to test whether some functions are over- or under-represented in young genes versus old genes, before trying to control for this?

***A21:** The idea was to assess which functions were encoded by young genes to verify whether the gene function drove the higher rates of adaptive evolution. We have focused on young genes to ensure enough genes in each age class to contrast young versus old in the GO categories. Unfortunately, the number of annotated young genes was very small, thus not allowing a rigorous analysis of the functions that are under- or over-represented by young or old genes.*

- 'When looking at omega_na, our analyses revealed a strong influence of gene age in most functions analysed in both species, where young genes present higher rates of non-adaptive substitutions.'
-> phrasing is slightly ambiguous. Strong influence of gene age on omega_na within most functional classes?

***A22:** We have rephrased as suggested (lines 261-264): "When looking at the non-adaptive rate of evolution, our analyses revealed a strong influence of gene age in ω_{na} within most functions analysed in both species, where young genes present higher rates of non-adaptive substitutions".*

References

1. *Huang Y-F. Dissecting Genomic Determinants of Positive Selection with an Evolution-Guided Regression Model. Molecular Biology and Evolution, 39(1), msab291.*

2. *Cox DR, Snell EJ. Analysis of Binary Data (2nd ed.). London: Chapman and Hall; 1970.*
