# Supplementary material for: Strong evidence for the adaptive walk model of gene evolution in Drosophila and Arabidopsis
Source: PLoS Biol. 2022 Sep 13;20(9):e3001775. doi: 10.1371/journal.pbio.3001775 (PMC9470001; doi:10.1371/journal.pbio.3001775)
Supplement: Attachment — Submitted filename: Response_reviewers_r2.pdf [file pbio.3001775.s057.pdf]

REVIEWERS' COMMENTS:

Reviewer #1:

The authors have addressed my previous concerns about the analyses and have vastly improved the manuscript.

We thank the reviewer for the positive comments about the manuscript.

I just have a few minor comments to help improve presentation:

1) Lines 245-250: This part was a little unclear. I think a sentence or two are needed toward the beginning of this paragraph giving an overview of what the authors are doing. My understanding is that the authors wish to control for the effect of protein function as a potential confounder on the relationship between omega and gene age. So, they split the genes into different functional categories based on GO terms. Then, within a given GO term, the genes were further divided into different age categories. Grapes was then used to estimate omega. Consider giving an overview like this before delving into the details of how the groups were assigned, etc.

***A1:** We thank the reviewer for their suggestion. We have followed the reviewer's suggestion and rephrased the beginning of this paragraph (lines 251-258):*

"To correct for the potential bias of protein function in the relationship between the rate of adaptive evolution and gene age, we split the genes into different functional categories based on GO terms. To simultaneously control for the effect of gene age, we further divided the genes into three age categories, trying to keep a similar number of genes in each (see Material and Methods). Grapes was then used to estimate ω, ω_{na}, and ω_a in each combined category. As some gene functions were biased towards some age categories (Figure S5), we could not do this analysis for all GO terms. We, therefore, only used the GO terms with a sufficient number of annotated genes in each age class (see Material and Methods)."

2) Figure S7: The caption here is a little unclear still. Ga is called the proportion of adaptive substitutions and later it's called the "Grantham's distance values" (when referring to the regression line). I think these are the same quantities, but it would be cleaner to use one term in the figure.

***A2:** We thank the reviewer for pointing this out. We have now clarified the legend of Figure S7 as follows:*

"Relationship between the average Grantham's distance amongst adaptive ($\overline{G_a}$) and non-adaptive ($\overline{G_{na}}$) substitutions and gene age. Each point represents the weighted average for each age category. A linear model was fitted between gene age and $\overline{G_a}$ and $\overline{G_{na}}$ values and is represented with the blue line. Statistical significance was assessed with a Pearson's correlation test and the respective correlation coefficient (R) and p-values (p) are shown in each plot."

3) Methods, section starting on line 520: While this is a little clearer than the previous version of the manuscript, I still think it's hard to follow. I might suggest re-ordering different parts. Specifically, I would first talk about the Grantham distances (like what is done up to line 526). Then, I would suggest talking about estimating omega and Grapes next (ie I would move the parts in lines 539-550 earlier). Lastly, I would talk about the details of inferring the proportion of adaptive nonsynonymous substitutions using the method of Bergman and Eyre-Walker. Right now, it's hard to know why that method is used and what it's being used for. Explicitly explaining that too would make the manuscript clearer here.

A3: *We thank the reviewer for pointing this out. We have rewritten this section to further clarify the purpose of using the method of Bergman and Eyre-Walker. However, we did not change the order of the text as their method is necessary to run Grapes. Specifically, we used their method to estimate the synonymous and non-synonymous site frequency spectrum (SFS) for each amino-acid pair, two of the parameters required to run Grapes. Grapes estimates the rate of adaptive and non-adaptive substitutions by comparing the SFS of a class of sites subject to selection (non-synonymous sites) to the SFS of a class of sites assumed to be neutral (here, synonymous sites). So, if one would want to estimate the rate of adaptive and non-adaptive substitutions for a class of genes, then the synonymous and non-synonymous SFS would just be calculated by summing the numbers of synonymous and non-synonymous codon sites at each position. However, in this analysis, we want to estimate* ω_a *and* ω_{na} *for each amino-acid pair, so, only non-synonymous polymorphisms are directly counted. Hence, we compared the non-synonymous SFS for each amino acid pair with the synonymous SFS estimated from the 4-fold degenerate sites separated by the same mutation type. For example, if we consider alanine and aspartic acid, which are separated by an A <> C mutation, we compared the non-synonymous SFS with the synonymous 4-fold degenerate sites separated only by A <> C mutations. We hope to have clarified this analysis (lines 535-539):*

"In the second analysis, we estimated the average Grantham's distance for adaptive ($\bar{G}_a$*) and non-adaptive (*$\bar{G}_{na}$*) non-synonymous substitutions within each age stratum. This analysis was performed by running Grapes across amino acid pairs separated by a single mutational step within categories of gene age. To run Grapes, we first estimated the synonymous and non-synonymous SFS using the same approach as in Bergman and Eyre-Walker [106]."*

Reviewer #2:

The authors have sufficiently addressed my concerns.

We thank the reviewer for their comments.

Reviewer #3:

The Authors have addressed my comments. I find the experiment controlling for omega_na particularly interesting, in the idea to try to discriminate between the adaptive walk hypothesis and the static model. I still think that more direct tests of the adaptive walk could be considered, e.g. one should see a trend for omega over time. But that's left for future work..

congrats for this beautiful manuscript.

We thank the reviewer for his enthusiastic feedback on our work.
